# Pathologist-Read vs AI-Driven Assessment of Tumor-Infiltrating Lymphocytes in Melanoma

**DOI:** 10.1001/jamanetworkopen.2025.18906

**Published:** 2025-07-03

**Authors:** Thazin N. Aung, Matthew Liu, David Su, Saba Shafi, Ceren Boyaci, Sanna Steen, Nikolaos Tsiknakis, Joan Martinez Vidal, Nigel Maher, Goran Micevic, Samuel X. Tan, Matthew D. Vesely, Saeed Nourmohammadi, Yalai Bai, Dijana Djureinovic, Pok Fai Wong, Katherine Bates, Nay N. N. Chan, Niki Gavrielatou, Mengni He, Sneha Burela, Robert Barna, Martina Bosic, Konstantin Bräutigam, Irineu Illabochaca, Zhou Chenhao, Joao Gama, Bianca Kreis, Reka Mohacsi, Nir Pillar, Joao Pinto, Christos Poulios, Maria Angeliki Toli, Evangelos Tzoras, Yadriel Bracero, Francesca Bosisio, Gábor Cserni, Alis Dema, Francesco Fortarezza, Mercedes Solorzano Gonzalez, Irene Gullo, Francisco Javier Queipo Gutiérrez, Ezgi Hacihasanoglu, Viktor Jovic, Bianca Lazar, Maria Olinca, Christina Neppl, Rui Caetano Oliveira, Federica Pezzuto, Daniel Gomes Pinto, Vanda Plotar, Ovidiu Pop, Tilman Rau, Kristijan Skok, Wenwen Sun, Ezgi Dicle Serbes, Wiebke Solass, Olga Stanowska, Marcell Szasz, Krzysztof Szymonski, Franziska Thimm, Danielle Vignati, Alon Vigdorovits, Victor Prieto, Tobias Sinnberg, James Wilmott, Shawn Cowper, Jonathan Warrell, Yvonne Saenger, Johan Hartman, Jasmine Plummer, Iman Osman, David L. Rimm, Balazs Acs

**Affiliations:** 1 Department of Pathology, Yale University School of Medicine, New Haven, Connecticut; 2 Yale Cancer Center, New Haven, Connecticut; 3 Department of Pathology, The Ohio State University Wexner Medical Center, Columbus; 4 Department of Clinical Pathology and Cancer Diagnostics, Karolinska University Hospital, Stockholm, Sweden; 5 Department of Oncology and Pathology, Karolinska Institutet, Stockholm, Sweden; 6 Medical Oncology Department, Hospital Clínic, Barcelona, Spain, Barcelona, Spain; 7 Melanoma Institute Australia, The University of Sydney, Sydney, New South Wales, Australia; 8 Frazer Institute, University of Queensland, Brisbane, Queensland, Australia; 9 Mayo Clinic, Scottsdale, Arizona; 10 ANAPATMOL Research Center, Victor Babes University of Medicine and Pharmacy, Timisoara, Romania; 11 University of Belgrade, Faculty of Medicine, Institute of Pathology “Prof. Dr Djordje Joannovic”; 12 Centre for Evolution and Cancer, The Institute of Cancer Research, London, United Kingdom; 13 Department of Dermatology, NYU Grossman School of Medicine, New York, New York; 14 Pathology Department, Faculty of Medicine of the University of Coimbra, Centro Hospitalar e Universitário de Coimbra, Coimbra, Portugal; 15 Institute of Pathology, General Hospital Leoben, Leoben, Austria; 16 Department of Medical Oncology, Semmelweis University, Hungary; 17 Department of Pathology, Hadassah Hebrew University Medical Center, Jerusalem, Israel; 18 Pathology Laboratory, Institute of Molecular Pathology and Immunology of University of Porto (IPATIMUP), Porto, Portugal; 19 European Society of Pathology, Brussels, Belgium; 20 Montefiore Einstein Comprehensive Cancer Center, Albert Einstein College of Medicine, Bronx, New York; 21 Translational Cell & Tissue Research, Faculty of Medicine, KU Leuven, Belgium; 22 Department of Pathology, Bács-Kiskun County Teaching Hospital, Department of Pathology, Albert Szent-György Faculty of Medicine University of Szeged, Hungary; 23 University Hospital of Padova, Surgical Pathology and Cytopathology Unit, Padova, Italy.; 24 Department of Pathology, Complexo Hospitalario Universitario A Coruña, A Coruña, Spain; 25 Department of Pathology, Unidade Local de Saúde São João, Porto, Portugal; 26 Department of Pathology, Faculty of Medicine of the University of Porto (FMUP), Porto, Portugal; 27 Instituto de Investigação e Inovação em Saúde (i3S), Porto, Portugal.; 28 Department of Pathology, Yeditepe University, Turkey; 29 Pathophysiology Department, University of Medicine, Pharmacy, Sciences and Technology of Targu Mures, Targu Mures, Romania; 30 Department of Pathology, University Medicine and Pharmacy “Carol Davila”, Bucharest, Romania; 31 Institute of Pathology, University Hospital Düsseldorf, Germany; 32 Department of Cardiac, Thoracic, Vascular Sciences and Public Health, University of Padova, Padova, Italy; 33 Department of Pathology, NOVA Medical School, Lisboa, Portugal; 34 Surgical and Molecular Tumor-pathology, National Institute of Oncology, Budapest, Hungary; 35 Morphological Sciences, University of Oradea, Faculty of Medicine and Pharmacy, Oradea, Romania; 36 Diagnostic and Research Institute of Pathology, Medical University of Graz, Graz, Austria; 37 Institute of Biomedical Sciences, Faculty of Medicine, University of Maribor, Maribor, Slovenia; 38 Ankara University Medical School, Ankara, Turkey; 39 Van Research and Training Hospital, Van, Turkey; 40 Institute of Tissue Medicine and Pathology Bern, University Bern, Switzerland; 41 Pathomorphology Department, Jagiellonian University Medical College, Cracow, Poland; 42 Department of Pathology and Laboratory Medicine, The University of Texas MD Anderson Cancer Center, Houston, Texas; 43 Department of Dermatology, The University of Texas MD Anderson Cancer Center, Houston, Texas; 44 Center for Dermatooncology, Department of Dermatology, Eberhard Karls University of Tübingen, Tübingen, Germany; 45 Department of Dermatology, Venereology and Allergology, Charité - Universitätsmedizin Berlin, Berlin, Germany; 46 Department of Molecular Biophysics and Biochemistry, Program in Computational Biology and Bioinformatics, Yale University, New Haven, Connecticut; 47 NEC Laboratories America, Princeton Office, Princeton, New Jersey; 48 Center for Spatial Omics, St Jude Children’s Research Hospital, Memphis, Tennessee

## Abstract

**Importance:**

Tumor-infiltrating lymphocytes (TILs) are a provocative biomarker in melanoma, influencing diagnosis, prognosis, and immunotherapy outcomes; however, traditional pathologist-read TIL assessment on hematoxylin and eosin–stained slides is prone to interobserver variability, leading to inconsistent clinical decisions. Therefore, development of newer TIL scoring approaches that produce more reliable and consistent readouts is important.

**Objective:**

To evaluate the analytical and clinical validity of a machine learning algorithm for TIL quantification in melanoma compared with traditional pathologist-read methods.

**Design, Setting, and Participants:**

This multioperator, global, multi-institutional prognostic study compared TIL scoring reproducibility between traditional pathologist-read methods and an artificial intelligence (AI)-driven approach. The study was conducted using retrospective cohorts of patients with melanoma between January 2022 and June 2023 across 45 institutions, with tissue evaluated by participants from academic, clinical, and research institutions. Participants were selected to ensure diverse expertise and professional backgrounds.

**Main Outcomes and Measures:**

Intraclass correlation coefficient (ICC) values were calculated for the manual and AI-assisted arms using log-transformed data. Kendall *W* values were calculated for Clark scores (brisk = 3, nonbrisk = 2, and sparse = 1). Reliabilities of ICC and *W* values were classified as moderate (0.40-0.60), good (0.61-0.80), or excellent (>0.80). AI TIL measurements were dichotomized using the 16.6 and median cutoffs. Univariable and multivariable Cox regression analyses assessed the prognostic value of TIL scores adjusted for clinicopathologic variables.

**Results:**

There were 111 patients with melanoma in the independent testing cohort (median [range] age at diagnosis, 61.0 [25.0-87.0] years; 56 [50.5%] male) who contributed melanoma whole tissue sections. A total of 98 participants evaluated TILs on 60 hematoxylin and eosin–stained melanoma tissue sections. All 40 participants in the manual arm were pathologists, while the AI-assisted arm included 11 pathologists and 47 nonpathologists (scientists). The AI algorithm demonstrated superior reproducibility, with ICCs higher than 0.90 for all machine learning TIL variables, significantly outperforming manual assessments (ICC, 0.61 for AI-derived stromal TILs vs Kendall *W*, 0.44 for manual Clark TIL scoring). AI-based TIL scores showed prognostic associations with patient outcomes (n = 111) using the median cutoff approach with a hazard ratio (HR) of 0.45 (95% CI, 0.26-0.80; *P* = .005), and using the cutoff of 16.6, with an HR of 0.56 (95% CI, 0.32-0.98; *P* = .04).

**Conclusions and Relevance:**

In this prognostic study of TIL quantification in melanoma, the AI algorithm demonstrated superior reproducibility and prognostic associations compared with traditional methods. Although the retrospective nature of the cohorts limits demonstration of clinical utility, the publicly available dataset and open-source AI tool offer a foundation for future validation and integration into melanoma management.

## Key Points

**Question:**

Is the use of a machine learning algorithm for tumor-infiltrating lymphocyte (TIL) quantification in melanoma associated with improved reproducibility and prognostic validity compared with traditional pathologist-read methods?

**Findings:**

In this prognostic study across 45 institutions with 98 participants, the artificial intelligence (AI) algorithm achieved high reproducibility for all machine learning TIL variables, significantly outperforming traditional pathologist-read methods. AI-based TIL scores also showed prognostic associations with patient outcomes.

**Meaning:**

These findings suggest that an AI-driven TIL quantification tool may provide consistent, reliable assessments with a strong potential for clinical integration, offering a robust alternative to traditional methods.

## Teaser

This prognostic study compares the analytical and clinical validity of a machine learning algorithm with traditional pathologist-read methods for quantification of tumor-infiltrating lymphocytes in melanoma.

## Introduction

Tumor-infiltrating lymphocytes (TILs) play a crucial role in cancer biology as key components of the tumor microenvironment. They serve as important indicators of the immune system response to malignancy, with their presence associated with favorable clinical outcomes.^1^ This prognostic significance is evident in breast, ovarian, and colorectal cancers, for which the density, type, and spatial distribution of TILs are linked to prognosis and treatment response.^2,3,4,5,6,7,8^ TILs also hold predictive value across various cancers, guiding therapeutic decision-making, especially for the selection and optimization of immunotherapies.^2,3^ This predictive value is especially pronounced in melanoma, with TILs emerging as critical biomarkers for predicting responses to immune checkpoint inhibitors.^4,5,6^

High TIL levels in melanoma are strongly associated with improved outcomes, and TIL assessment, if not common, has become a routine practice for melanoma in many institutions.^7,8,9,10,11^Traditionally, the assessment of TILs has been performed by pathologists through visual examination of hematoxylin and eosin (H&E)-stained tissue sections.^12,13^ While this method provides valuable insights, it is often hampered by interobserver variability, subjectivity, and lack of widely accepted consensus guidelines, leading to inconsistent and unreliable results.^14,15^ The accuracy of TIL scoring by pathologists rests on the rigor of the standardized methods currently used. The Clark grading system ranks TILs as sparse, nonbrisk, or brisk from the intratumoral region,^9^ whereas in breast cancer, a method endorsed by the International TILS Working Group (TIL-WG) recommends scoring stromal and intratumoral TILs separately by percentage.^12,13^ Both methods are subjective, semiquantitative, and performed visually on H&E slides by pathologists. These shortcomings have spurred efforts to develop newer TIL scoring approaches that produce more reliable and objective readouts.

Advances in artificial intelligence (AI) and machine learning have improved the accurate and consistent quantification of TILs, demonstrating clinical validity across studies.^16,17,18^ However, the analytical performance of AI methods may be influenced by variability arising from tissue handling factors or operator dependency. To address these challenges, we developed a streamlined machine learning algorithm to manage the analytical variability associated with tissue processing while incorporating input from human operators during analysis. This is the first study, to our knowledge, to evaluate analytical validity in a large, multioperator, and multi-institutional setting, closely mimicking clinical practice, using a previously validated AI tool for TIL scoring in melanoma. Prior work by our team with the AI-based eTIL algorithm demonstrated its robustness in assessing TILs across diverse melanoma and lung cohorts.^5,12,13,19^ In these studies, the eTIL algorithm demonstrated high precision in identifying and quantifying TILs across tissue variations, establishing its analytical validity and potential as a standardized clinical tool. The present project evaluated its analytical and clinical validity in melanoma, comparing AI-driven TIL scoring across multiple operators with traditional TIL scoring by pathologists and assessing its consistency and objectivity in TIL assessment.

## Methods

### Patient Cohorts

The study consisted of 2 melanoma cohorts (total N = 208). The training cohort from the Melanoma Institute of Australia (MIA) included 125 tumor whole tissue sections (WTSs) of cutaneous melanomas and matching metastatic lymph nodes from 97 patients diagnosed with melanoma between 1998 and 2019, with a median (IQR) follow-up of 69.8 (30.6-99.7) months. We excluded 22 WTS images due to insufficient tissue content and poor image quality, such as blurriness. Exclusion criteria included a WTS with an area less than 0.5 mm^2^ or containing less than 50% tumor cells. These samples were excluded because regions with low tumor cell content may be dominated by stromal or necrotic areas, which can confound analysis and reduce the interpretability of TIL-associated features. Consequently, 103 WTS images were included as the training cohort (accession No., S-BIAD470). The study was approved by the Sydney Local Health District (RPAH Zone) Human Research Ethics Committee, which granted a waiver of informed consent because the study used archival, deidentified specimens and posed no more than minimal risk to participants. The testing cohort from Yale University consisted of 135 patients diagnosed with melanoma between 1981 and 2010, with a median (IQR) follow-up of 66.3 (33.5-150.2) months. Of these, 24 samples were excluded due to insufficient tissue content and poor image quality. The remaining 111 samples were assessed by our AI-driven method and manually evaluated by a single pathologist (S.B.) as brisk, nonbrisk, and sparse (Clark system), as well as stromal TIL (sTIL) percentage scores (TIL-WG system). Patients provided written informed consent where applicable; for samples collected before 1995, the Yale Human Investigation Committee approved the study and granted a waiver of informed consent because the research involved minimal risk, and the use of deidentified, archived tissue made recontacting patients impracticable. The Aperio Scan Scope XT platform (Leica Biosystems) was used to digitize the H&E-stained slides from both training and testing cohorts at a magnification of ×20 with a pixel size of 0.4986 μm by 0.4986 μm. The images were initially in SVS format and subsequently converted to OME−TIFF after undergoing color normalization. The clinicopathologic characteristics for both cohorts are presented in Table 1. This prognostic study followed the Standards for Reporting of Diagnostic Accuracy (STARD) reporting guideline.

**Table 1.** Clinicopathologic Features of the Cohorts Included in This Study

Characteristic	Patients, No. (%)	
	Melanoma Institute Australia (training) cohort (n = 97)	Yale (testing) cohort (n = 111)
Age at diagnosis, median (range), y	NA	61.0 (25.0-87.0)
Follow-up, median (IQR), mo	69.8 (30.6-99.7)	66.3 (33.5-150.2)
Sex		
Female	NA	55 (49.5)
Male	NA	56 (50.5)
Core type		
Primary	NA	109 (98.2)
Metastasis	NA	2 (1.8)
Cancer stage		
I	NA	83 (74.8)
II	NA	4 (3.6)
III	NA	11 (9.9)
IV	NA	2 (1.8)
NA	NA	11 (9.9)
Clark level		
II	NA	6 (5.4)
III	11 (11.3)	36 (32.4)
IV	62 (63.9)	55 (49.6)
V	22 (22.7)	13 (11.7)
NA	2 (2.1)	1 (0.9)
Tumor-infiltrating lymphocytes		
Sparse	NA	6 (5.4)
Brisk or diffuse	NA	20 (18.0)
NA	NA	2 (1.8)
Nonbrisk or nondiffuse	NA	67 (60.4)
Sparse	NA	16 (14.4)
AJCC 7th edition stage		
IIA	23 (23.7)	NA
IIA/IIB	5 (5.2)	NA
IIB	16 (16.5)	NA
IIB/IIC	2 (2.1)	NA
IIC	10 (10.3)	NA
IIIA	13 (13.4)	NA
IIIA/IIIB	1 (1.0)	NA
IIIB	10 (10.3)	NA
IIIB/IIIC	1 (1.0)	NA
IIIC	16 (16.5)	NA

Abbreviations: AJCC 7^th^ edition, *American Joint Committee on Cancer Staging Manual Seventh Edition*; NA, not available.

### Image Selection for Interobserver Assessment

From the testing cohort, we selected 60 images of cutaneous melanoma WTS to assess interobserver variability of TIL scoring for both pathologist-read and AI assessments. These images were chosen to represent various morphological features, mimicking daily clinical practice to challenge the participants and the algorithm. The images were distributed among participants for pathologist-read and AI-assisted assessments of TIL percentages. The number of images was selected to balance the need for diversity and statistical relevance with participant workload and feasibility. Each participant received identical image sets to ensure consistency in evaluation.

### Participant Recruitment

We advertised the study on professional platforms, including LinkedIn, X (formerly Twitter), the TIL-WG ^20^ and specialized pathologist forums. The call for participants emphasized the importance of diverse expertise in evaluating TIL assessment methods. Participants included pathologists with MD and MD-PhD qualifications (for both manual and AI arms), and scientists with PhD, MSc, and BSc qualifications working in translational research (for the AI arm). To ensure consistency and reliability in TIL assessment, all participants were required to complete the US Food and Drug Administration (FDA) TIL assessment course and obtain a certificate of completion.^20^ Although focused on breast cancer, this course included melanoma related details for the present study. This step standardized the evaluation process and ensured uniform understanding of TIL assessment. Only certified participants received the set of 60 normalized images for assessment. Participants also received detailed study materials, including detailed instructions for manual or AI-assisted scoring, setup instructions for an open-source image analysis tool (QuPath),including a download link for the software, access to the FDA TILs Continuing Medical Education resources, an AI algorithm (trained on normalized images from the training cohort, n = 103), normalized images from the testing cohort (n = 60), and additional detailed instructions specific to their assigned study arm.

### AI Algorithm and Image Analysis

Members of our team previously validated the cell classifier NN_192^21^ and its prognostic significance using multi-institutional cohorts.^14,22,23,24^ However, NN_192 is user-dependent, requiring operators to estimate the stain vector for each image. Incorrect estimation can lead to inaccurate cell detection and results. To address this limitation, we implemented major updates and introduced Artificial Neural Network Multilayer Perceptron (ANN_MLP) in an updated algorithm (ANNMAR_24), which uses stain vector normalized images. This approach minimizes subjectivity and improves consistency. ANN_MLP distinguishes tumor, stromal, immune, and other cells (melanocytic cells, necrotic or apoptotic cells, red blood cells, polymorphonuclear leukocytes, and staining artifacts). Details on stain-vector normalization workflow (eFigure 1 in Supplement 1) and the algorithm development are in the eMethods in Supplement 1.^22^ The algorithm learns to recognize unique features of each cell type based on morphological characteristics and staining patterns on color normalized WTSs. From this, machine-derived TIL variables, including the percentage of electronic TILs (eTILs), electronic-total TILs (etTILs), electronic-stromal TILs (esTILs), electronic-area TILs (eaTILs), and electronic-area-stromal TILs (easTILs) are calculated^23,24^ (eMethods in Supplement 1).

### Algorithm Performance Evaluation

To evaluate our algorithm’s performance, we calculated the F1 score, which balances precision and recall (eMethods in Supplement 1). The ground truth was based on a board-certified pathologist (B.A.) annotation. We also assessed the prognostic performance of the algorithm in an independent cohort of WTSs from Yale University. The previous algorithm showed a correlation between high TIL presence and improved outcomes.^21,23^ which the updated version aimed to replicate, confirming the association between high TIL counts and favorable prognosis.

### AI TIL Scoring

Participants in the AI arm were provided a set of instructions summarized as follows: (1) inspect the image, (2) annotate the tumor, and (3) run the script to generate TIL variable scores. All participants completed the FDA TIL scoring course before accessing the data. Full copies of the instructions are in the eData in eAppendix 1 and eAppendix 2 in Supplement 1.

### Pathologist-Read TIL Scoring

Pathologists in the manual arm used the same images as the AI arm used, and they followed these instructions: (1) inspect the image, (2) identify the tumor area, (3) determine the type and the localization of inflammatory infiltrate, (4) provide sTIL score (percentage of sTILs) following TIL-WG guidelines,^25^ and (5) provide the intratumor TIL score following the Clark system.^26,27^ All participants completed the FDA TIL scoring course before accessing the data. A full copy of the instructions is in Supplement 1.

### Computational Infrastructure and Workflow Standardization

To ensure consistency, reproducibility, and generalizability across sites, we implemented a standardized computational workflow using accessible, open-source tools. Image analysis, including cell detection and classification, was performed using QuPath (version 0.4.3) with the built-in ANN_MLP classifier. Annotations used for training were reviewed by a board-certified pathologist to ensure accuracy. Stain normalization was conducted using a modified Macenko^22^ algorithm in MATLAB, applying uniform stain vectors derived from a reference image to all tissue patches for consistent color normalization. The pipeline was compatible with both local workstations and high-performance computing environments. Additional technical details are provided in the eMethods in Supplement 1.

### Statistical Analysis

Interobserver variability was analyzed separately for AI-assisted and manual assessment arms. Intraclass correlation coefficient (ICC) values were calculated for the manual (sTILs, percentage) and AI-assisted arms using log-transformed data in Python (Pingouin library).^28^ The Kendall *W* value for Clark scores (brisk = 3, nonbrisk = 2, and sparse = 1) was calculated using R (irr package) (R Project for Statistical Computing).^29^ Reliabilities of ICC and *W* values were classified as moderate (0.40-0.60), good (0.61-0.80), or excellent (>0.80).^30^ Comparing ICC and *W* values between methods determined the consistency of the AI algorithm relative to human experts. Disease-specific survival was time from diagnosis to melanoma events. AI TIL measurements were dichotomized using the 16.6 and median cutoffs.^21^ Manual scoring used 10% and 30% cutoffs, as established in prior studies.^31,32,33,34,35^ Survival differences were tested with the log-rank test. Univariable and multivariable Cox regression analyses assessed the prognostic value of TIL scores adjusted for clinicopathologic variables. Schoenfeld residuals validated proportional hazard assumptions for all machine-derived TILs variables. Statistical analyses used survminer^36^ and survival^37^ packages in R. Statistical significance was considered a 2-sided *P* < .05.

## Results

There were 111 patients with melanoma in the testing cohort used to test analytical and clinical validity. The female (55 [49.5%]) to male (56 [50.5%]) ratio was approximately 1:1. The median [range] age at diagnosis was 61.0 [25.0-87.0] years. Most of the patients had stage I disease (Table 1).

### Participant Enrollment and Retention

**Figure 1.**
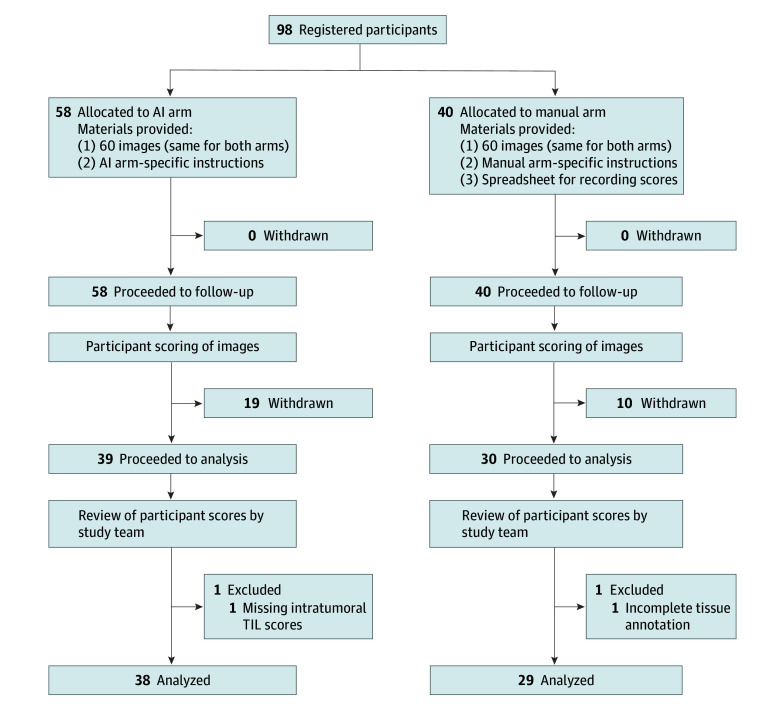
Diagram for the International Round-Robin Study TIL represents tumor-infiltrating lymphocyte.

A total of 98 participants registered for the study to score 60 digitized WTSs of melanoma, with 58 in the AI arm and 40 in the manual arm, representing 45 institutions globally. Assignments were based on professional backgrounds: the AI arm included 11 pathologists and scientists, while the manual arm consisted entirely of pathologists. This ensured diverse representation of techniques and interpretations from various health care and research settings. The open recruitment approach was intentionally designed to capture analytical variability in TIL scoring practices closest to clinical routine and to enhance the generalizability of findings. During the study, 29 participants dropped out, leaving 69 active participants. In the AI arm, 1 of 39 participants was excluded for incomplete tissue annotation. In the manual arm, 1 participant was excluded for providing only sTIL scores without intratumoral TIL scores. After exclusions, participants represented 39 unique institutions. Dropout rates were recorded to ensure transparency and to understand participant challenges. The diagram in Figure 1 illustrates participant recruitment.

### Analytical Performance of the ANNMAR_24 Algorithm

The ANNMAR_24 algorithm was evaluated using F1 scores across 5 images. The F1 score combines precision, measuring accuracy in identification, and recall, measuring correct identifications relative to all instances. The algorithm performed best in classifying tumor cells, with an F1 score of 0.80, recall of 0.89, and precision of 0.72. For immune cells, the F1 score was 0.70, with recall of 0.60 and precision of 0.80. Stromal cells had a moderate F1 score of 0.53, recall of 0.73, and precision of 0.45. The other cells category showed the poorest performance, with an F1 score of 0.14, precision of 0.52, and recall of 0.09 (eFigure 2 in Supplement 1).

### Analytical Validity

**Figure 2.**
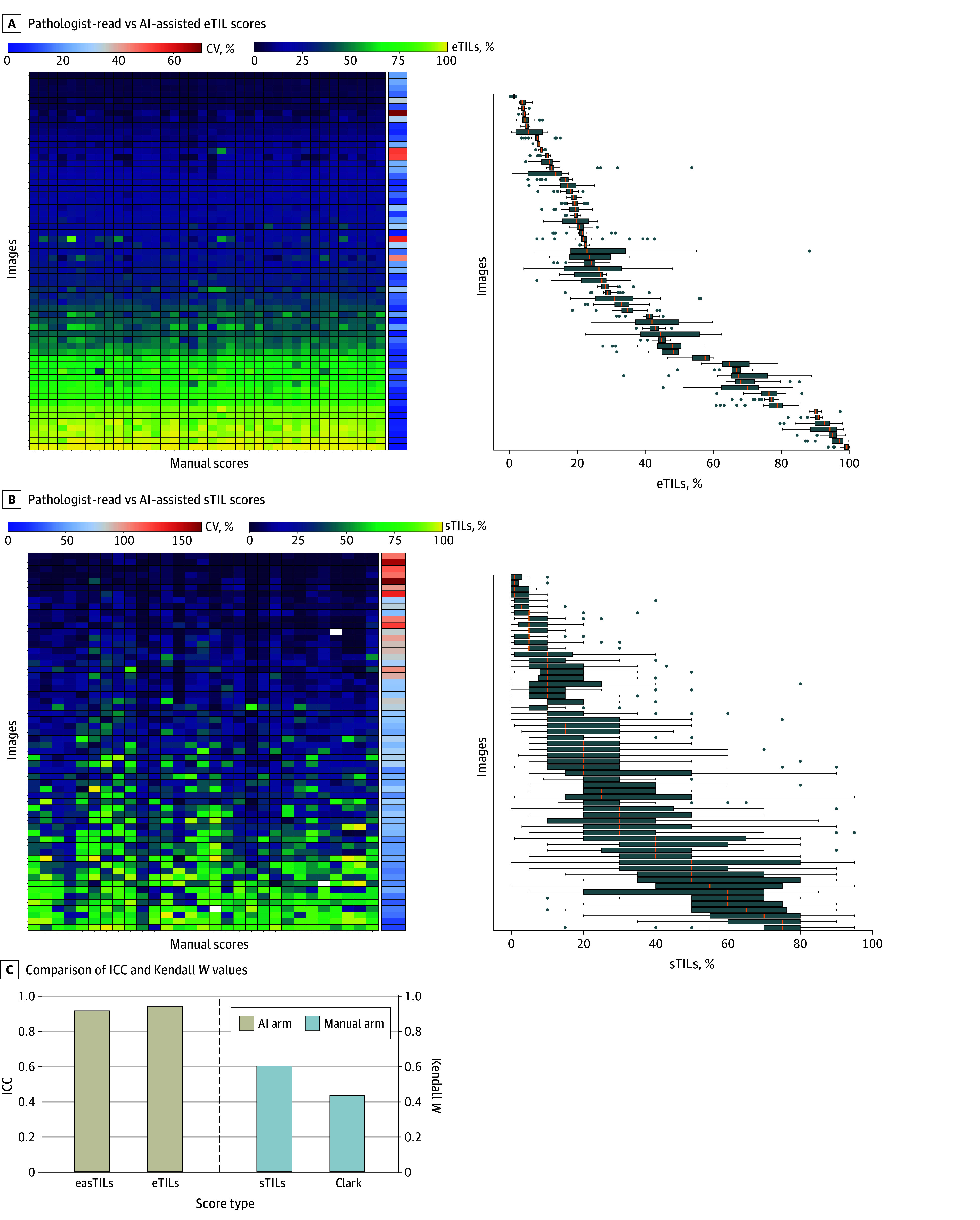
Interobserver Variability in AI-Assisted vs Manual Tumor-Infiltrating Lymphocyte (TIL) Scoring Heatmaps and corresponding box plots display variability in selected TIL variables. (A) electronic TIL (eTILs) score from the AI arm demonstrating lower operator variability compared with (B) stromal TIL (sTILs) score from the manual arm. The vertical axes for each heatmap-box plot set are ordered by their respective median values. Concordance statistics are provided for each variable in both arms. CV indicates coefficient of variation. (C) Comparison of intraclass correlation coefficient (ICC) values (for electronic-area-stromal TIL [easTILs], eTILs, and sTILs scores) and Kendall *W* (for Clark TIL scoring) values between the AI arm and the manual pathologist-read TIL scoring arm.

We compared the scoring of 60 images between participants in the AI-assisted and pathologist-read arms. The calculation methods for the 5 machine-derived TIL variables are detailed in the eMethods and eFigure 3 in Supplement 1, which was adapted with permission from a previously published version.^23^ AI-assisted assessments, particularly percentages of eTILs (Figure 2A) and easTILs (eFigure 4B in Supplement 1), showed significantly higher operator concordance compared with pathologist-read assessments (Figure 2C). The AI-assisted group demonstrated very high ICC values: 0.92 (95% CI, 0.89-0.94) for easTILs percentage, 0.92 (95% CI, 0.89-0.94) for eaTILs per square millimeter, 0.91 (95% CI, 0.87-0.94) for esTILs percentage, 0.94 (95% CI, 0.92-0.96) for eTILs percentage, and 0.92 (95% CI, 0.89-0.94) for etTILs percentage (Figure 2A; eFigure 4A-D in Supplement 1). Pathologists in the manual arm showed good reproducibility for sTIL scoring per TIL-WG guidelines, with an ICC of 0.60 (95% CI, 0.51-0.70) (Figure 2C). However, the intratumoral TIL scoring (Clark system) demonstrated low agreement (Kendall *W* = 0.44) (Figure 2C). Since the AI-assisted group included participants with diverse educational backgrounds, not all board-certified pathologists (eTable 1 in Supplement 1), we further evaluated the interobserver variability between board-certified pathologists (n = 11) and non–board-certified participants (n = 27) in the AI-assisted arm. ICC values were comparable (eFigure 5 in Supplement 1), highlighting high reproducibility across groups regardless of educational background.

### Clinical Validity

**Figure 3.**
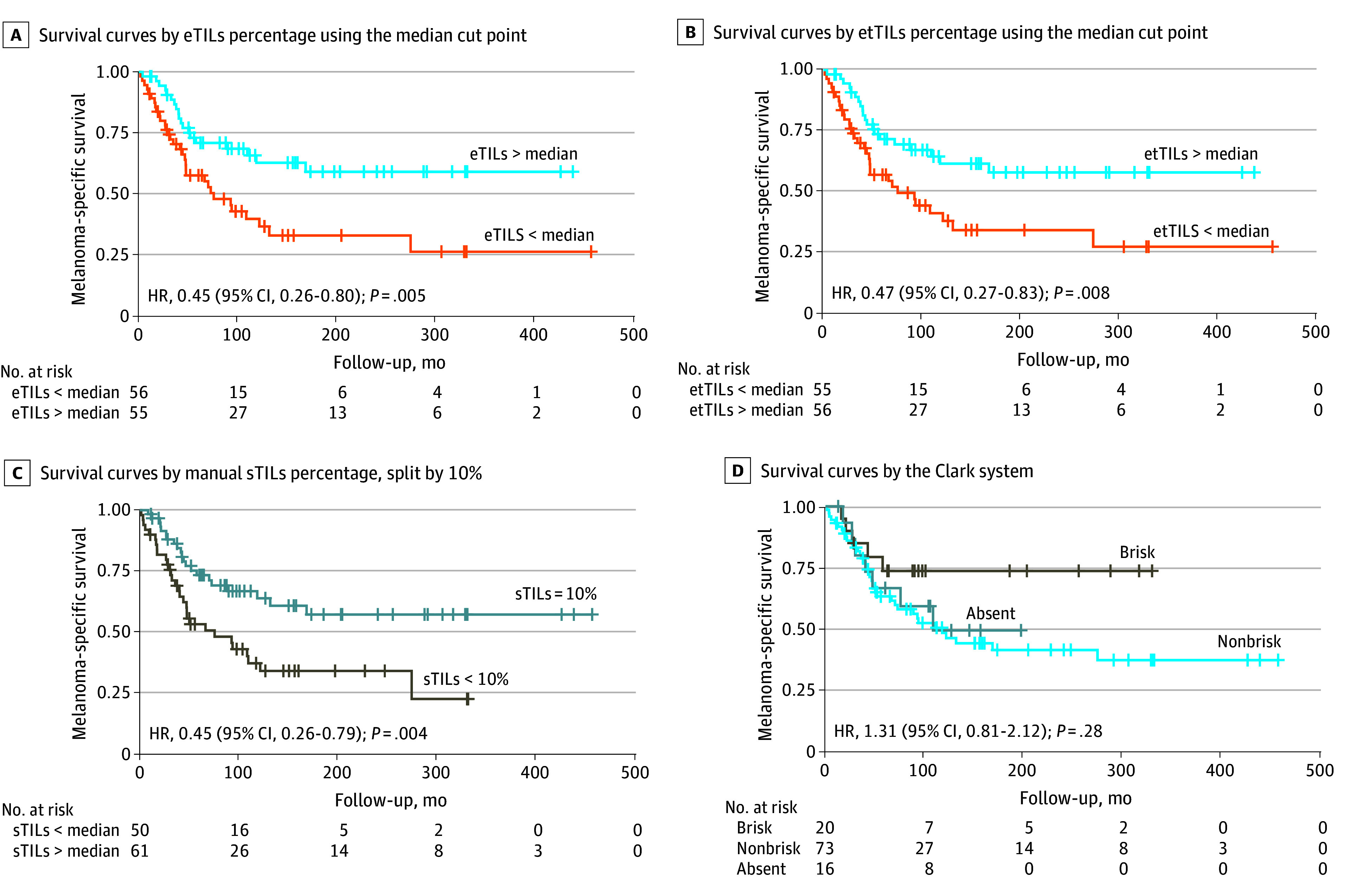
Comparative Survival Analysis Based on Tumor-Infiltrating Lymphocyte (TIL) Scoring Methods eTILs represents electronic TIL score; etTILs, electronic-total TIL score; sTILs, stromal TIL score; HR, hazard ratio.

The evaluation of the prognostic performance of our algorithm across 5 machine-derived TIL variables demonstrated associations in the independent testing cohort (n = 111). Machine-derived TIL scores (median cutoff) had a hazard ratio (HR) of 0.45 (95% CI, 0.26-0.80; *P* = .005) (Figure 3A). Using the previously published cutoff of eTILs percentage at 16.6,^21^ our machine-derived TIL scores showed an HR of 0.56 (95% CI, 0.32-0.98; *P* = .04) (eFigure 6A in Supplement 1). However, the easTILs percentage, which mirrors manual pathologist TIL assessments, was not associated with clinical outcomes (HR, 0.91 [95% CI, 0.53-1.58]; *P* = .74) (eFigure 6B in Supplement 1). The etTILs percentage (Figure 3B) was associated with clinical outcomes (HR, 0.47 [95% CI, 0.27-0.83]; *P* = .008). In contrast, eaTILs (per square millimeter) (HR, 0.91 [95% CI, 0.53-1.58]; *P* = .74) and the esTILs percentage (HR, 0.88 [95% CI, 0.51-1.53]; *P* = .64) showed no association (eFigure 6C and D in Supplement 1). For manually assessed sTILs percentages, there was an association with clinical outcomes at a 10% cutoff (HR, 0.45 [95% CI, 0.26-0.79]; *P* = .004) (Figure 3C) but not at a 30% cutoff (HR, 0.61 [95% CI, 0.31-1.22]; *P* = .16) (eFigure 6E in Supplement 1). The Clark TIL classification (brisk, nonbrisk, and sparse) also showed no association with prognosis (HR, 1.31 [95% CI, 0.81-2.12]; *P* = .28) (Figure 3D). These results indicated that specific machine-derived TIL scores were associated with improved clinical outcomes, while manually assessed TILs and traditional classifications showed varying prognostic relevance. In multivariable analysis adjusted for sex, age, and stage, only machine-derived TIL scores and stage remained independently prognostic for melanoma specific survival (Table 2; eTable 2 in Supplement 1).

**Table 2.** Univariable and Multivariable Cox Regression Analyses of TIL Scores Assessing the Association Between Various TIL Score Cutoffs and Disease-Specific Survival^a^

TIL score type and variable	Univariable		Multivariable	
	HR (95% CI)	*P* value	HR (95% CI)	*P* value
eTILs (median cutoff)				
eTILs ≥ median	0.45 (0.26-0.8)	.005	0.53 (0.29-0.97)	.04
Male sex	NA	NA	1.52 (0.82-2.81)	.18
Age	NA	NA	1.02 (0.99-1.04)	.14
Tumor stage II	NA	NA	1.50 (0.36-6.26)	.58
Tumor stage III	NA	NA	4.45 (2.12-9.32)	<.001
Tumor stage IV	NA	NA	4.99 (1.10-22.75)	.04
easTILs (median cutoff)				
easTILs ≥ median	0.91 (0.53-1.58)	.74	0.89 (0.48-1.66)	.72
Male sex	NA	NA	1.49 (0.80-2.75)	.21
Age	NA	NA	1.01 (0.99-1.04)	.26
Tumor stage II	NA	NA	1.40 (0.33-5.92)	.65
Tumor stage III	NA	NA	4.71 (2.20-10.09)	<.001
Tumor stage IV	NA	NA	5.82 (1.27-26.70)	.02
sTILs (10% cutoff)				
sTILs ≥ 10%	0.45 (0.26-0.79)	.005	0.54 (0.29-1.01)	.06
Sex (male)	NA	NA	1.62 (0.87-3.02)	.12
Age	NA	NA	1.01 (0.99-1.04)	.33
Tumor stage II	NA	NA	1.58 (0.37-6.72)	.53
Tumor stage III	NA	NA	3.77 (1.77-8.03)	<.001
Tumor stage IV	NA	NA	4.58 (1.00-21.05)	.05
Clark system				
Nonbrisk or nondiffuse	0.74 (0.33-1.66)	.46	1.35 (0.49-3.68)	.56
Sparse	1.02 (0.21-4.91)	.98	2.45 (0.44-13.69)	.31
Male sex	NA	NA	1.46 (0.79-2.70)	.23
Age	NA	NA	1.02 (0.99-1.04)	.18
Tumor stage II	NA	NA	1.64 (0.37-7.38)	.52
Tumor stage III	NA	NA	4.85 (2.29-10.29)	<.001
Tumor stage IV	NA	NA	6.23 (1.39-27.86)	.02

Abbreviations: easTILs, electronic-area-stromal; eTILs, electronic tumor-infiltrating lymphocytes; HR, hazard ratio; NA, not available; sTILs, stromal tumor-infiltrating lymphocytes; TIL, tumor-infiltrating lymphocyte.

^a^ In multivariable models, each TIL score analyzed by its cutoff is adjusted for sex, age, and tumor stage (I-IV).

## Discussion

This large multioperator, multi-institutional diagnostic/prognostic study investigated the analytical validity of an AI tool for TIL scoring in melanoma. Specifically, we compared TIL scoring reproducibility between AI operators and pathologists. Our study shows that operators using the AI tool achieved outstanding reproducibility (Figure 2A; eFigure 4 in Supplement 1). The only possible variability among AI-arm observers was due to differences in area selection by individual operators, leading to generally consistent results across multiple operators. Examples of area selection variations are shown in eFigures 7-9 in Supplement 1. The high F1 score of the AI algorithm underscored its ability to reduce variability and enhance the reproducibility of TIL quantification, while its effective color normalization addressed limitations of previous methods,^21,22,26^ presenting a substantial advancement in melanoma diagnostics. In contrast, manual TIL scoring showed substantial interobserver variability, reflecting the subjectivity and expertise-dependence of manual assessment and guideline interpretation.

Immunotherapies, including pembrolizumab and ipilimumab, benefit only 20% of patients with stage III melanoma, while 50% achieve disease-free survival after surgery alone.^31,32,38^ The NADINA trial (Multicenter Phase 3 Trial Comparing Neoadjuvant Ipilimumab Plus Nivolumab Versus Standard Adjuvant Nivolumab in Macroscopic Stage III Melanoma) showed neoadjuvant nivolumab plus ipilimumab treatment reduced disease recurrence or death by 68% compared with standard dissection and adjuvant nivolumab in resectable melanoma.^31,32,38^ TILs could identify patients unlikely to benefit from immunotherapy. TILs have traditionally been assessed semiquantitatively by pathologists on H&E-stained slides as sparse, nonbrisk, or brisk.^27^ A 4-tier grading system for TIL distribution and density, used for decades, has shown limited prognostic value due to low reproducibility^39,40,41,42^. In breast cancer, the TIL-WG standardized the sTILs percentage scoring method and published detailed guidelines for pathologist-read visual assessment of H&E sections.^12,27^ This method has demonstrated robustness in international ring trials,^14^ and level 1 evidence supports its clinical utility, particularly in triple-negative breast cancer^43,44,45^. However, it remains unclear whether the sTILs percentage has the same potential in melanoma. In this study, we showed that the sTILs percentage in melanoma had good reproducibility among 29 pathologists. However, TIL scoring based on the Clark system showed low analytical validity.

Despite US FDA clearance of digital pathology in 2017, adoption in US laboratories remains minimal.^33^ AI-based approaches for TIL biomarkers show potential but often lack comprehensive validation, emphasizing the need for studies comparing their validity with traditional methods.^21,24,34^ Most importantly, studies comparing analytical and clinical validity of AI tools with traditional methods, such as those used by pathologists, are needed. The major strength of the present study is its scale, representing the largest, to our knowledge, international investigation of the analytical validity of an AI tool, involving 38 operators from 36 institutions. The study replicates real-life clinical practice, incorporating challenging cases with diverse participants. Notably, the AI algorithm demonstrated strong performance with minimal interoperator variability, showing that users familiar with tumor delineation can operate it effectively (eFigure 5 in Supplement 1). Additionally, we compared the analytical validity of the algorithm to that of 29 independent pathologists evaluating TILs in a parallel study arm. Our findings also support the analytical validity of the TIL-WG pathologist-based TIL scoring system.^20^ We further evaluated its performance in an independent test cohort. In this study, only the AI-based TIL scoring showed robust and independent prognostic potential in multivariable analyses, whereas pathologist-assessed TIL evaluations, including the Clark system, showed no significant prognostic value. Moreover, as validated on core and WTSs, our approach offers a standardized, reproducible method for TIL assessment, supporting its future clinical application and integration into routine practice. We are optimistic that the data presented herein, along with the user-friendly, open-source ANNMAR_24, will provide a foundation for future prospective evaluations. To facilitate the clinical adoption of AI tools in melanoma, we have made the data publicly available as a benchmark for validating AI tools against pathologist-assessed TIL scores. The open-source nature of our approach also promotes independent validation and broader clinical implementation.

### Limitations

This study has limitations. The main limitation of the study is the retrospective nature of the cohorts, which prevents demonstrating the clinical utility of our AI model for TIL scoring in melanoma. However, retrospective studies are essential for establishing AI tools before prospective trials due to cost and feasibility constraints.

## Conclusions

In this prognostic study of TILs in melanoma, we provided robust evidence of the analytical and clinical validity of an objective AI tool for TIL scoring in melanoma, demonstrating superior consistency among operators and enhanced clinical validity compared with the current semiquantitative gold standard. While traditional pathologist-read TIL evaluations offer valuable insights, our findings indicated that an advanced AI tool enhanced reproducibility of TIL scoring and improved prognostic potential.

## Notes

Supplement 1. **eFigure 1.** Refinement of color normalization algorithm for H&E WSIs **eFigure 2.** F1 score across different cell types **eFigure 3.** Schematic representation of the five tumor-infiltrating lymphocyte (TIL) variables **eFigure 4.** Interobserver variability among operators on the AI arm for additional TIL variables **eFigure 5.** Interobserver variability between operators on the AI arm, stratified by board-certified pathologists (n=11) and non-board-certified pathologists (n=27) **eFigure 6.** Comparative survival analysis based on TIL scoring methods **eFigure 7.** Example of an H&E image given to the participants, revealing differences in AI-based TILs scores across 6 selected participants (initials shown) who enrolled in the AI arm **eFigure 8.** A second example of an H&E image given to the participants, revealing differences in AI-based TILs scores across 6 selected participants (initials shown) who enrolled in the AI arm **eFigure 9.** A third example of an H&E image given to the participants, revealing differences in AI-based TILs scores across 6 selected participants (initials shown) who enrolled in the AI arm **eTable 1.** Stratification of participants in the AI-assisted arm by educational level, including the number of board-certified pathologists **eTable 2.** Univariable and multivariable cox regression analysis of tumor-infiltrating lymphocyte (TIL) scores in relation to disease-specific survival **eMethods**. Supplementary methods

Supplement 2.
Data Sharing Statement
