# Error in Byline

**DOI:** 10.1001/jamanetworkopen.2025.52301

**Published:** 2025-12-10

In the Original Investigation titled “Pathologist-Read vs AI-Driven Assessment of Tumor-Infiltrating Lymphocytes in Melanoma,”^1^ published July 3, 2025, Dr Gavrielatou’s name was misspelled in the byline. This article has been corrected.^1^

## References

[1] Aung TN, Liu M, Su D, . Pathologist-read vs AI-driven assessment of tumor-infiltrating lymphocytes in melanoma. JAMA Netw Open. 2025;8(7):e2518906. doi:10.1001/jamanetworkopen.2025.18906 40608341 PMC12232186

