# Supplementary material for: Pathologist-Read vs AI-Driven Assessment of Tumor-Infiltrating Lymphocytes in Melanoma
Source: JAMA Netw Open. 2025 Jul 3;8(7):e2518906. doi: 10.1001/jamanetworkopen.2025.18906 (PMC12232186; doi:10.1001/jamanetworkopen.2025.18906)
Supplement: Supplement 1. — eFigure 1. Refinement of color normalization algorithm for H&E WSIs eFigure 2. F1 score across different cell types eFigure 3. Schematic representation of the five tumor-infiltrating lymphocyte (TIL) variables eFigure 4. Interobserver variability among operators on the AI arm for additional TIL variables eFigure 5. Interobserver variability between operators on the AI arm, stratified by board-certified pathologists (n=11) and non-board-certified pathologists (n=27) eFigure 6. Comparative survival analysis based on TIL scoring methods eFigure 7. Example of an H&E image given to the participants, revealing differences in AI-based TILs scores across 6 selected participants (initials shown) who enrolled in the AI arm eFigure 8. A second example of an H&E image given to the participants, revealing differences in AI-based TILs scores across 6 selected participants (initials shown) who enrolled in the AI arm eFigure 9. A third example of an H&E image given to the participants, revealing differences in AI-based TILs scores across 6 selected participants (initials shown) who enrolled in the AI arm eTable 1. Stratification of participants in the AI-assisted arm by educational level, including the number of board-certified pathologists eTable 2. Univariable and multivariable cox regression analysis of tumor-infiltrating lymphocyte (TIL) scores in relation to disease-specific survival eMethods. Supplementary methods [file jamanetwopen-e2518906-s001.pdf]

Supplementary Online Content

Aung TN, Liu M, Su D, et al. Analytical and clinical validity of pathologist-read vs AI-driven assessment of tumor-infiltrating lymphocytes in melanoma. *JAMA Netw Open*. 2025;8(7):e2519020.
doi:10.1001/jamanetworkopen.2025.19020

eFigure 1. Refinement of color normalization algorithm for H&E WSIs

eFigure 2. F1 score across different cell types

eFigure 3. Schematic representation of the five tumor-infiltrating lymphocyte (TIL) variables

eFigure 4. Interobserver variability among operators on the AI arm for additional TIL variables

eFigure 5. Interobserver variability between operators on the AI arm, stratified by board-certified pathologists (n=11) and non-board-certified pathologists (n=27)

eFigure 6. Comparative survival analysis based on TIL scoring methods

eFigure 7. Example of an H&E image given to the participants, revealing differences in AI-based TILs scores across 6 selected participants (initials shown) who enrolled in the AI arm

eFigure 8. A second example of an H&E image given to the participants, revealing differences in AI-based TILs scores across 6 selected participants (initials shown) who enrolled in the AI arm

eFigure 9. A third example of an H&E image given to the participants, revealing differences in AI-based TILs scores across 6 selected participants (initials shown) who enrolled in the AI arm

eTable 1. Stratification of participants in the AI-assisted arm by educational level, including the number of board-certified pathologists

eTable 2. Univariable and multivariable cox regression analysis of tumor-infiltrating lymphocyte (TIL) scores in relation to disease-specific survival

eMethods. Supplementary methods

eAppendix 1. Instructions for automated TIL scoring

eAppendix 2. Instructions for manual TIL scoring

This supplementary material has been provided by the authors to give readers additional information about their work.

eFigure 1. Refinement of color normalization algorithm for H&E WSIs

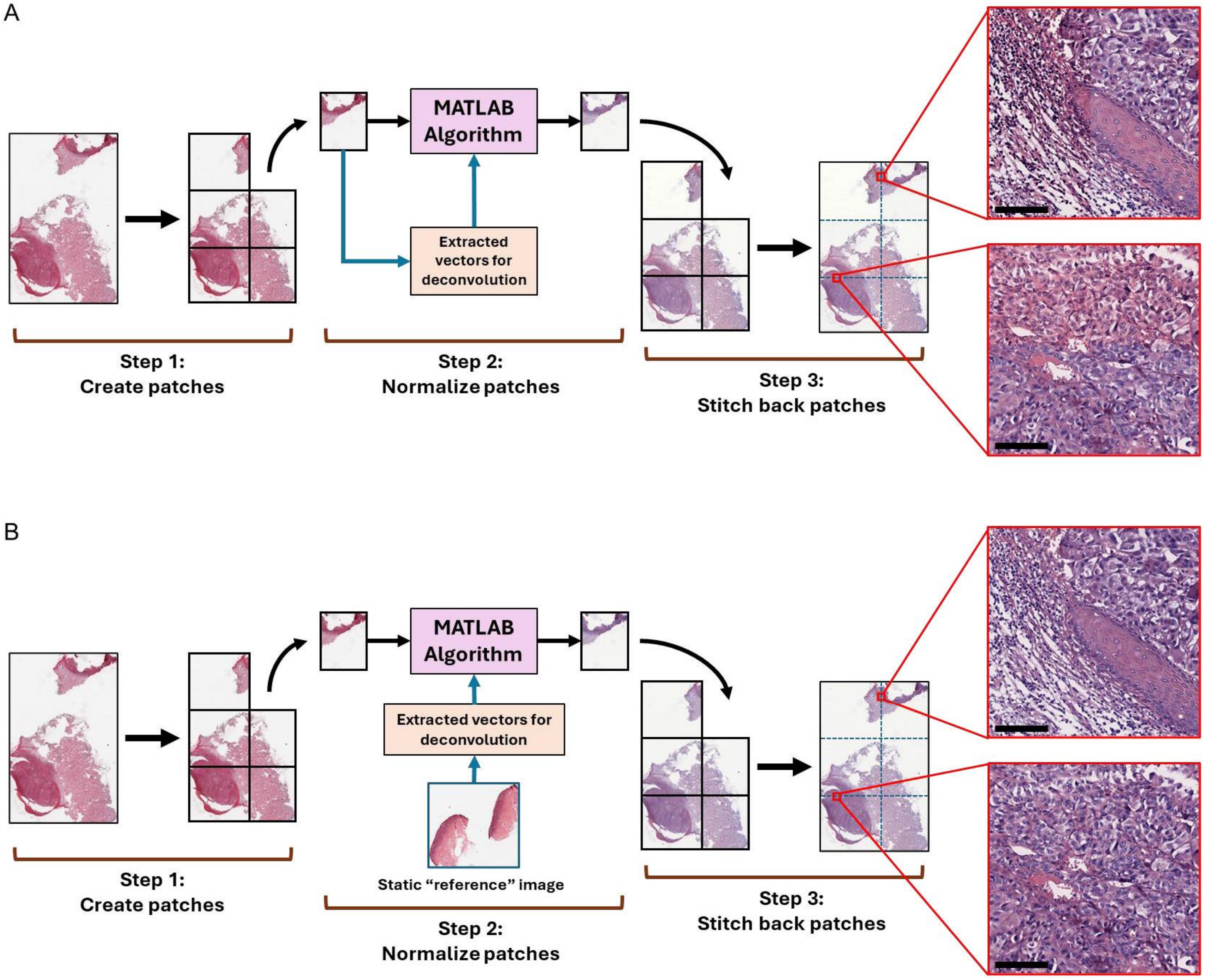

Supplementary Figure 1: Refinement of color normalization algorithm for H&E WSIs. (A) The initial algorithm displayed suboptimal results in color consistency. H&E images were divided into patches (to address computer memory limitations), inputted into the MATLAB script as implemented by Macenko et al. for normalization, and then reconstituted. The higher magnification regions of the sample image on the right reveal subtle coloring inconsistencies, which arose from the heterogeneity of the patches using their own extracted vectors during the deconvolution process in normalization. **(B)** The modified algorithm showed improved color consistency. H&E images were similarly split into patches, inputted into the MATLAB script, and reconstituted. The implementation of the MATLAB script was adjusted so that the extracted vectors from a representative, static "reference" image were used instead of each patch's individual extracted vectors for deconvolution. The higher magnification regions on the right exhibit color consistency across patches. Scale bars: 100 μm.

© 2025 Aung TN et al. *JAMA Network Open*.

eFigure 2. F1 score across different cell types

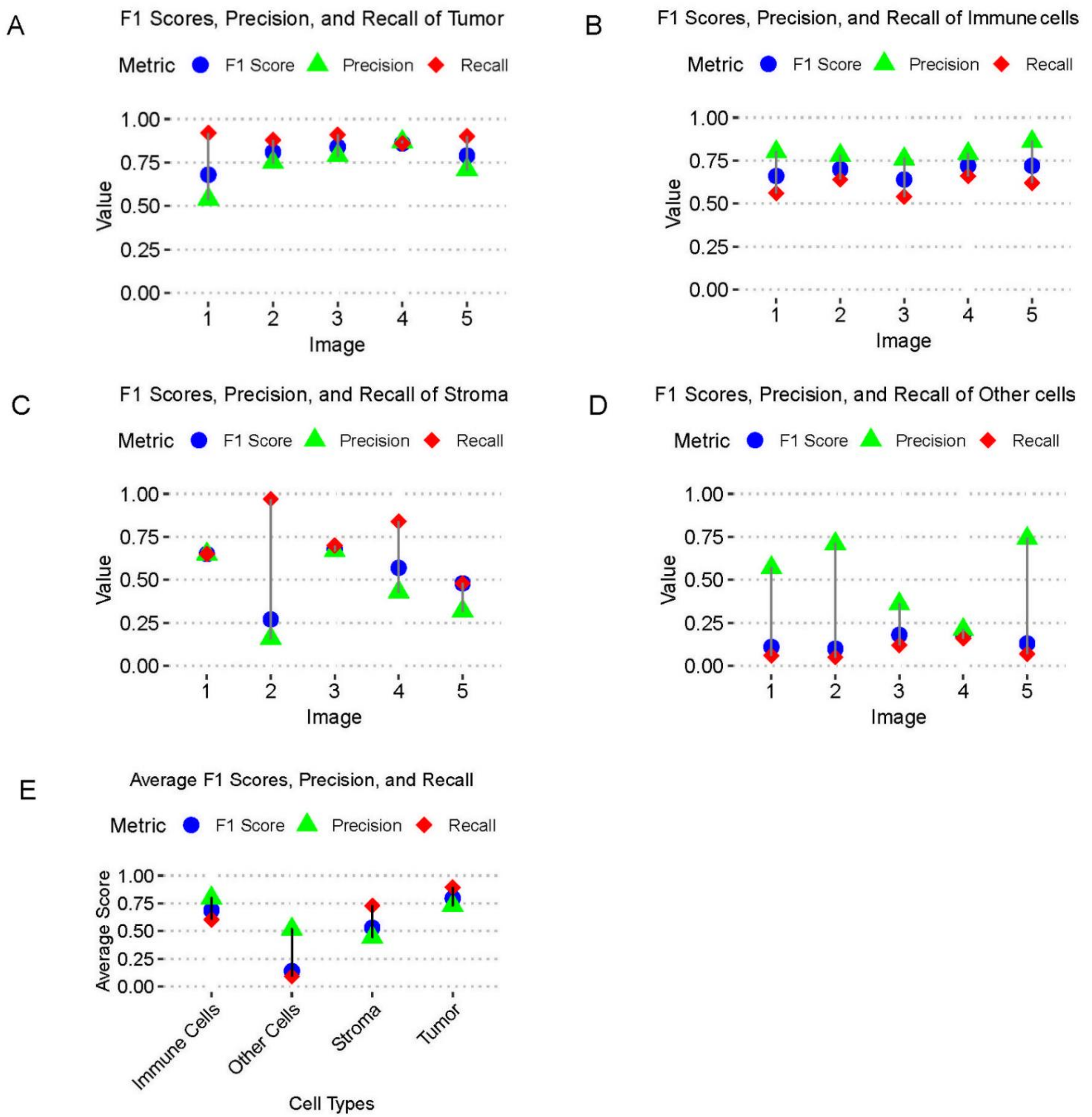

Supplementary Figure 2: F1 Score Across Different Cell Types. F1 scores, precision, and recall metrics for (A) tumor cells, (B) immune cells, (C) stromal cells, and (D) other cells are presented. The x-axis represents the image numbers (i.e., five images), and the y-axis denotes the corresponding F1 score values. (E) The average precision, recall, and F1 scores across all cell types are shown, providing an overall evaluation of model performance across the dataset.

© 2025 Aung TN et al. *JAMA Network Open*.

eFigure 3. Schematic representation of the five tumor-infiltrating lymphocyte (TIL) variables

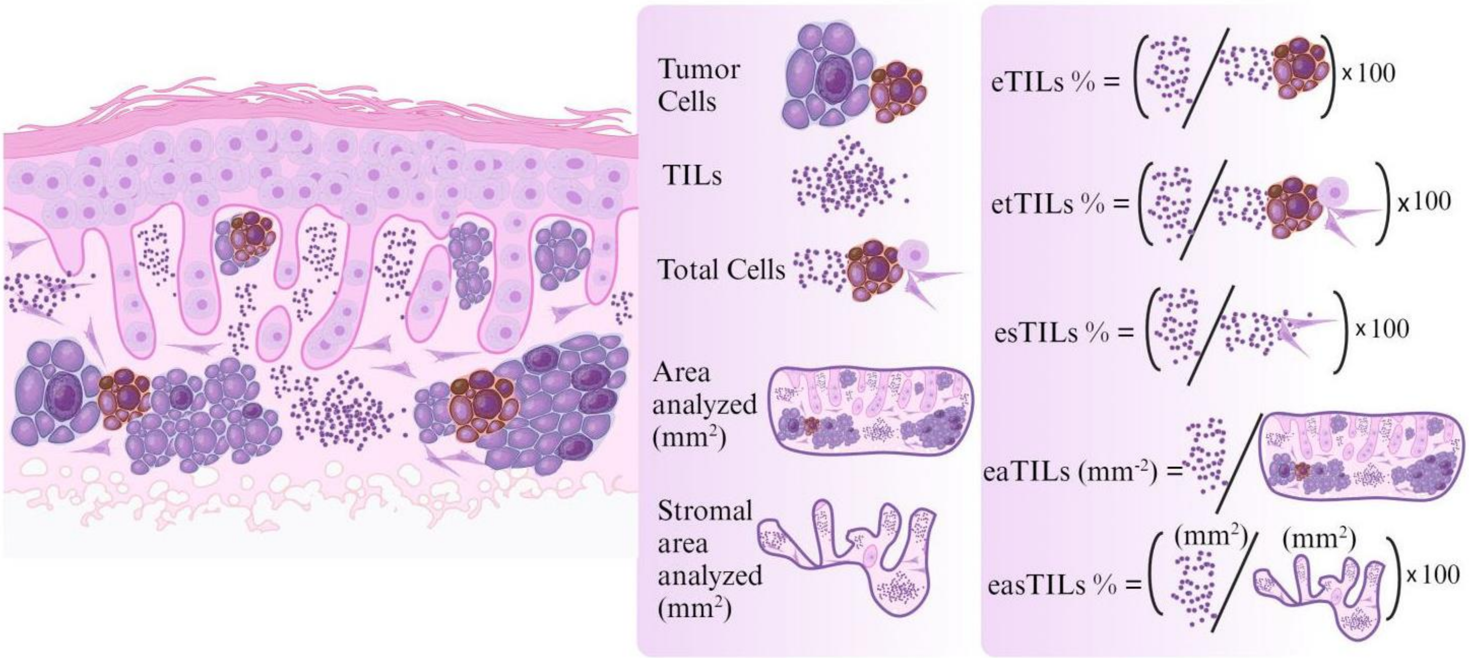

Supplementary Figure 3: Schematic representation of the five tumor-infiltrating lymphocyte (TIL) variables. Five machine-read TIL variables: eTILs%, etTILs%, esTILs%, eaTILs (mm^{-2}), and easTILs%, illustrating their respective calculations. This diagram provides a comprehensive visual breakdown of each variable, and it has been retouched from our previous publication (Aung et al., 2022) (created with biorender.com).

© 2025 Aung TN et al. *JAMA Network Open*.

eFigure 4. Interobserver variability among operators on the AI arm for additional TIL variables

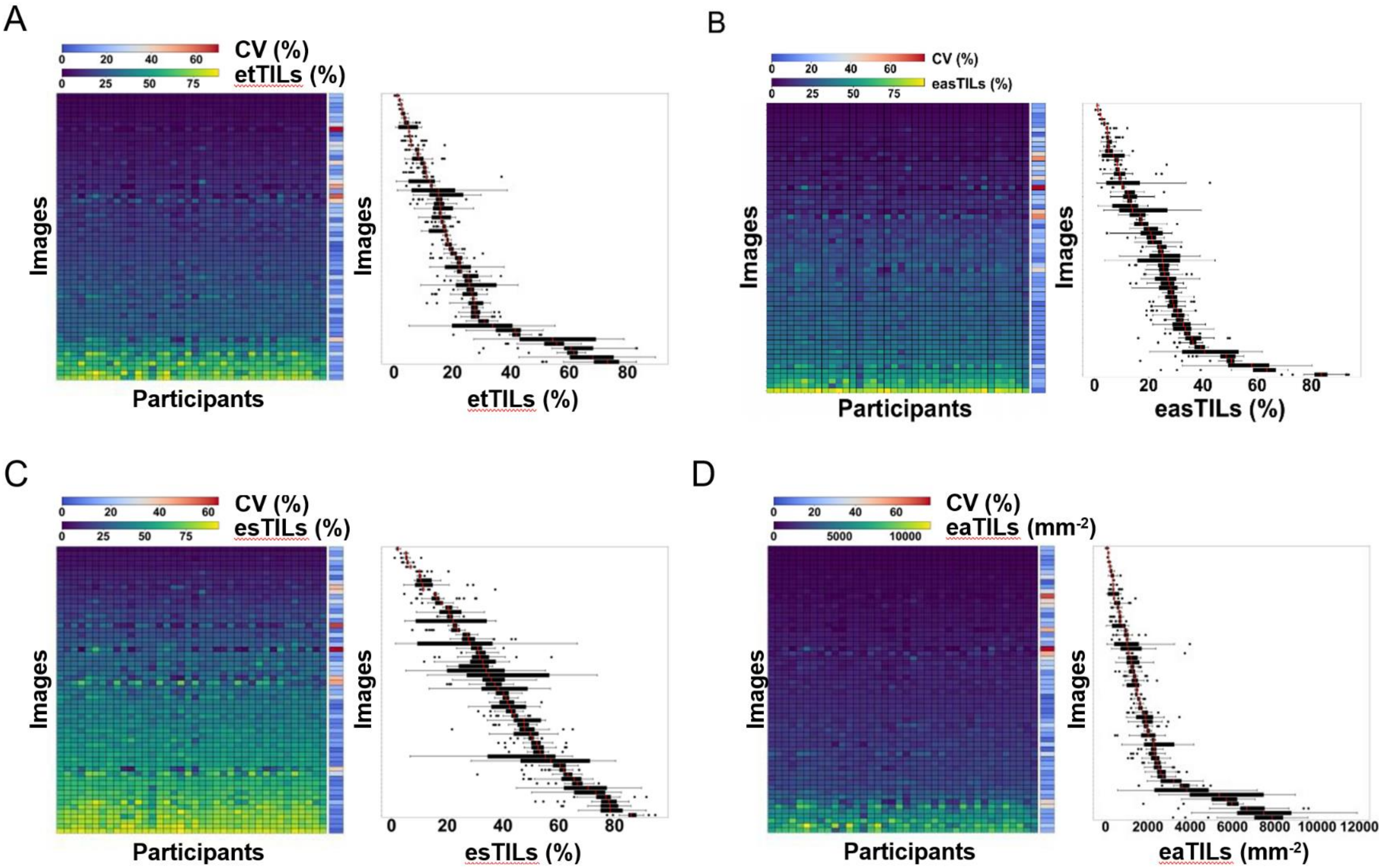

Supplementary Figure 4: Interobserver variability among operators on the AI arm for additional TIL variables. (A) etTILs, **(B)** easTILs, **(C)** esTILs and **D)** eaTILs are shown in heatmaps and corresponding boxplots. The vertical axes for the heatmap-boxplot sets are sorted by their respective median values.

© 2025 Aung TN et al. *JAMA Network Open*.

eFigure 5. Interobserver variability between operators on the AI arm, stratified by board-certified pathologists (n=11) and non-board-certified pathologists (n=27)

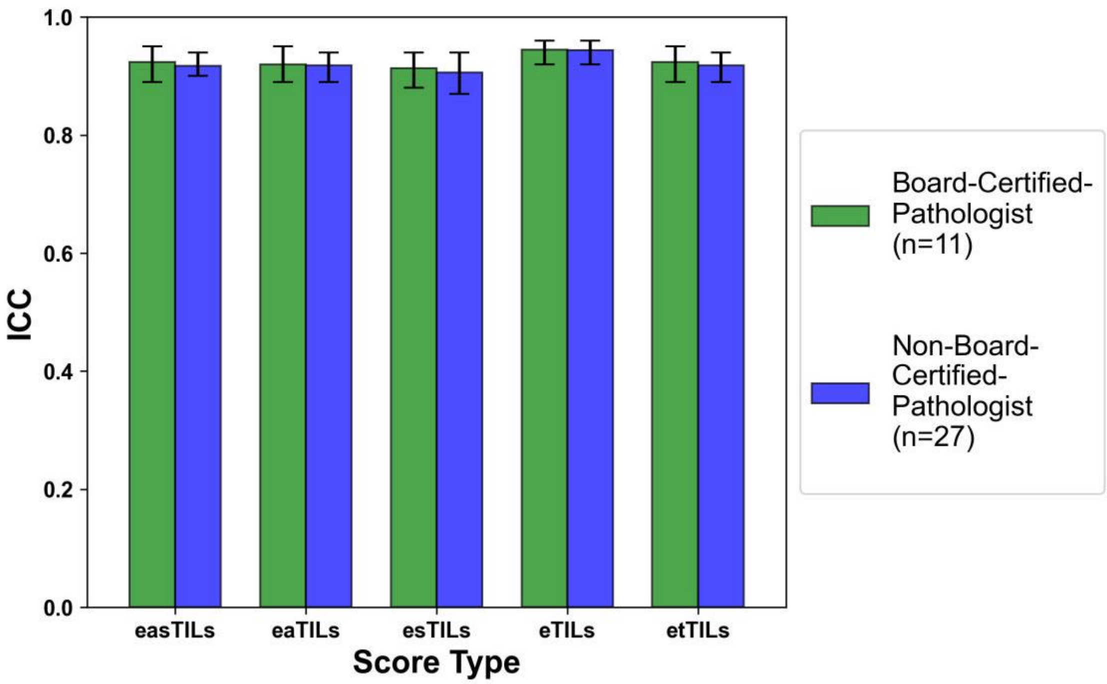

Supplementary Figure 5: Interobserver variability between operators on the AI arm, stratified by board-certified pathologists (n=11) and non-board-certified pathologists (n=27). Concordance statistics for TIL variables from the AI arm are shown for board-certified pathologists (easTILs ICC = 0.924, 95% CI: 0.89-0.95; eaTILs ICC = 0.92, 95% CI: 0.89-0.95; esTILs ICC = 0.913, 95% CI: 0.88-0.94; eTILs ICC = 0.945, 95% CI: 0.92-0.96; etTILs ICC = 0.924, 95% CI:0.89-0.95) and non-board-certified pathologists (easTILs ICC = 0.917, 95% CI: 0.90-0.94; eaTILs ICC = 0.918, 95% CI: 0.89-0.94; esTILs ICC = 0.906, 95% CI: 0.87-0.94; eTILs ICC = 0.944, 95% CI: 0.92-0.96; etTILs ICC = 0.918, 95% CI: 0.89-0.94), highlighting comparable variability. Error bars in the figure represent 95% confidence intervals.

© 2025 Aung TN et al. *JAMA Network Open*.

eFigure 6. Comparative survival analysis based on TIL scoring methods

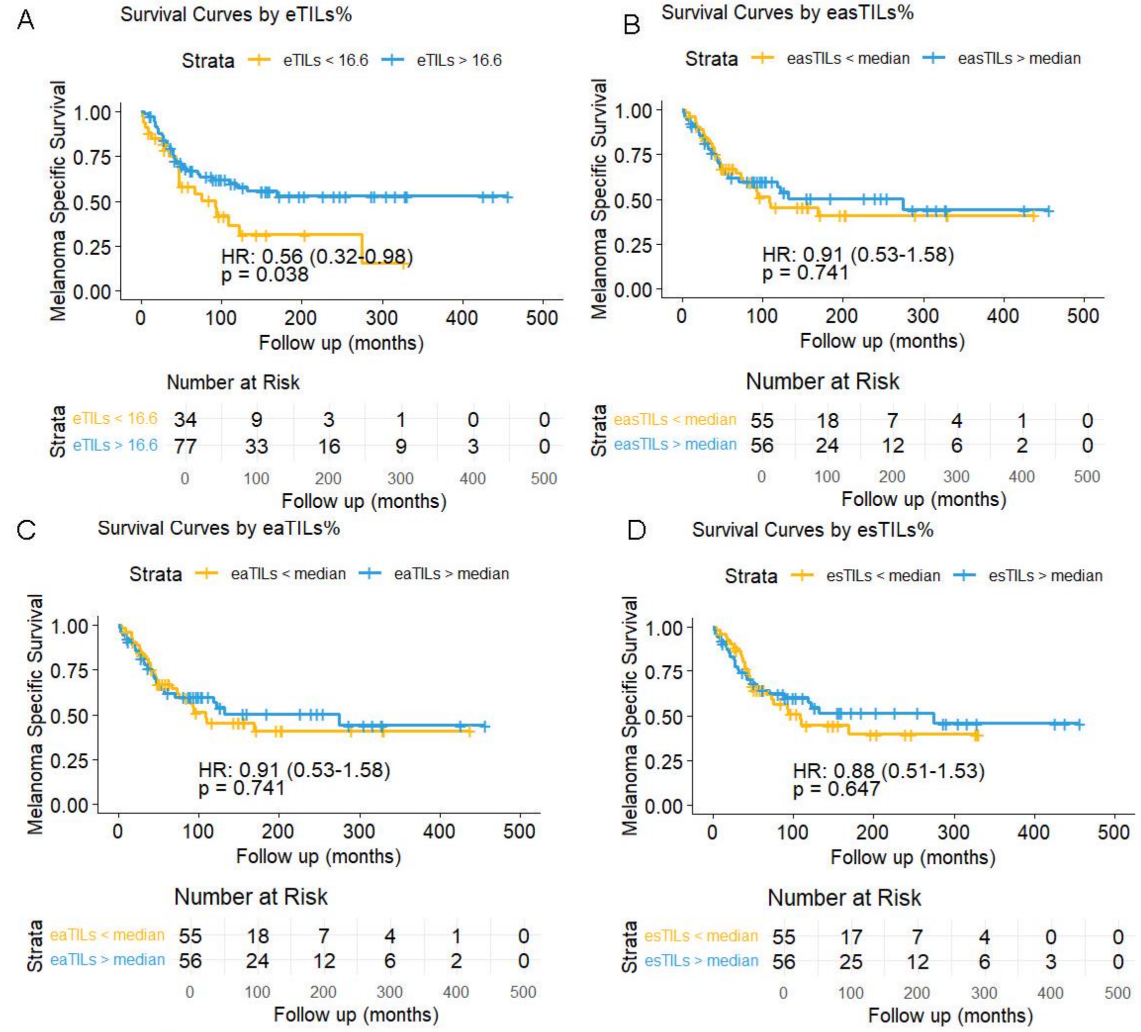

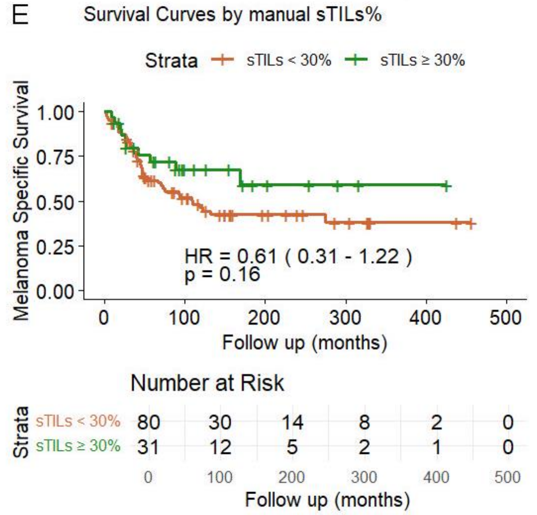

Supplementary Figure 6: Comparative Survival Analysis Based on TIL Scoring Methods. Kaplan-Meier survival curve stratified by **(A)** eTILs using the published cutpoint 16.6%, (**B**) esTILs% using the median cutpoint **(C)** eaTILs%, divided by the median cutpoint **(D)** easTILs%, divided by the median cutpoint and **(E)** manual sTILs%, split by 30% are shown.

© 2025 Aung TN et al. *JAMA Network Open*.

eFigure 7. Example of an H&E image given to the participants, revealing differences in AI-based TILs scores across 6 selected participants (initials shown) who enrolled in the AI arm

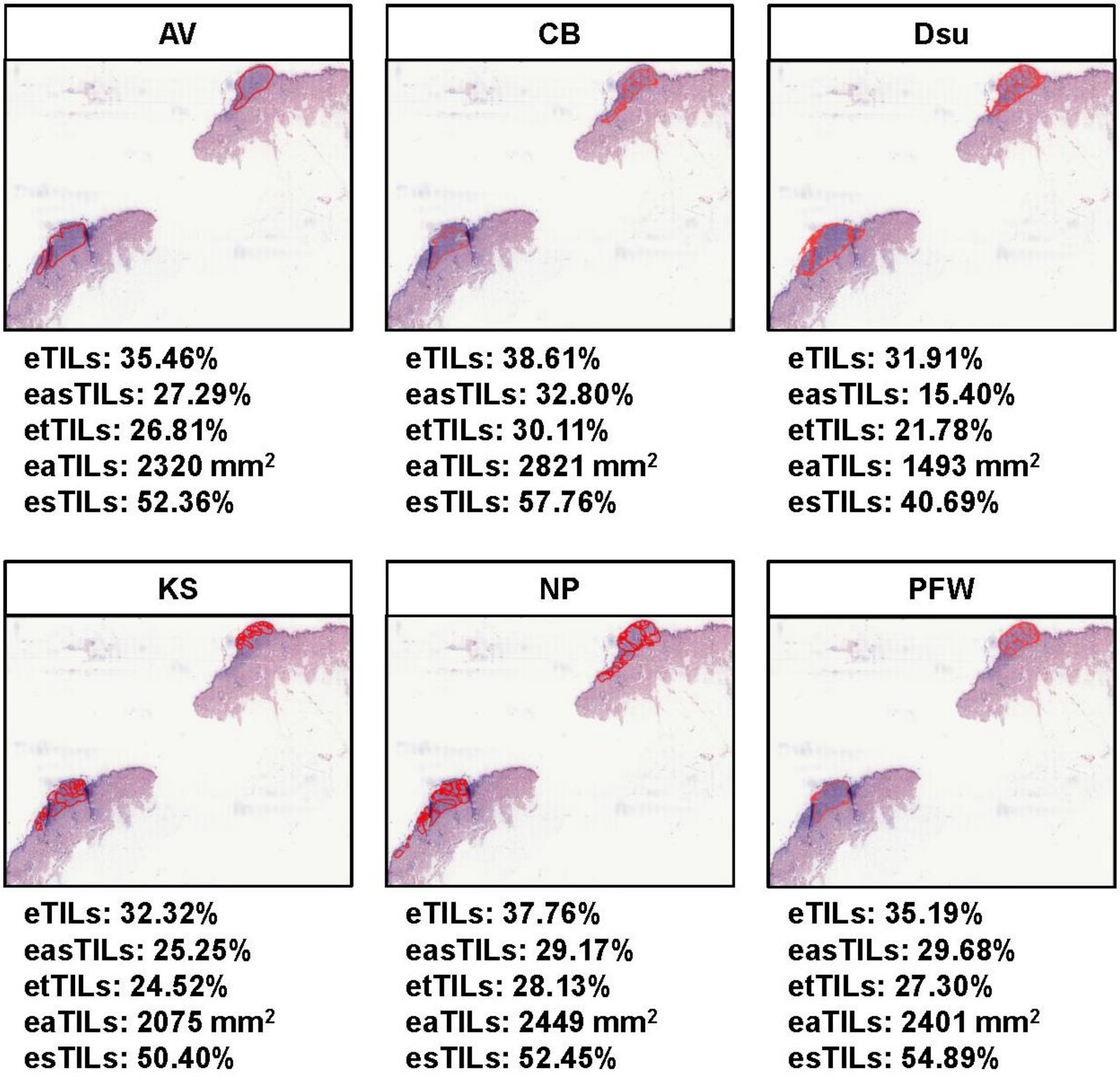

Supplementary Figure 7: Example of an H&E image (as with Supplementary Figures 6 and 7) given to the participants, revealing differences in AI-based TILs scores across 6 select participants (initials shown) who enrolled in the AI arm. The variability is attributed to differences in ROI selection among the participants, as can be seen from the overlaid annotations.

© 2025 Aung TN et al. *JAMA Network Open*.

eFigure 8. A second example of an H&E image given to the participants, revealing differences in AI-based TILs scores across 6 selected participants (initials shown) who enrolled in the AI arm

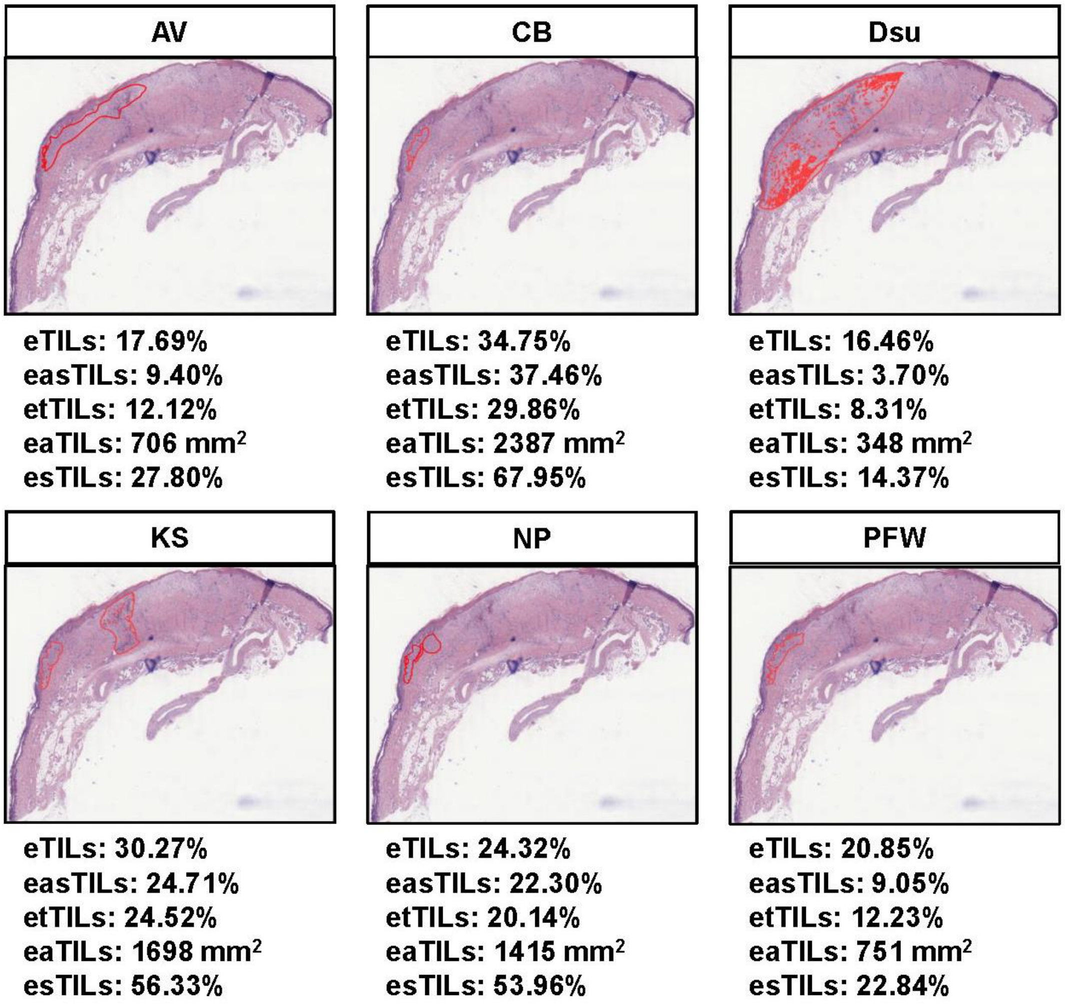

Supplementary Figure 8: Example of an H&E image (as with Supplementary Figures 5 and 7) given to the participants, revealing differences in AI-based TILs scores across 6 select participants (initials shown) who enrolled in the AI arm. The variability is attributed to differences in ROI selection among the participants, as can be seen from the overlaid annotations.

© 2025 Aung TN et al. *JAMA Network Open*.

eFigure 9. A third example of an H&E image given to the participants, revealing differences in AI-based TILs scores across 6 selected participants (initials shown) who enrolled in the AI arm

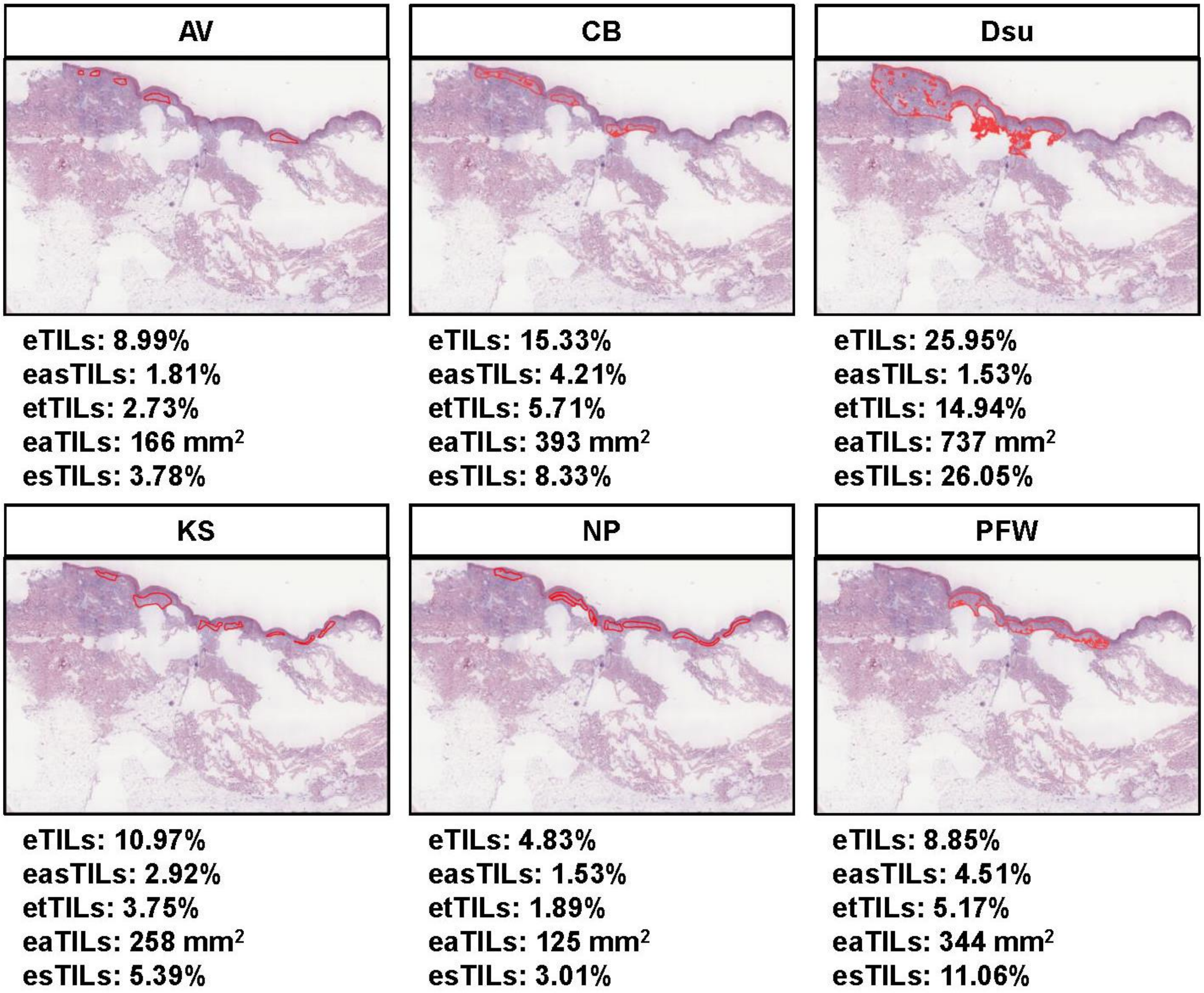

Supplementary Figure 9: Example of an H&E image (as with Supplementary Figures 5 and 6) given to the participants, revealing differences in AI-based TILs scores across 6 select participants (initials shown) who enrolled in the AI arm. The variability is attributed to differences in ROI selection among the participants, as can be seen from the overlaid annotations.

© 2025 Aung TN et al. *JAMA Network Open*.

eTable 1. Stratification of participants in the AI-assisted arm by educational level, including the number of board-certified pathologists

Supplementary Table 1: Stratification of Participants in the AI-Assisted Arm by Educational Level, Including the Number of Board-Certified Pathologists. Stratification of the participants from the AI-assisted arm who remained active in the study (n=38) by highest educational level (BSc, MSc, MD only, PhD only, and MD-PhD). The number of board-certified pathologists from the MD and MD-PhD groups is also indicated.

Educational Level	Number of Participants	Board-Certified Pathologists
BSc	4	N/A
MSc	3	N/A
MD only	12	5
PhD only	7	N/A
MD-PhD	12	6
Total	38	11

eTable 2. Univariable and multivariable cox regression analysis of tumor-infiltrating lymphocyte (TIL) scores in relation to disease-specific survival

Supplementary Table 2: Univariable and Multivariable Cox Regression Analysis of Tumor-Infiltrating Lymphocyte (TIL) Scores in Relation to Disease-Specific Survival. Univariable and multivariable Cox regression models assessing the association between various TIL score cutoffs (eTILs ≥16.6%, etTILs ≥median, eaTILs ≥median, esTILs ≥median, and sTILs >30%) and disease-specific survival. In the multivariable models, each TIL score analyzed by its cutoff is adjusted for sex, age, and tumor stage (I–IV). Hazard ratios (HR) are reported with 95% confidence intervals (CI).

TIL Score Type	Variable	Univariable HR (95% CI)	Univariable p-value	Multivariable HR (95% CI)	Multivariable p-value
eTILs (16.6% cutoff)	eTILs: ≥16.6%	0.56 (0.32–0.98)	0.041 *	0.66 (0.35–1.24)	0.194
	Sex (Male)	-	-	1.51 (0.82–2.79)	0.188
	Age	-	-	1.02 (0.99–1.04)	0.166
	Stage II	-	-	1.61 (0.38–6.90)	0.519
	Stage III	-	-	4.60 (2.21–9.60)	<0.001 ***
	Stage IV	-	-	4.98 (1.08–22.99)	0.040 *
etTILs (median cutoff)	etTILs: ≥median	0.47 (0.27–0.83)	0.008 **	0.53 (0.29–0.97)	0.038 *
	Sex (Male)	-	-	1.52 (0.82–2.81)	0.184
	Age	-	-	1.02 (0.99–1.04)	0.144
	Stage II	-	-	1.50 (0.36–6.26)	0.582
	Stage III	-	-	4.45 (2.12–9.32)	<0.001 ***
	Stage IV	-	-	4.99 (1.10–22.75)	0.038 *
eaTILs (median cutoff)	eaTILs: ≥median	0.91 (0.53–1.58)	0.741	0.89 (0.48–1.66)	0.717
	Sex (Male)	-	-	1.49 (0.80–2.75)	0.208
	Age	-	-	1.01 (0.99–1.04)	0.255
	Stage II	-	-	1.40 (0.33–5.92)	0.646
	Stage III	-	-	4.71 (2.20–10.09)	<0.001 ***
	Stage IV	-	-	5.82 (1.27–26.70)	0.023 *
esTILs (median cutoff)	esTILs: ≥median	0.88 (0.51–1.53)	0.647	1.01 (0.55–1.86)	0.973
	Sex (Male)	-	-	1.50 (0.81–2.77)	0.192
	Age	-	-	1.02 (0.99–1.04)	0.229
	Stage II	-	-	1.42 (0.34–5.99)	0.632
	Stage III	-	-	4.57 (2.18–9.56)	<0.001 ***
	Stage IV	-	-	6.16 (1.34–28.32)	0.020 *
sTILs (30% cutoff)	sTILs: ≥30%	0.61 (0.31–1.22)	0.166	0.64 (0.28–1.46)	0.291
	Sex (Male)	-	-	1.55 (0.84–2.87)	0.164
	Age	-	-	1.01 (0.99–1.04)	0.273
	Stage II	-	-	1.77 (0.40–7.87)	0.455
	Stage III	-	-	4.11 (1.93–8.76)	<0.001 ***
	Stage IV	-	-	5.72 (1.27–25.73)	0.023 *

© 2025 Aung TN et al. *JAMA Network Open*.

eMethods. Supplementary methods

Calculation methods for machine-derived TIL variables

For a given region of interest (ROI):

1. $eTILs\% = \left(\frac{tumor\ cell\ count}{tumor\ cell\ count + immune\ cell\ count}\right) \times 100$, representing the proportion of TILs over tumor cells.
2. $etTILs\% = \left(\frac{tumor\ cell\ count}{total\ cell\ count}\right) \times 100$, representing the proportion of TILs over all cells.
3. $esTILs\% = \left(\frac{tumor\ cell\ count}{total\ cell\ count - tumor\ cell\ count}\right) \times 100$, representing the proportion of TILs over non-tumor (i.e., stromal) cells.
4. $eaTILs\ (mm^{-2}) = \frac{immune\ cell\ count}{ROI\ area}$, representing the density of TILs over the ROI.
5. $easTILs\% = \left(\frac{immune\ cell\ area}{ROI\ area - tumor\ cell\ area}\right) \times 100$, representing the density of TILs over non-tumor (i.e., stromal) area.

HE Color normalization of WTS images

While traditional inspection of H&E slides by pathologists does not encounter issues with variations in stain color intensities for patient diagnosis, these variations present significant challenges for ML algorithms, particularly in cell segmentation. To address this, we employed a method developed by Macenko et al. to remove confounding factors affecting stain intensity, providing a robust basis for direct analytical analysis and consistent color normalization across large datasets. This method was integrated into our workflow using MATLAB to achieve reliable results. However, color normalization was challenging without patching large WSIs. Normalizing patches individually led to color inconsistencies within a single WTS (Supplementary Figure 1A). To resolve this, we modified the workflow with the use of a surrogate “reference” image, from which the stain vectors were extracted from the filtered optical density (encoded as “ODhat” in the MATLAB source code). These vectors were then uniformly applied to all patches in the deconvolution process. This ensured that all patches were consistently normalized to the same color space, removing the risk of heterogeneity when stitching the patches back together (Supplementary Figure 1B).

Development of ANNMAR_24 algorithm

We annotated a total of 7,938 cells from the whole training cohort (primary cutaneus on stain vector normalized WTS images using QuPath 0.4.3 [39]. The annotated cells were categorized into four distinct types: tumor cells, stromal cells, immune cells, and other cells. The “other cells” category included melanocytic cells covered by melanin, dead cells, red blood cells, and neutrophils with

© 2025 Aung TN et al. *JAMA Network Open*.

polymorphonuclear leukocytes. These annotations formed the basis for training the algorithm. Using the annotated and color-normalized images, we developed an algorithm using the Artificial Neural Network Multilayer Perceptron (ANN_MLP) model in QuPath to identify and classify the four cell types mentioned above. The annotated cells were Tumor, Stroma, Immune cells and Other. Using QuPath's annotation tools, we manually identified and marked the different cell types. Tumor cells were identified based on their morphological features, such as irregular nuclei and high nucleus-to-cytoplasm ratio. Stromal cells, such as fibroblasts, were distinguished by their elongated shape and presence in the tumor microenvironment. Immune cells (specifically lymphocytes) were identified by their smaller size and dense, round nuclei. The "other cells" category comprises melanocytic cells, dead cells, red blood cells, neutrophils with polymorphonuclear leukocytes. The ground truth annotations were established based on cell annotations by a board-certified pathologist, which served as the reference standard against which the algorithm's performance was measured. These detailed annotations were essential in forming the training dataset for our ML algorithm. Using the annotated and color-normalized images, we developed an algorithm with the ANN_MLP, called ANNMAR_24, configured under QuPath's default settings for the input layer, hidden layers, and output layer.

Performance (F1-Score) evaluation of the ANNMAR_24 algorithm

Our cell detection enhancer extension within QuPath facilitated the annotation process, enabling precise annotation-creation from individual cells and enhanced drag selection for cells. This allowed us to annotate cells individually rather than in groups, overcoming the limitations of varying watershed cell segmentation patterns. We then trained our ANN_MLP classifier, characterized by its multiple interconnected artificial neural network layers. The performance of our TILs scoring algorithm, ANNMAR_24, was evaluated using the F1 score, which balances precision and recall, across four different categories of cells ("Tumor," "Stroma," "Immune cells," and "Other") from five tested images. The F1 score, calculated as the harmonic mean of precision and recall, provided a balanced measure of the algorithm's effectiveness in accurately identifying and classifying TILs. A confusion matrix was constructed where each entry (i, j) represents the count of cells annotated as $i$in "cells_1" and detected as class j in "cells_2". For each class pair (i, j), we identified the indices of cells in both datasets where the annotations and detections classes matched and populated the confusion matrix with these counts. The row sums (actual counts) and column sums (predicted counts) of the confusion matrix were computed. Using these, we calculated True Positives (TP), False Negatives (FN), and False Positives (FP) for each class. Precision for each class was calculated as $TP/(TP + FP)$, recall as $TP/(TP + FN)$, and the F1 score as $2 \times TP/(2 \times TP + FP + FN)$.

© 2025 Aung TN et al. *JAMA Network Open*.

eAppendix 1

Instructions for Automated TIL Scoring

TILs Melanoma Study

© 2025 Aung TN et al. *JAMA Network Open*.

Step 1: Download the H&E Images

1. Click here to find the images.
    - You may be prompted to enter your email for a verification code.

© 2025 Aung TN et al. *JAMA Network Open.*

Step 1: Download the H&E Images

2. Select all the images and click “Download”

- This will be a heavy download. Make sure your computer or drive has at least 100 GB.

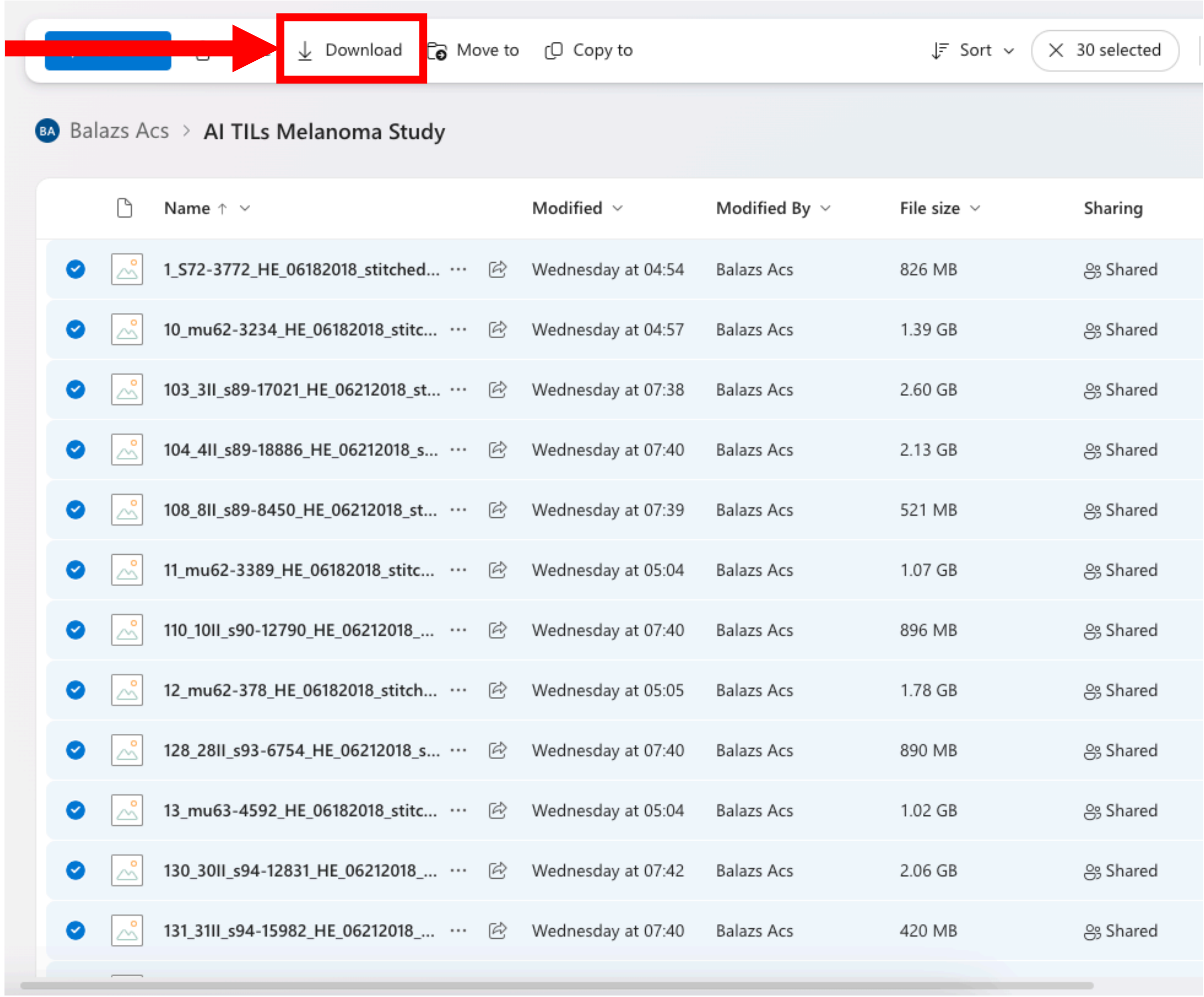

© 2025 Aung TN et al. *JAMA Network Open*.

Step 1: Download the H&E Images

3. Once downloaded (and extracted as needed), keep the images in a safe location in your computer.

- We highly recommend putting them in a location where they won't be changed or removed. This will ensure fidelity of subsequent steps.

© 2025 Aung TN et al. *JAMA Network Open*.

Step 2: Install QuPath

1. Download the latest version of QuPath here, according to your system (macOS, Windows, Linux).

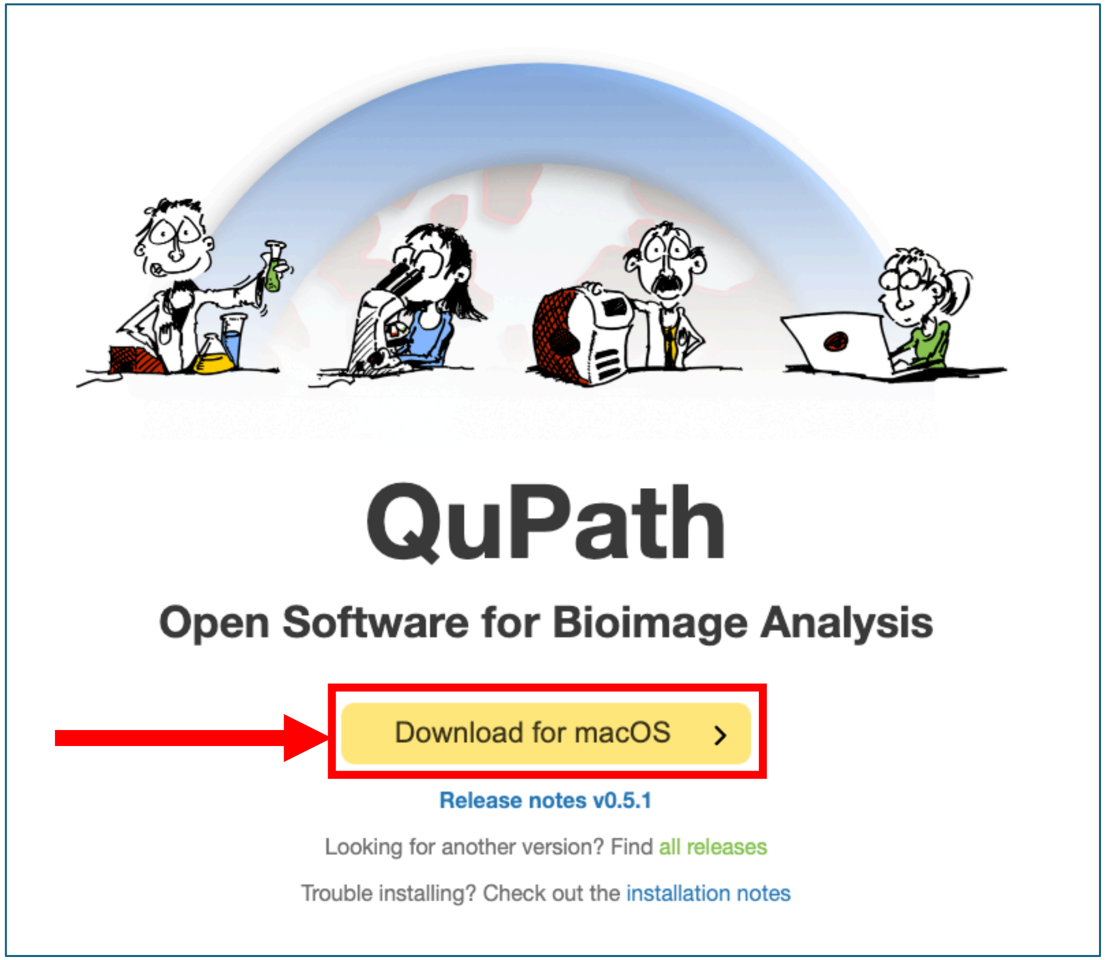

© 2025 Aung TN et al. *JAMA Network Open.*

Step 2: Install QuPath

2. Open the downloaded package and follow the installation instructions (may vary depending on system).

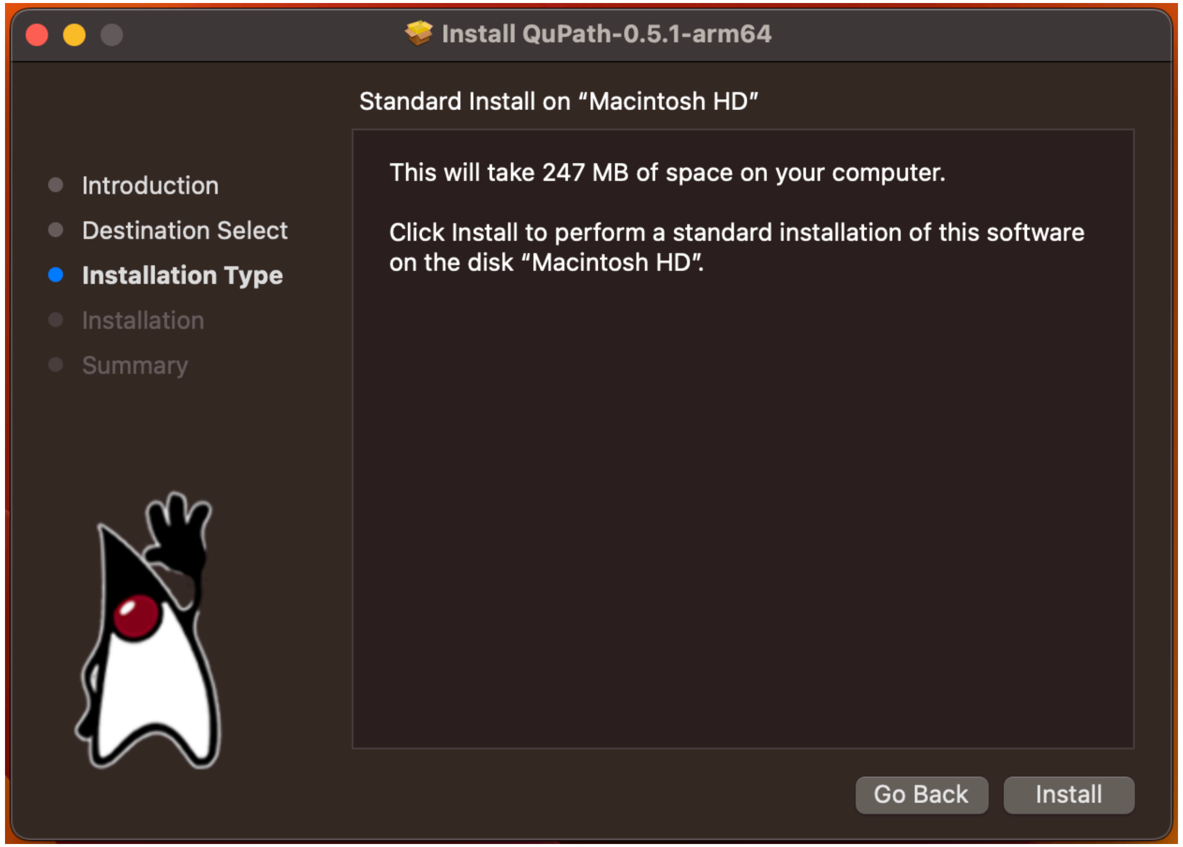

© 2025 Aung TN et al. *JAMA Network Open.*

Step 3: Create a QuPath Project

1. Open QuPath

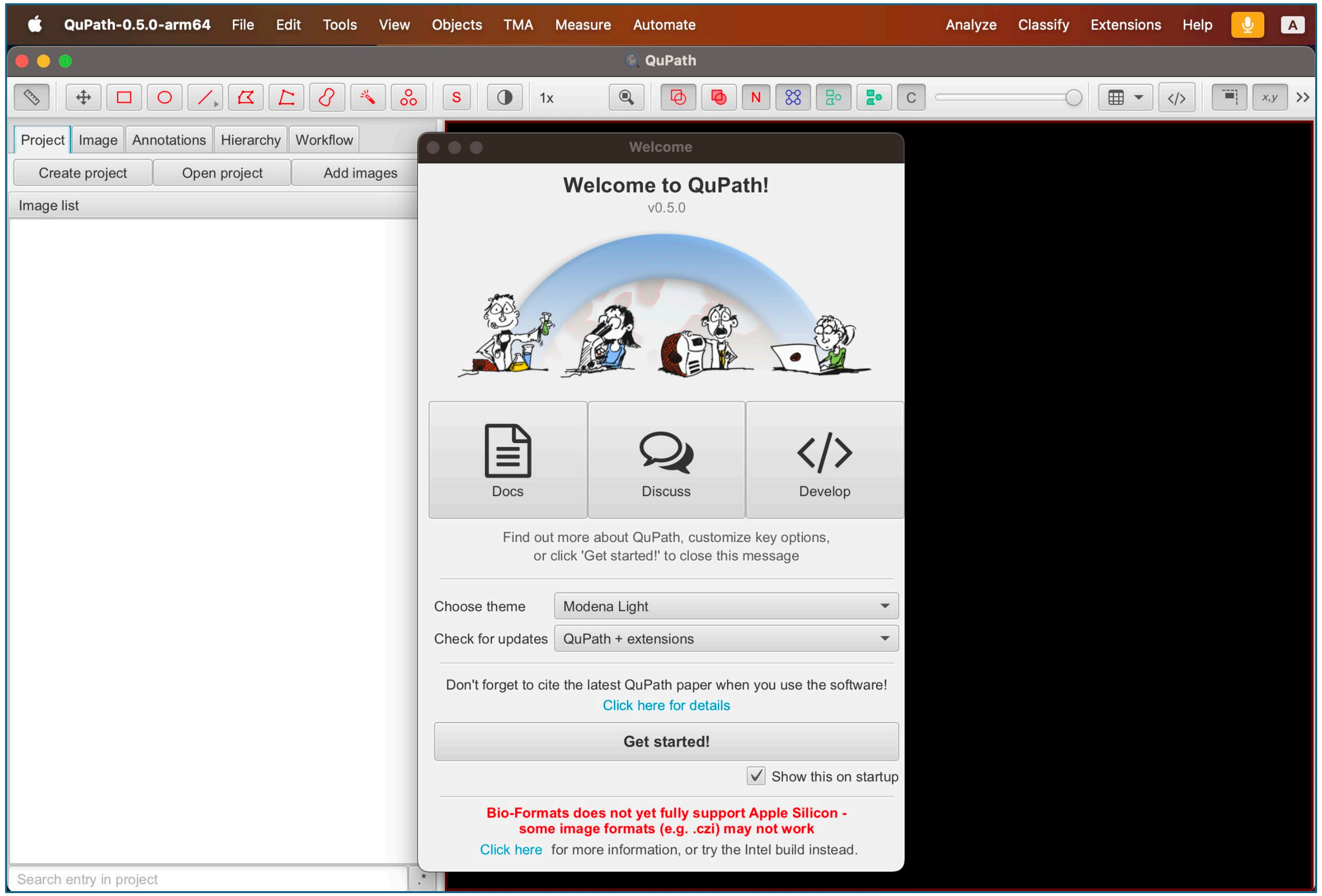

© 2025 Aung TN et al. *JAMA Network Open.*

Step 3: Create a QuPath Project

2. Create a new project by clicking on ”File” → “Project…” → “Create Project”

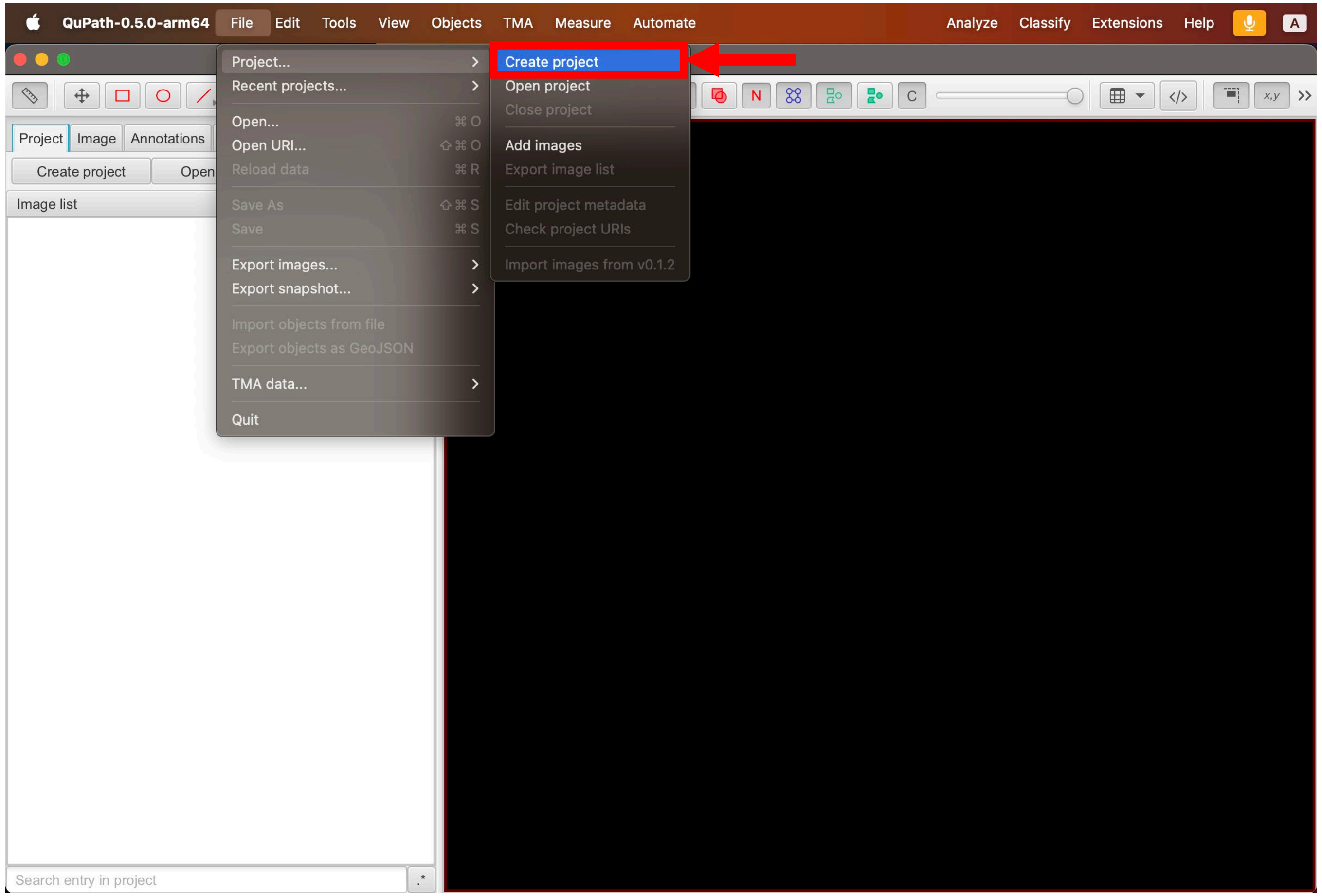

© 2025 Aung TN et al. *JAMA Network Open*.

Step 3: Create a QuPath Project

3. In the pop-up window, select an empty folder (create one if you haven't already) to finish creating your project.

(Note: Like for the images, it's best to have your project folder in a safe location.)

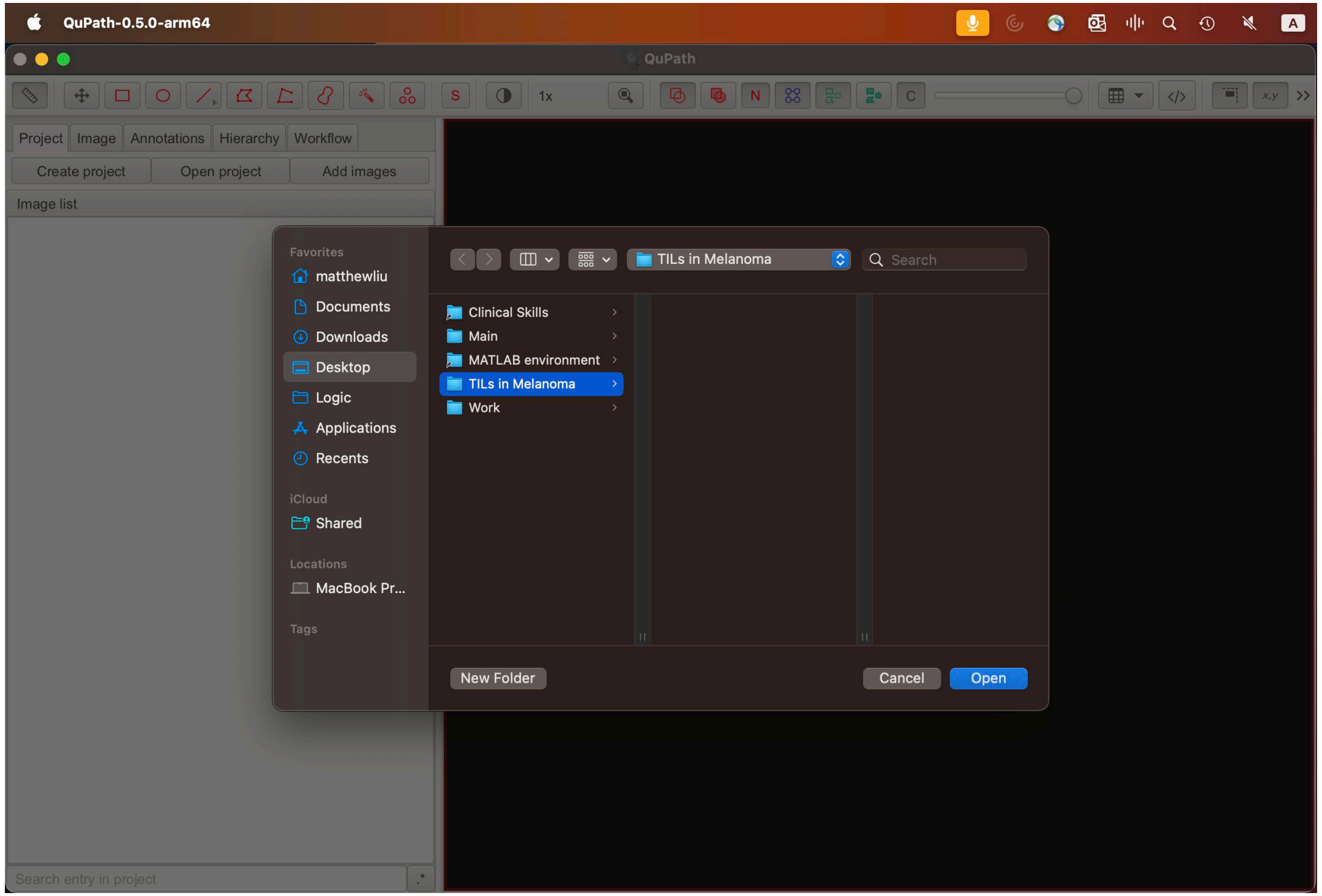

© 2025 Aung TN et al. *JAMA Network Open.*

Step 4: Load the images

1. Take the images you downloaded and drag them onto your QuPath project.

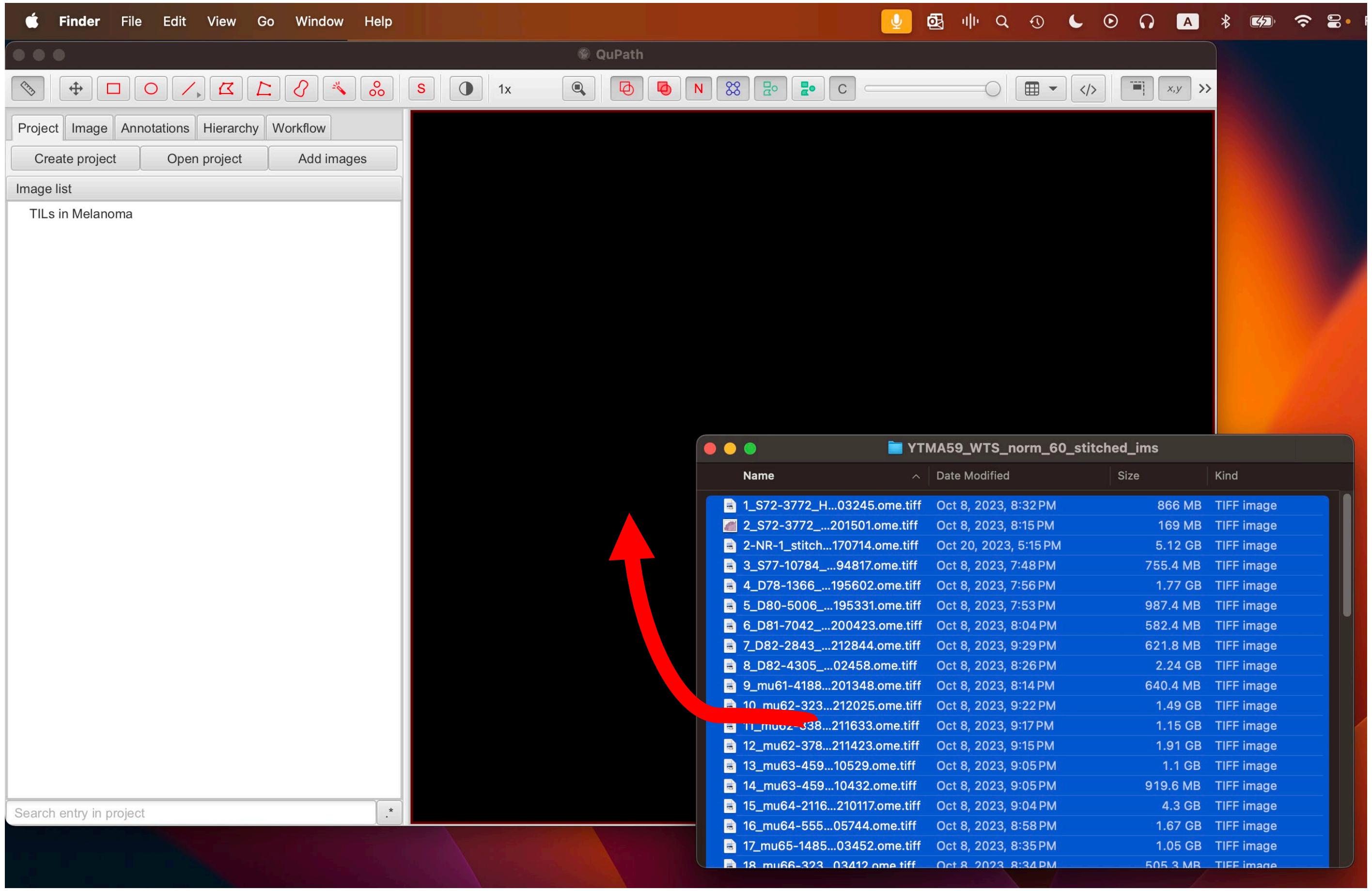

© 2025 Aung TN et al. *JAMA Network Open*.

Step 4: Load the images

2. In the pop-up prompt, simply set the image type to “Brightfield (H&E)” and then click “Import”

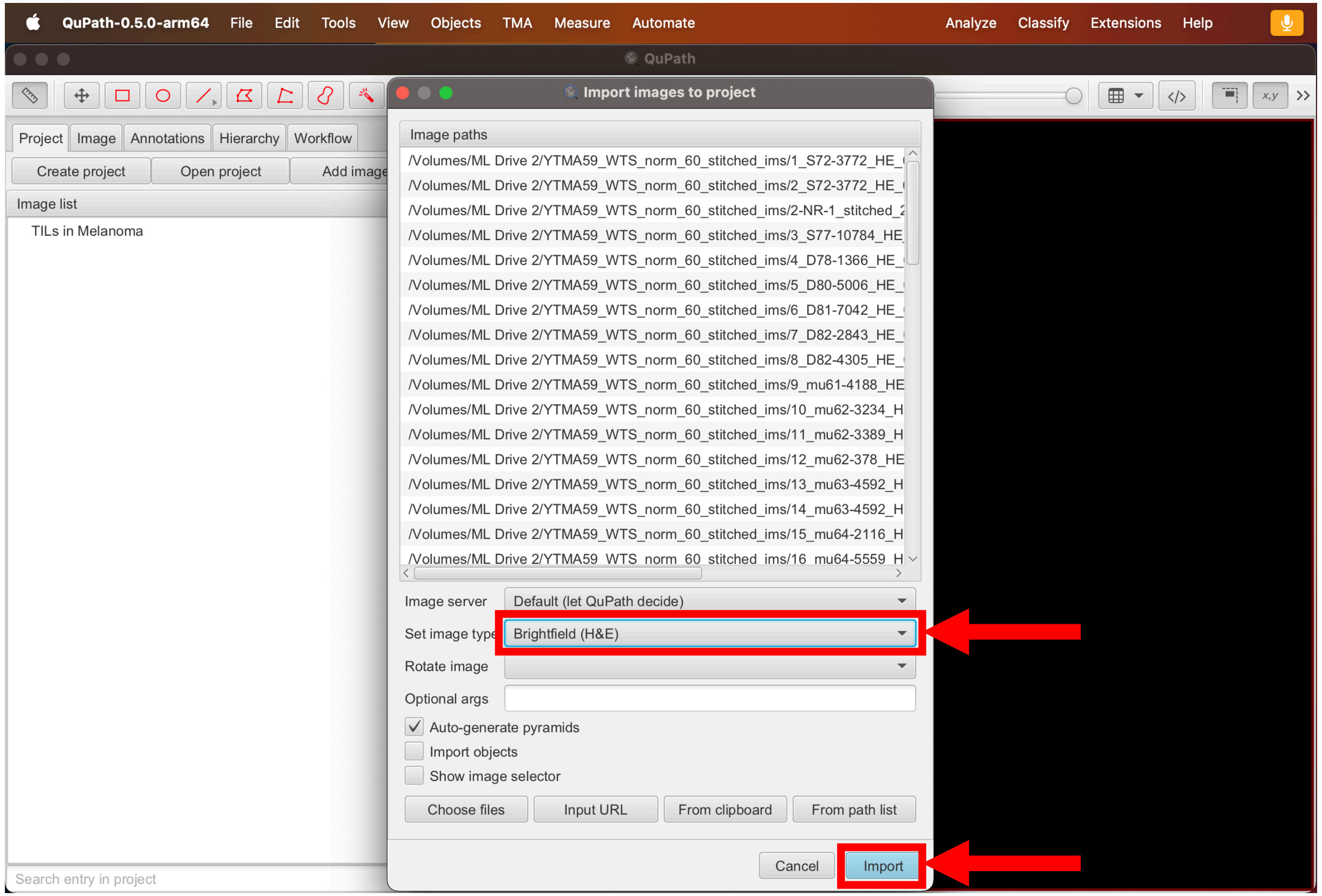

© 2025 Aung TN et al. *JAMA Network Open.*

Step 5: Annotate the Images

1. Back on QuPath, select to open images.

- You can navigate by double-clicking on the images on the side pane.

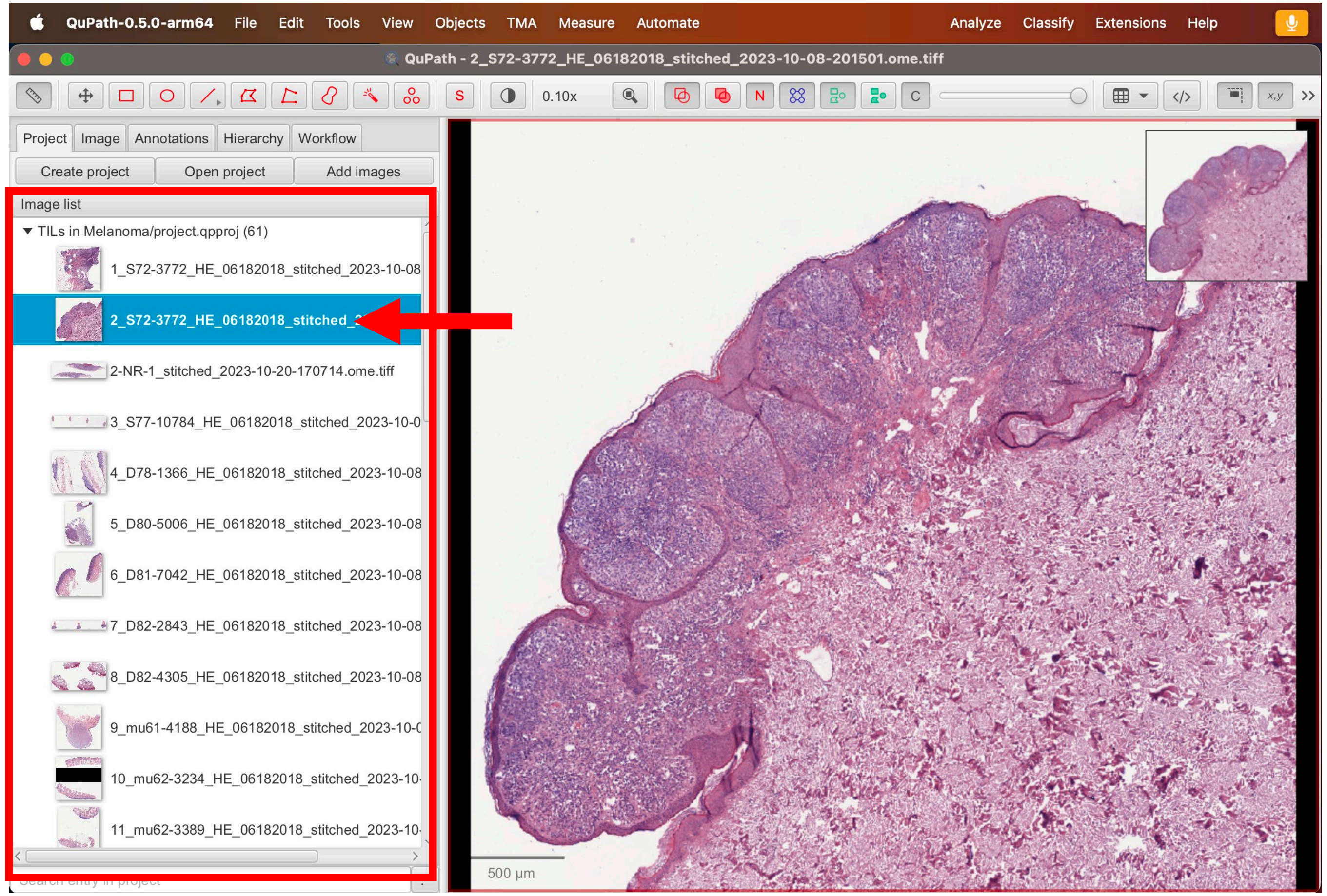

© 2025 Aung TN et al. *JAMA Network Open.*

Step 5: Annotate the Images

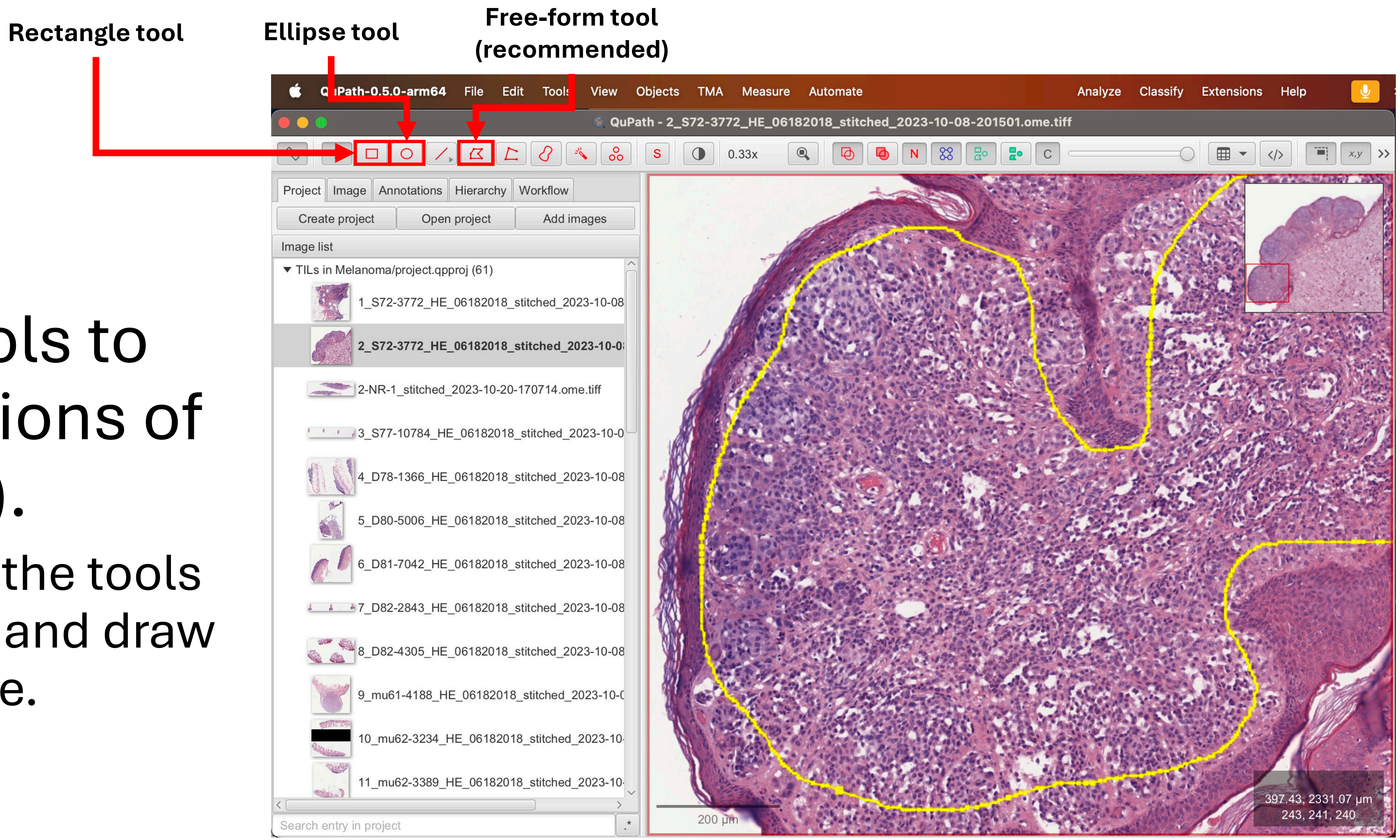

2. Use the annotation tools to make your regions of interest (ROIs).

- Select one of the tools shown above and draw over the image.

© 2025 Aung TN et al. *JAMA Network Open.*

Step 5: Annotate the Images

Tip 1: *It's ok to make multiple annotations for a given image. (The script will ultimately merge them to make one consolidated ROI).*

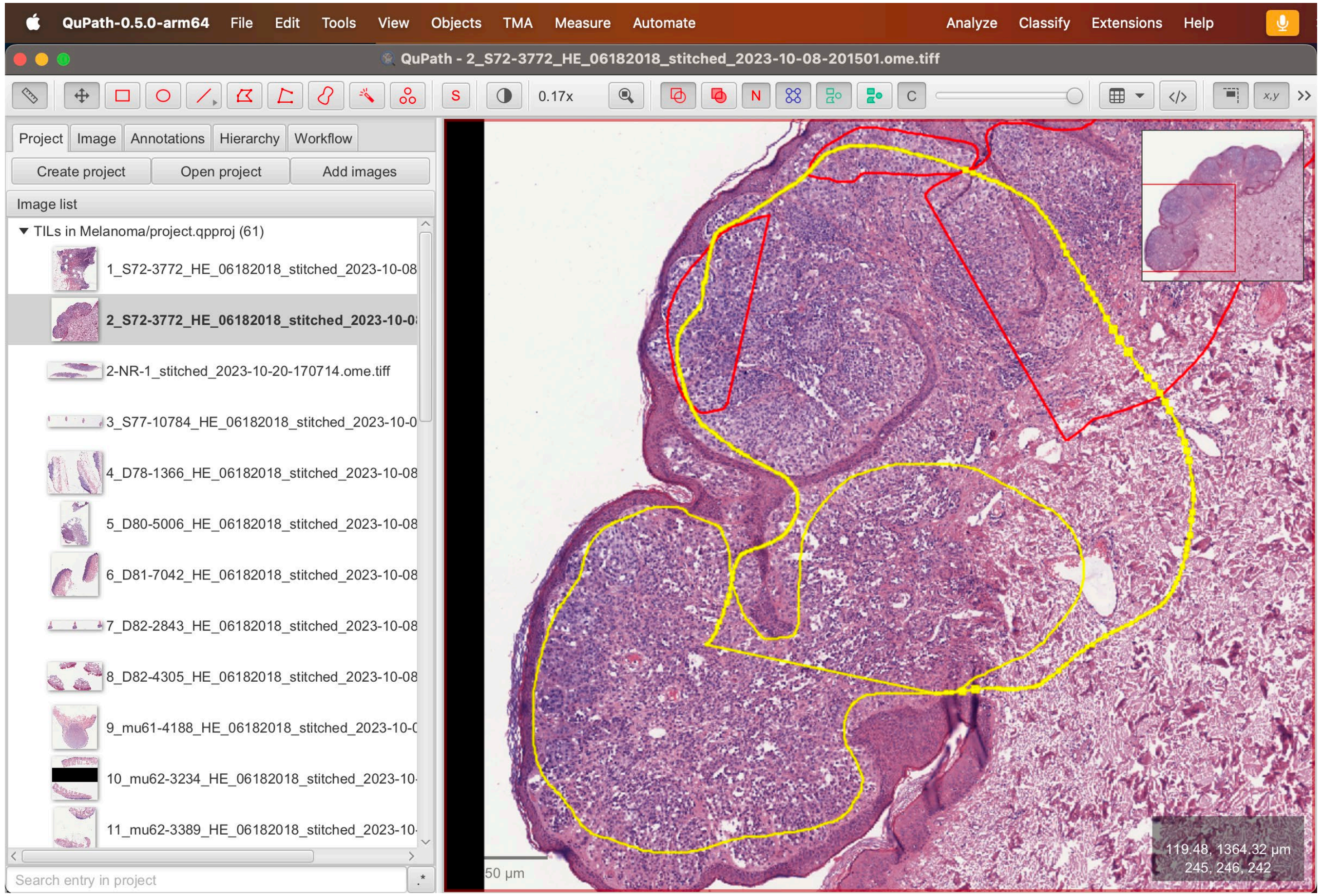

© 2025 Aung TN et al. *JAMA Network Open*.

Step 5: Annotate the Images

***Tip 2:** For the purposes of this study, try to avoid including squamous epithelium, tumor necrosis, ulceration. Stroma can be judiciously included if they are within 250 microns of the tumor.*

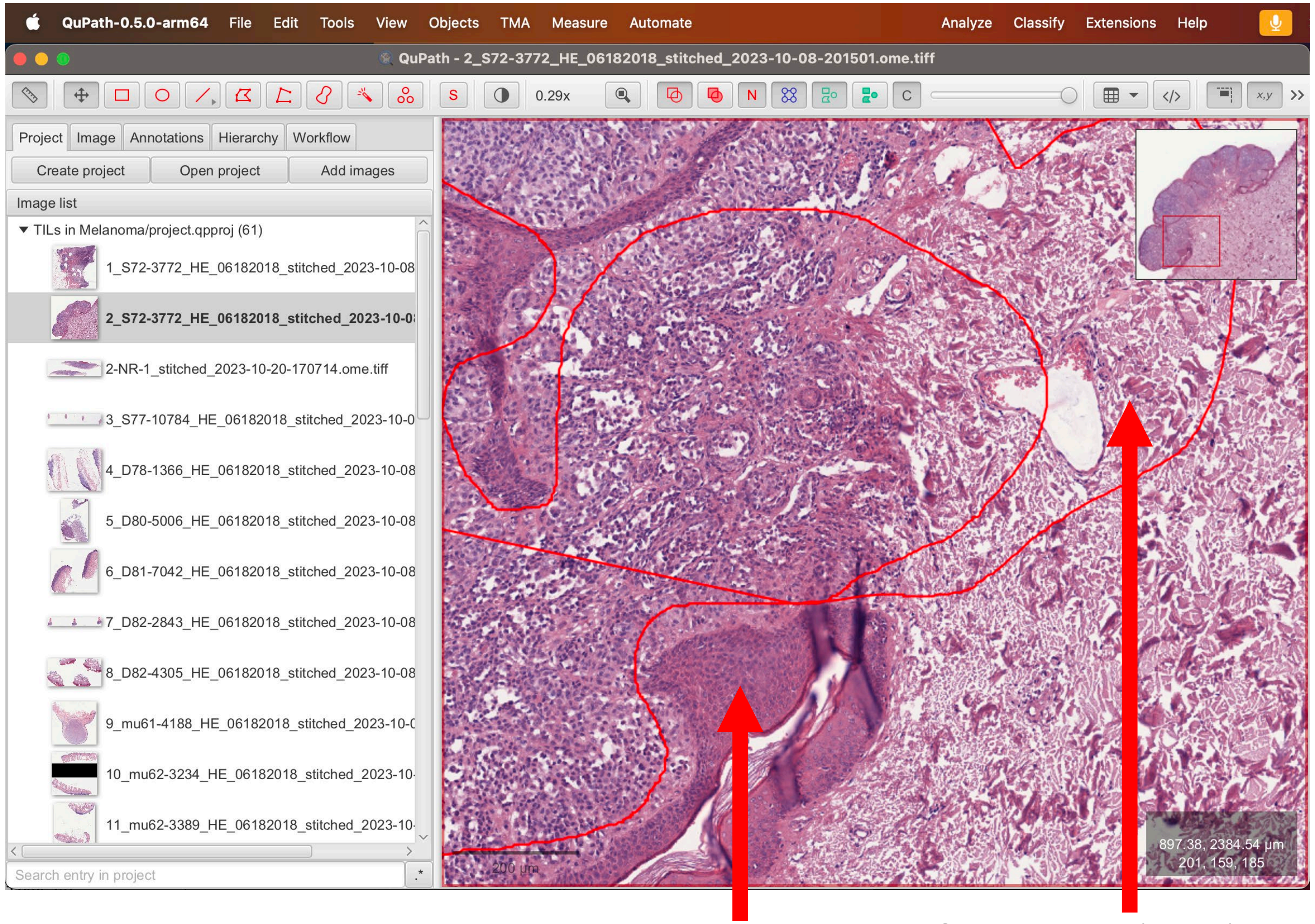

Avoid squamous epithelium

Some stromal inclusion (max 1mm) OK

© 2025 Aung TN et al. *JAMA Network Open*.

Step 5: Annotate the Images

Tip 3: *If desired, you can force certain areas to be ignored. This can be helpful if you overdrew an annotation – instead of redrawing it, you can overlay the undesired portion with another annotation. Right click on that annotation, go to "Set classification" → "Ignore*". This will designate this as a special annotation to exclude the area overlapping with your intended ROI annotation(s).*

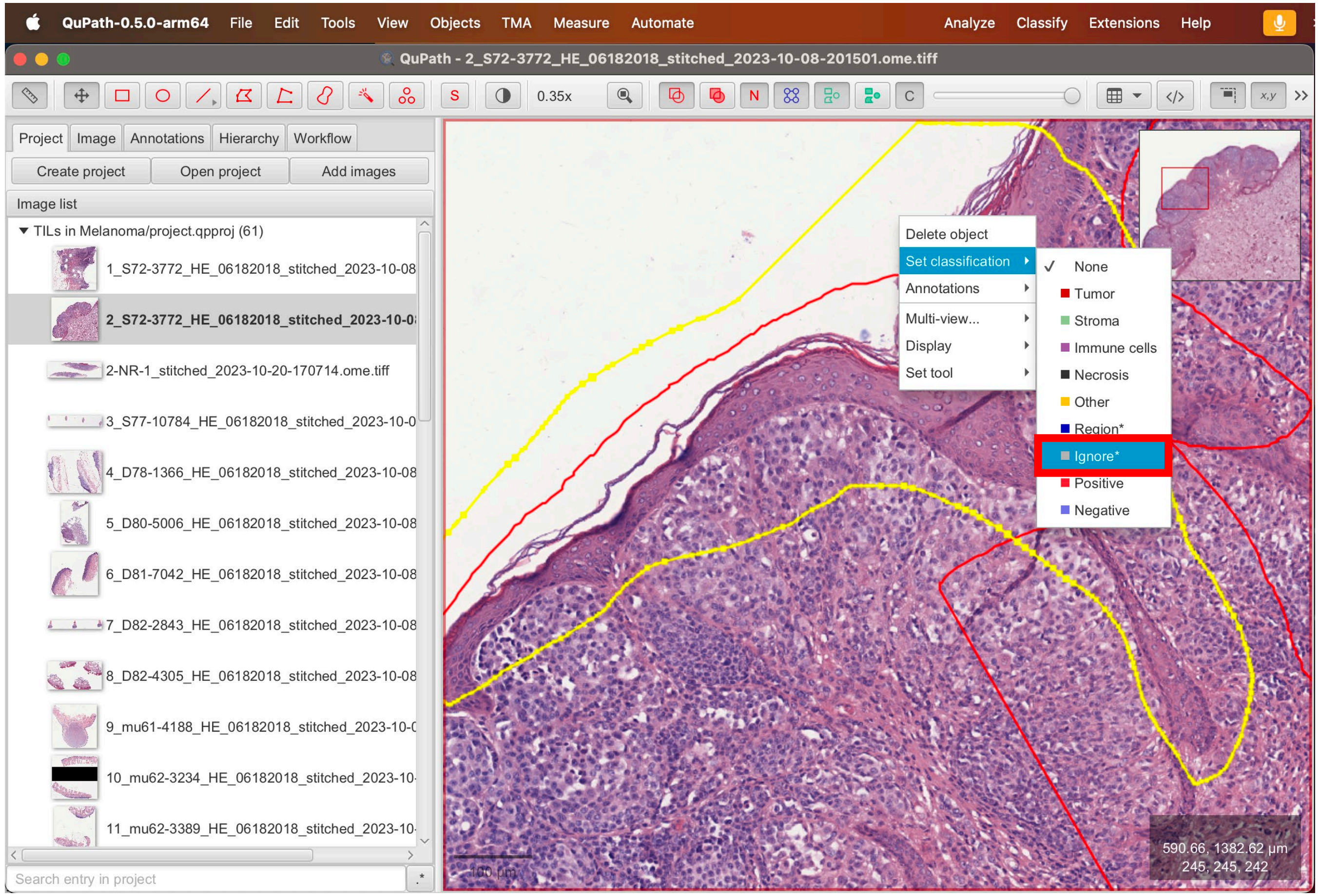

© 2025 Aung TN et al. *JAMA Network Open.*

Step 5: Annotate the Images

3. Save each image as you go through them and create ROIs.

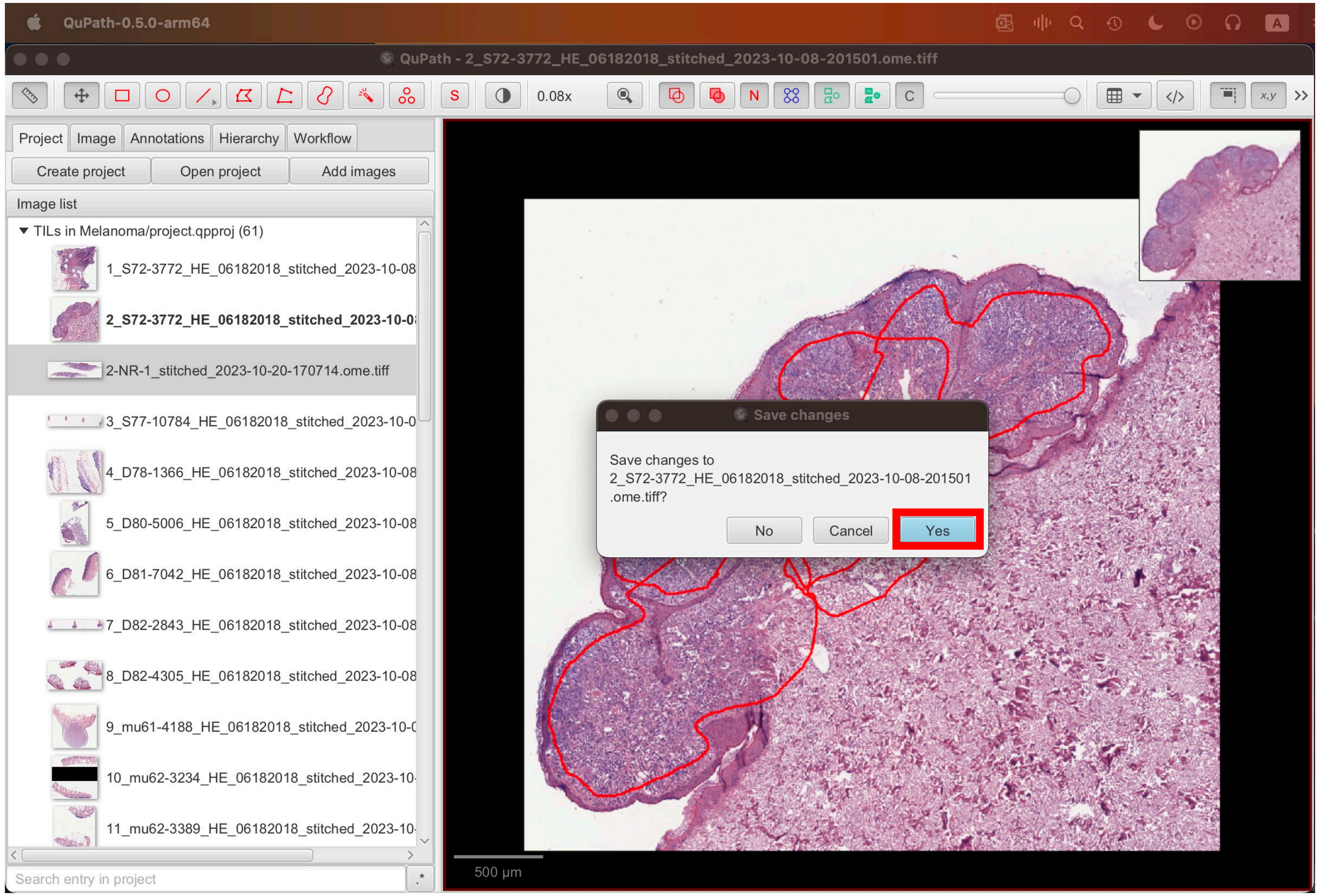

© 2025 Aung TN et al. *JAMA Network Open.*

Step 6: Upload the Project

1. After completion, upload the fully annotated project here.
   - This should be the folder where you created your QuPath project. Please just upload this and *not* the associated images you also downloaded.

© 2025 Aung TN et al. *JAMA Network Open.*

Step 7: Run the Script

1. Going back to your project, drag the provided script onto the interface.

- The script window should pop up.

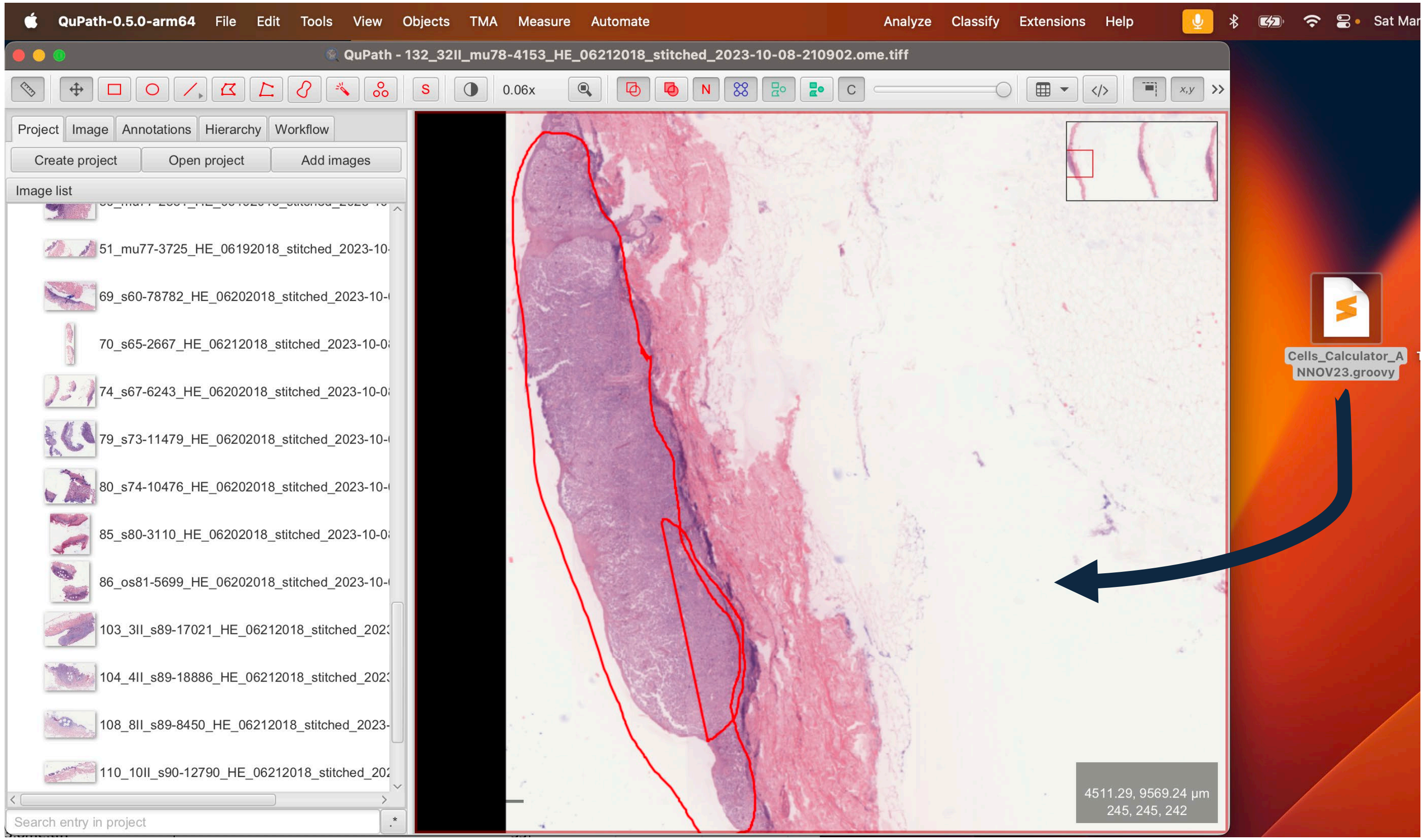

© 2025 Aung TN et al. *JAMA Network Open.*

Step 7: Run the Script

2. Click on the script window. Then select "Run" → "Run for Project".

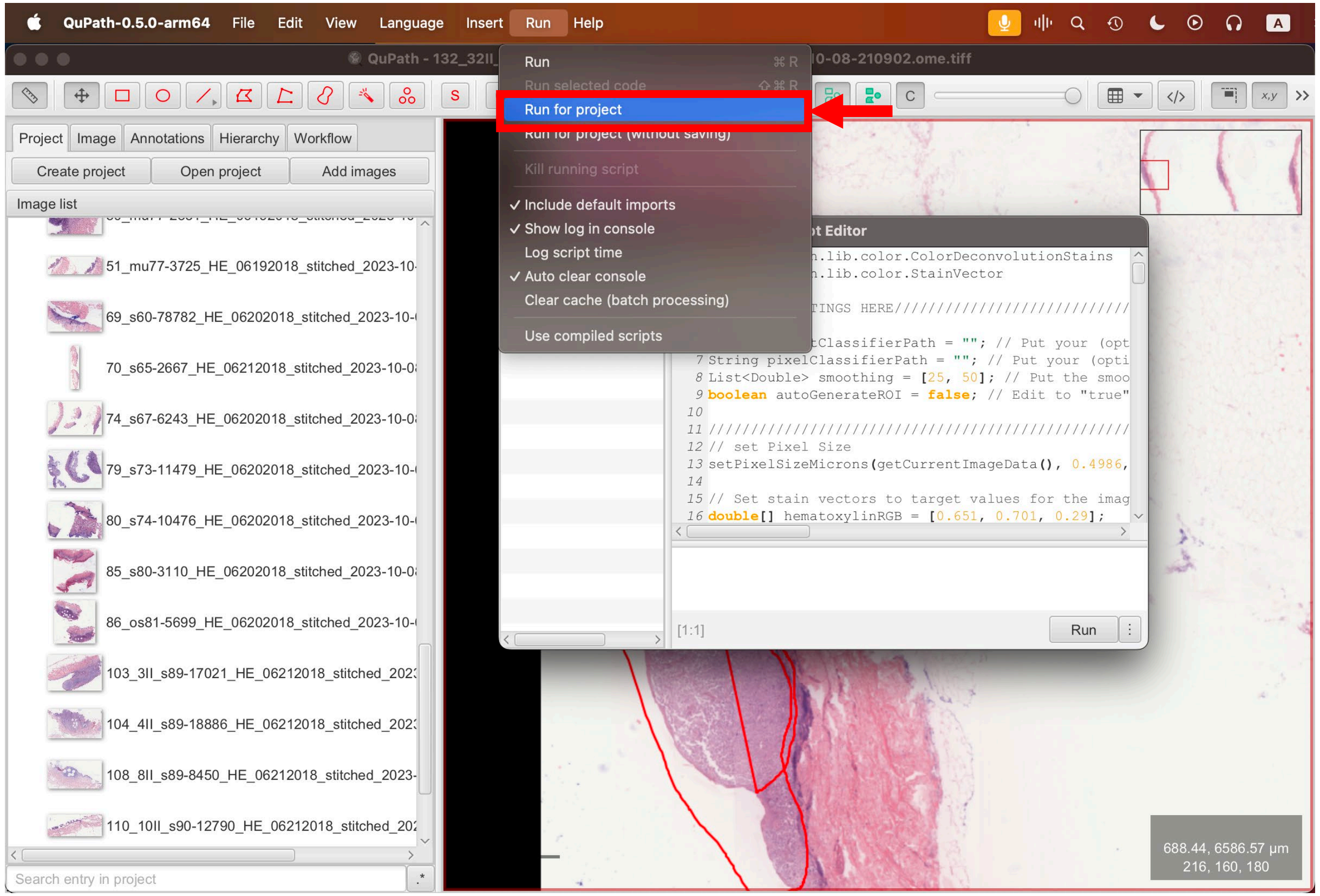

© 2025 Aung TN et al. *JAMA Network Open.*

Step 7: Run the Script

3. In the next pop up window, click on the ">>" button to include all images to be run. Then click "OK" to run the script.

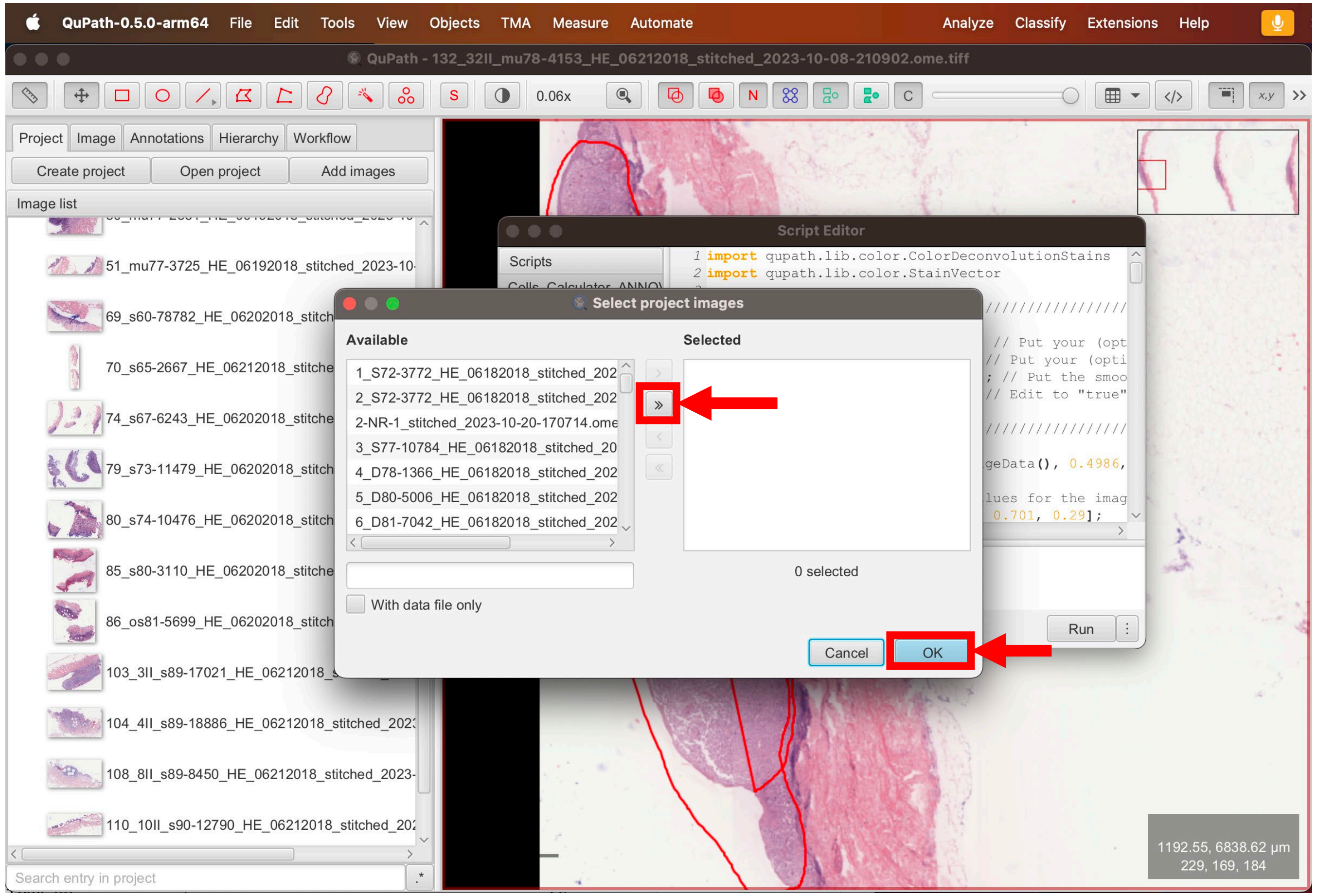

Note: Please make sure at this point ALL your images have all changes saved.

© 2025 Aung TN et al. *JAMA Network Open.*

Step 7: Run the Script

4. Wait for the script to complete.
    - This may take some time...

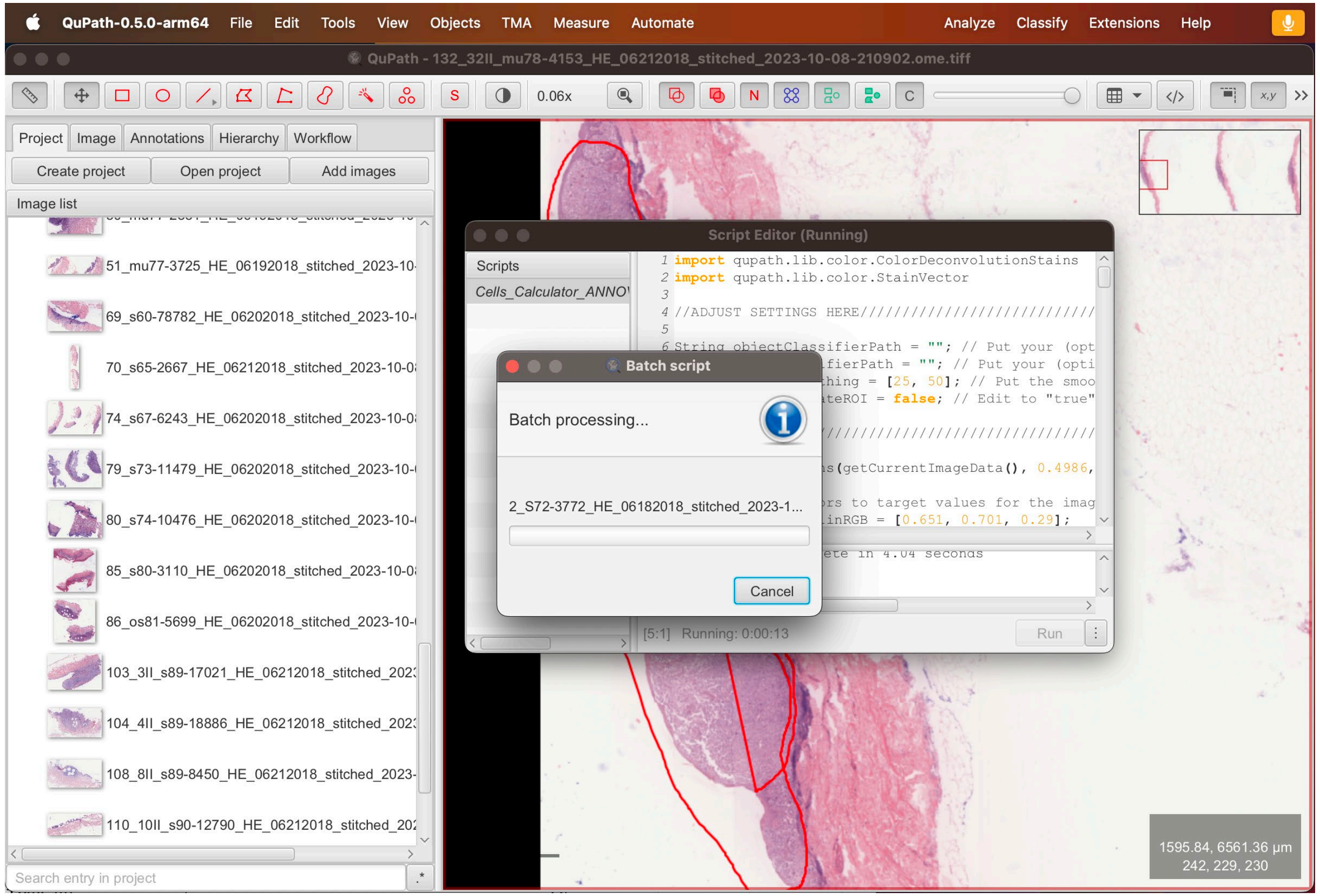

© 2025 Aung TN et al. *JAMA Network Open.*

Step 8: Export Measurements

1. Once the script has finished. Go back to your project and select “Measure” → “Export measurements”

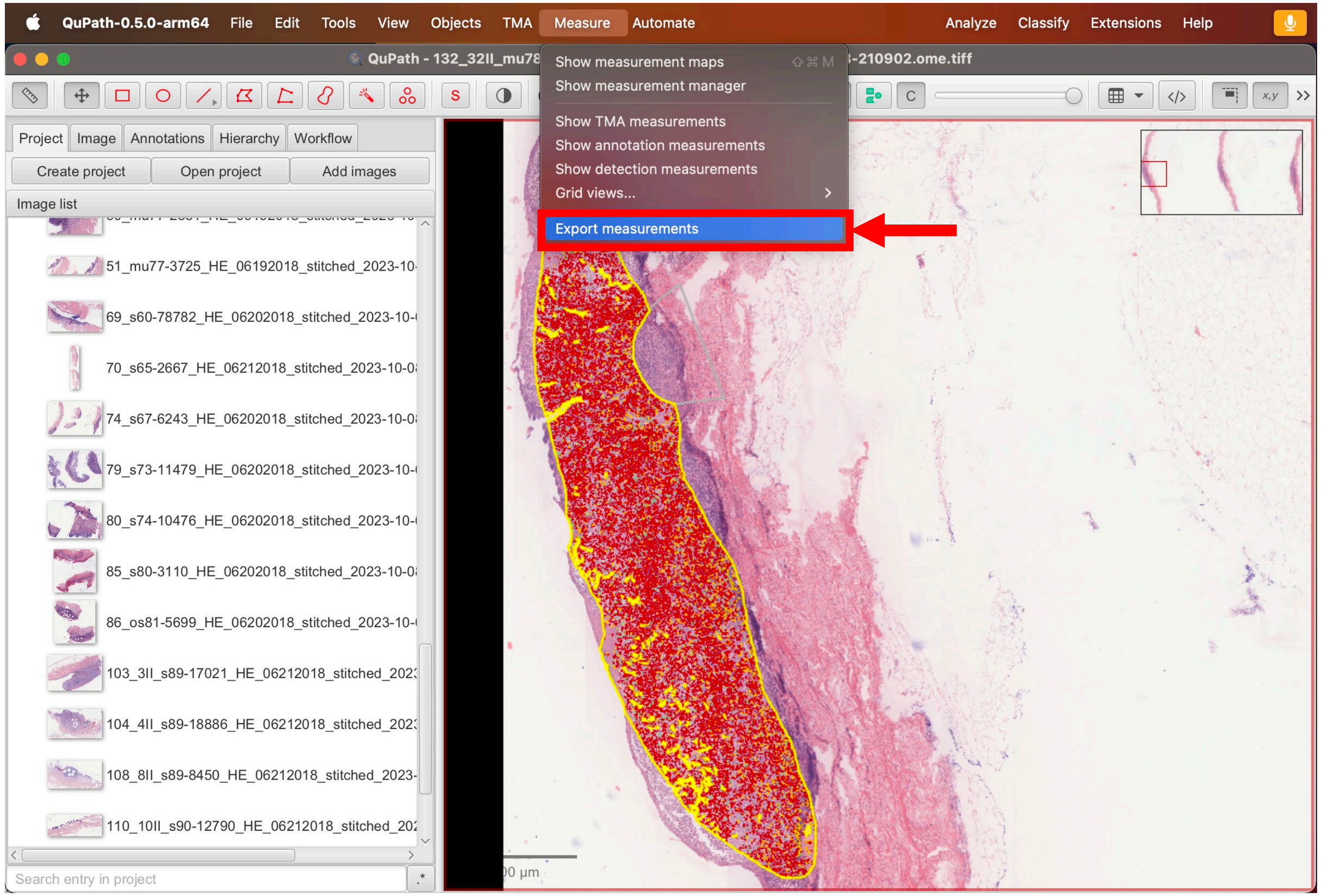

© 2025 Aung TN et al. *JAMA Network Open.*

Step 8: Export Measurements

2. In the pop-up window, follow the settings shown on the right.

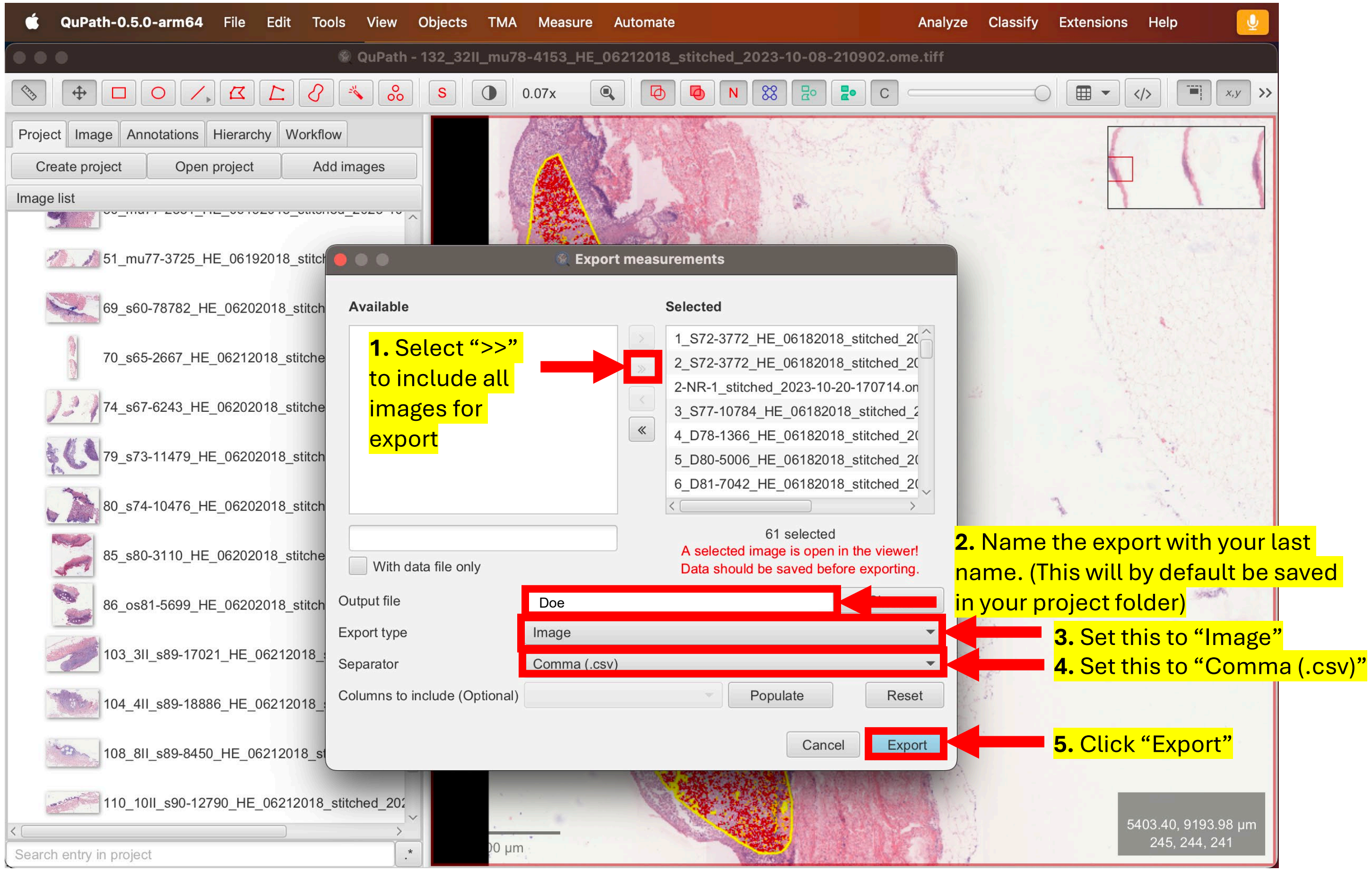

© 2025 Aung TN et al. *JAMA Network Open.*

eAppendix 2

Instructions for Manual TIL Scoring

TILs Melanoma Study

© 2025 Aung TN et al. *JAMA Network Open.*

Step 1: Download the H&E Images

1. Click here to find the images on Box.
    - You will be prompted to log into to your Box account (or make an account if needed).

© 2025 Aung TN et al. *JAMA Network Open.*

Step 1: Download the H&E Images

2. Select the folder containing the images, right click, and click ”Download”.

- This will be a heavy download. Make sure your computer or drive has at least 100 GB.

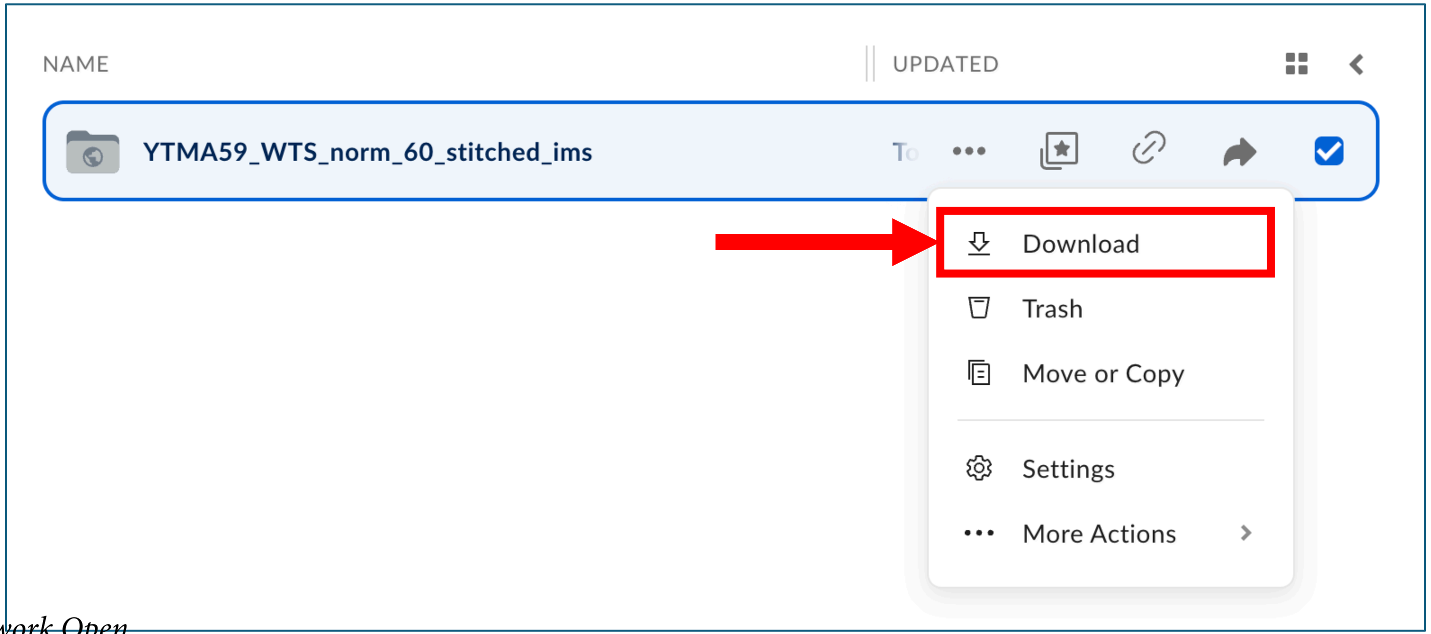

© 2025 Aung TN et al. *JAMA Network Open.*

Step 1: Download the H&E Images

3. Once downloaded (and extracted as needed), keep the images in a safe location in your computer.

- We recommend putting them in a location where they won't be changed or removed.

© 2025 Aung TN et al. *JAMA Network Open.*

The next instructions will cover the installation of QuPath.

QuPath is an open-source slide viewer software we recommend to view the provide H&E images.

However, please feel free to use any slide viewer software of your choice, in which case, you can skip to Step 5 (and take any mention of QuPath as the software you are using).

© 2025 Aung TN et al. *JAMA Network Open.*

Step 2: Install QuPath

1. Download the latest version of QuPath here, according to your system.

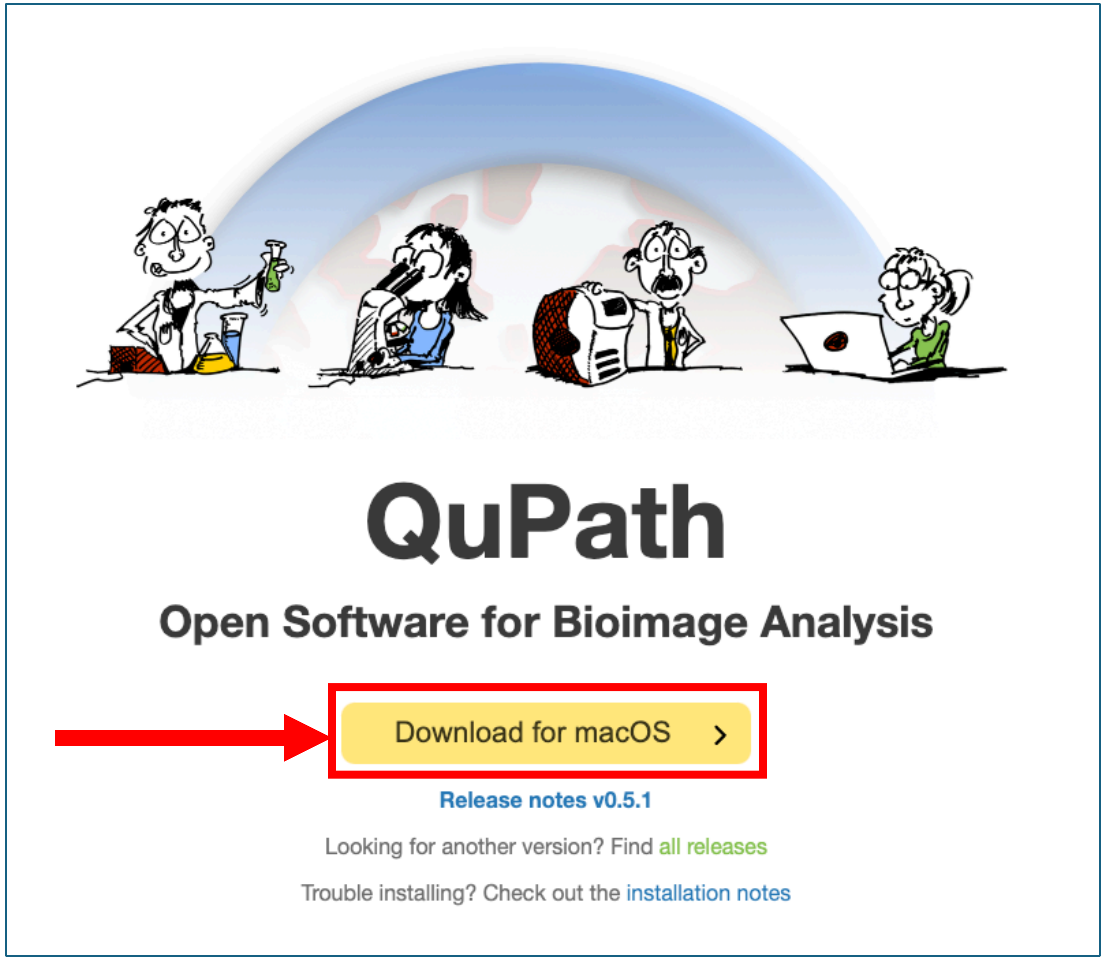

© 2025 Aung TN et al. *JAMA Network Open.*

Step 2: Install QuPath

2. Open the downloaded package and follow the installation instructions (may vary depending on system).

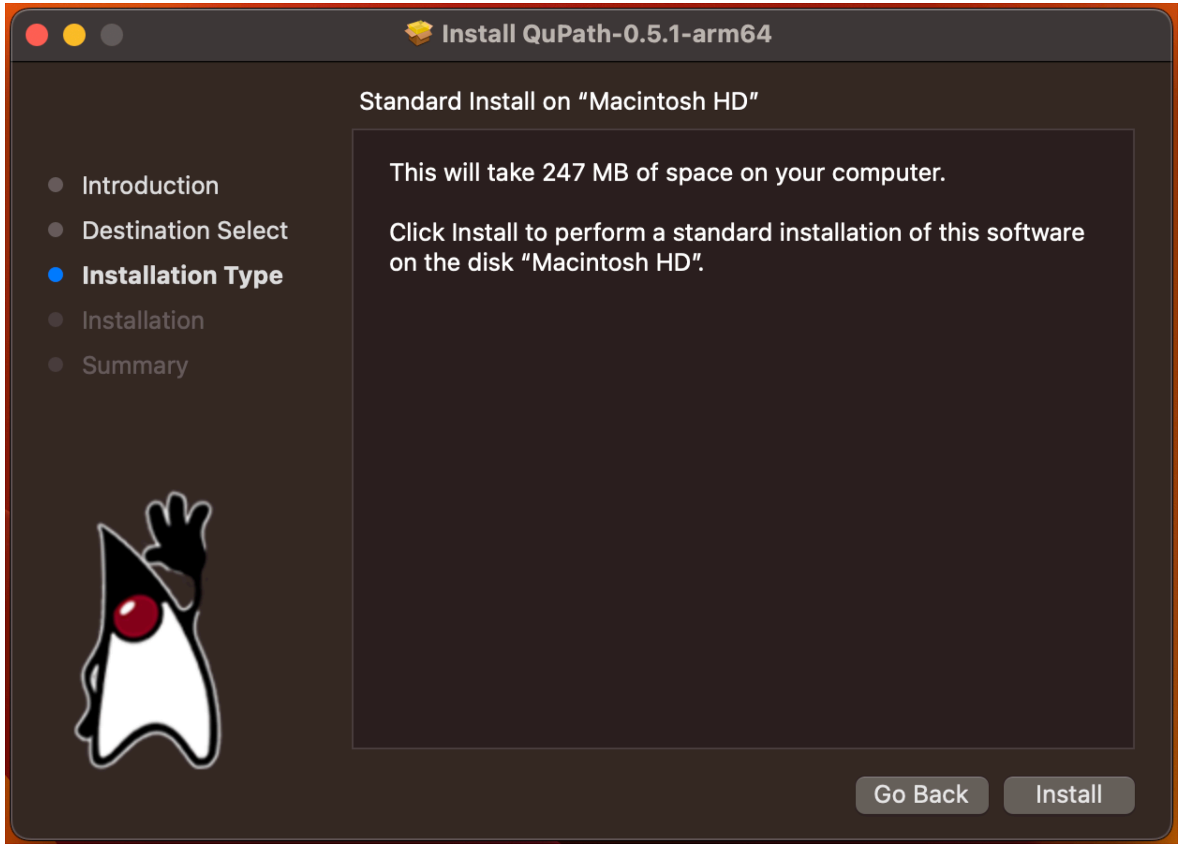

© 2025 Aung TN et al. *JAMA Network Open.*

Step 3: Create a QuPath Project

1. Open QuPath

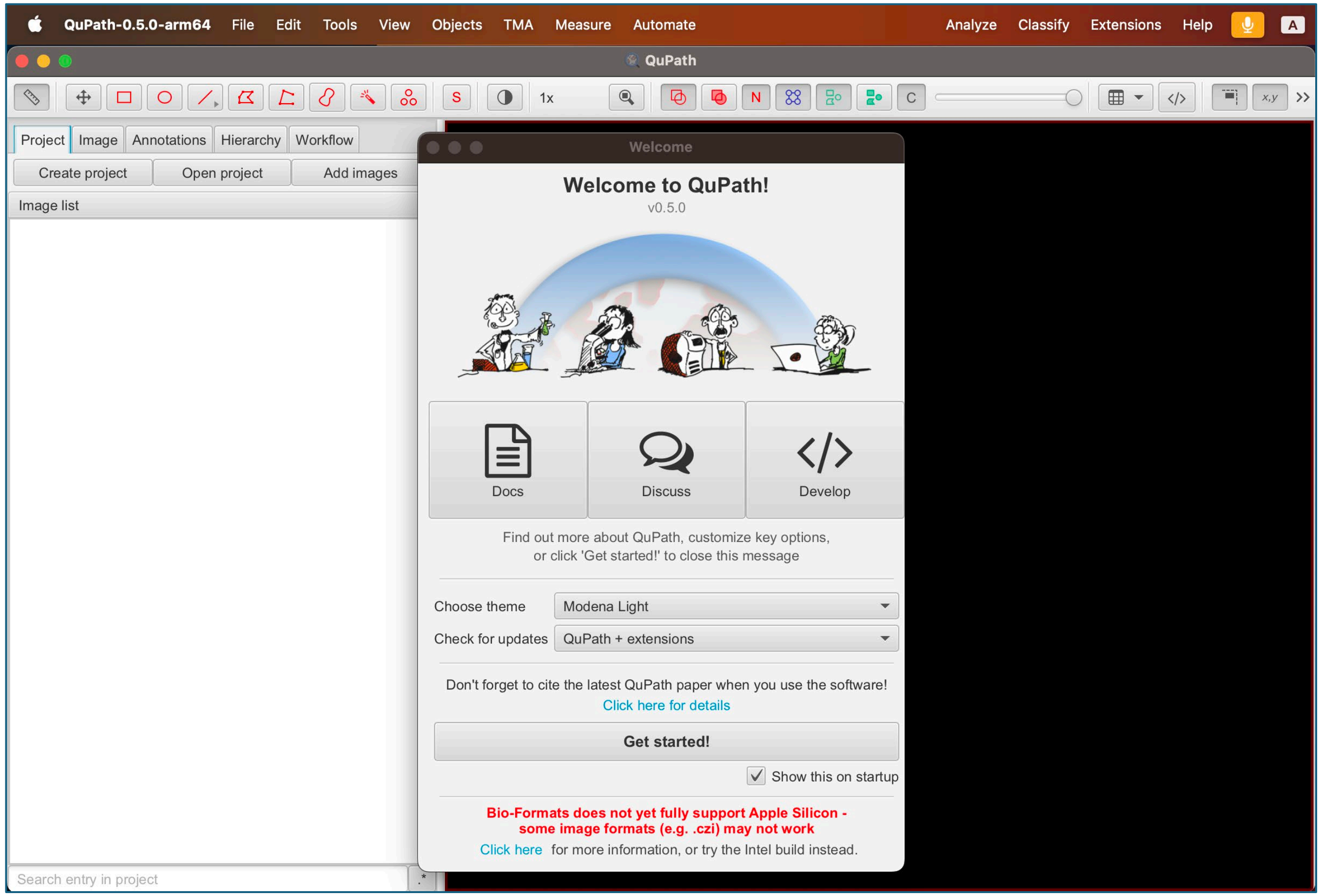

© 2025 Aung TN et al. *JAMA Network Open*.

Step 3: Create a QuPath Project

2. Create a new project by clicking on ”File” → “Project...” → “Create Project”

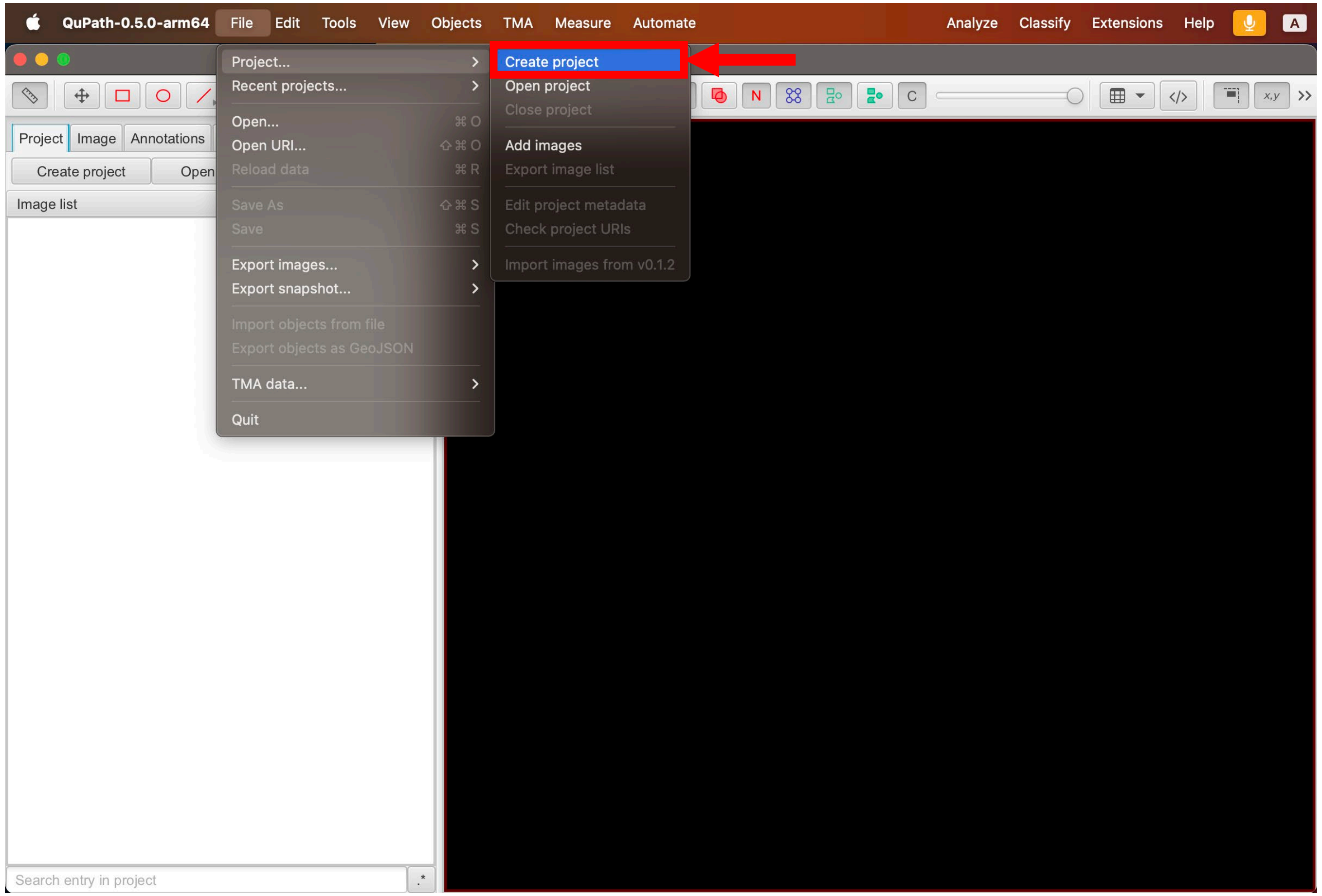

© 2025 Aung TN et al. *JAMA Network Open.*

Step 3: Create a QuPath Project

3. In the pop-up window, select an empty folder (create one if you haven't already) to finish creating your project.

(Note: Like for the images, it's best to have your project folder in a safe location.)

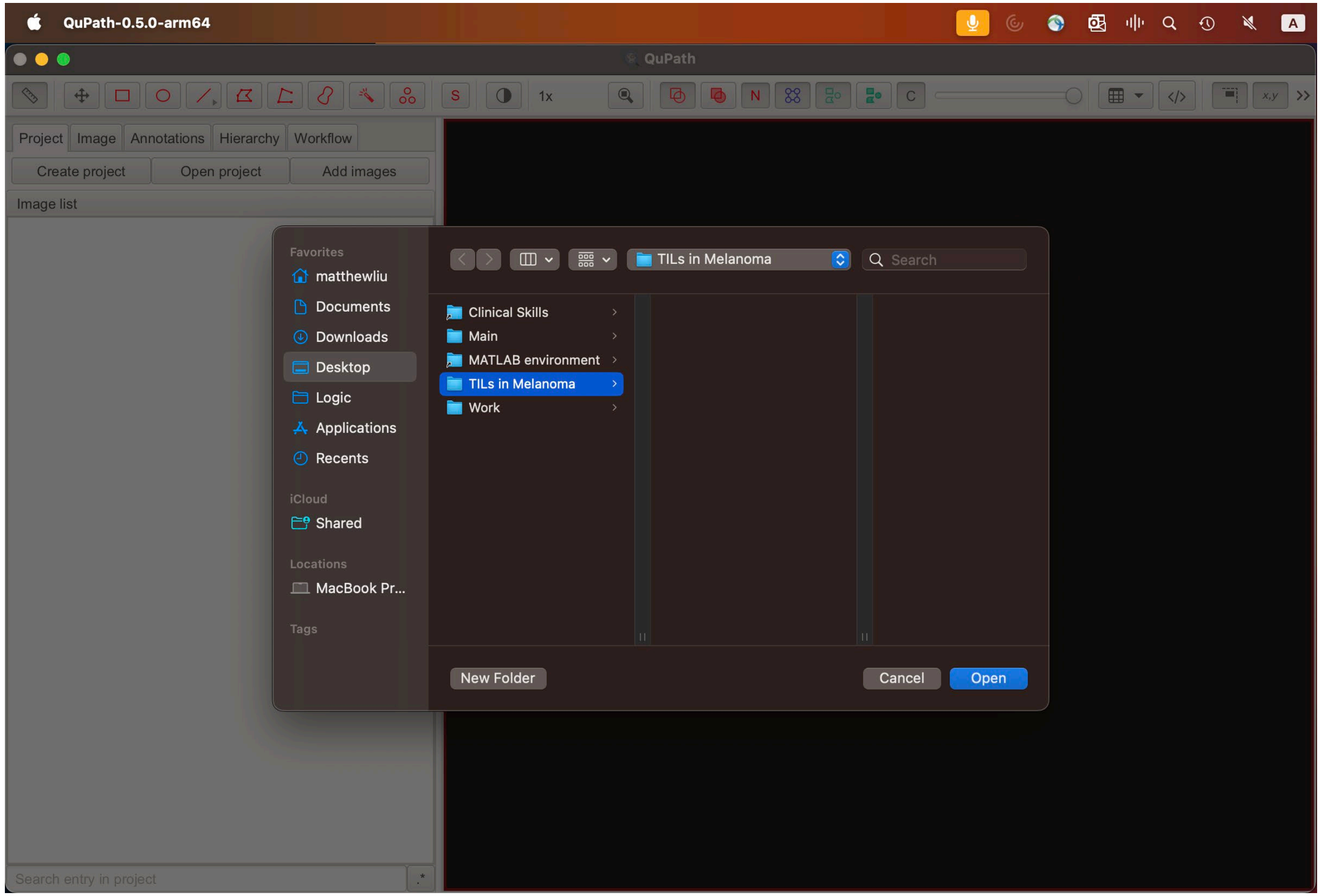

© 2025 Aung TN et al. *JAMA Network Open.*

Step 4: Load the images

1. Take the images you downloaded and drag them onto your QuPath project.

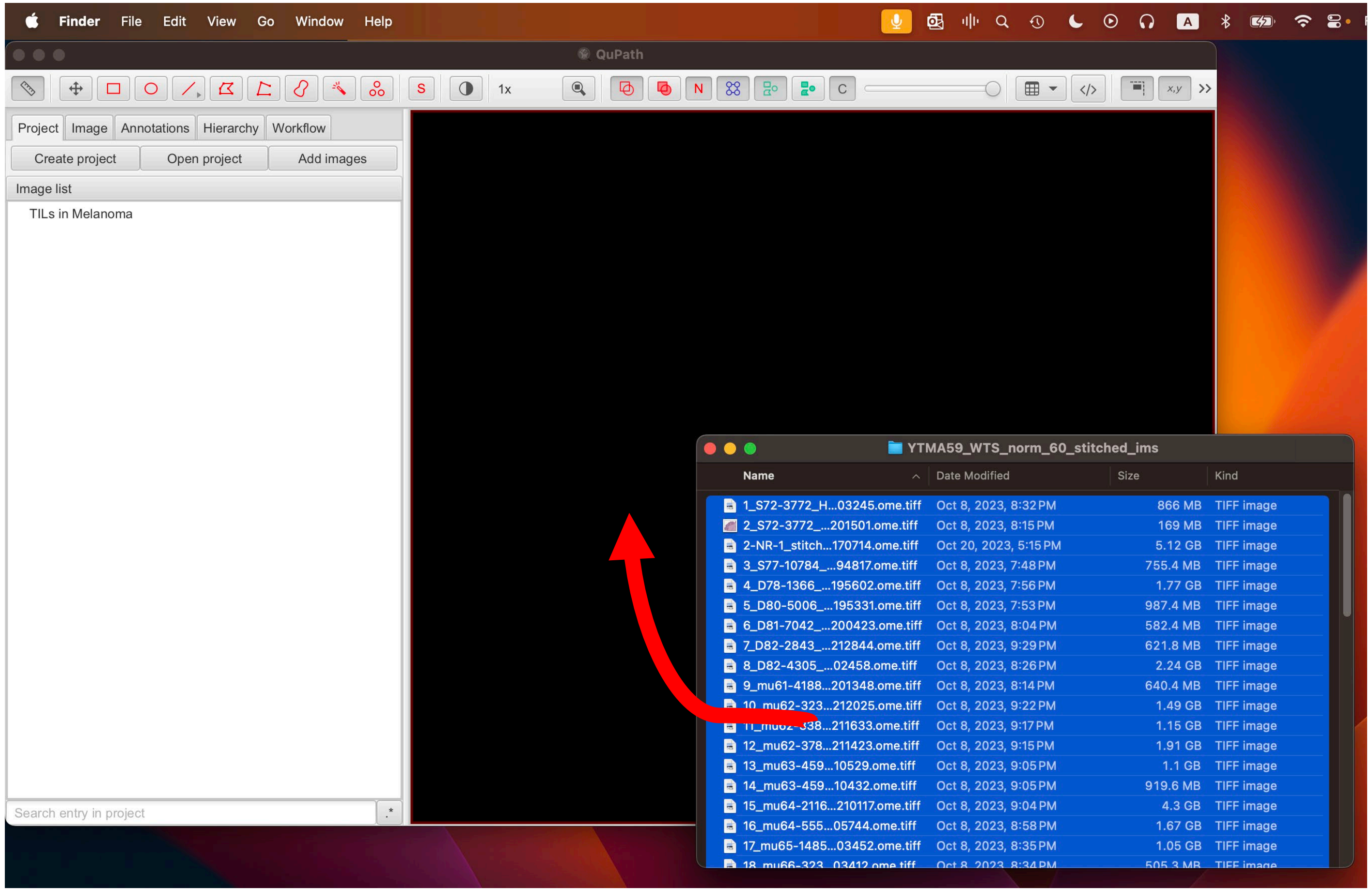

© 2025 Aung TN et al. *JAMA Network Open*.

Step 4: Load the images

2. In the pop-up prompt, simply set the image type to "Brightfield (H&E)" and then click "Import"

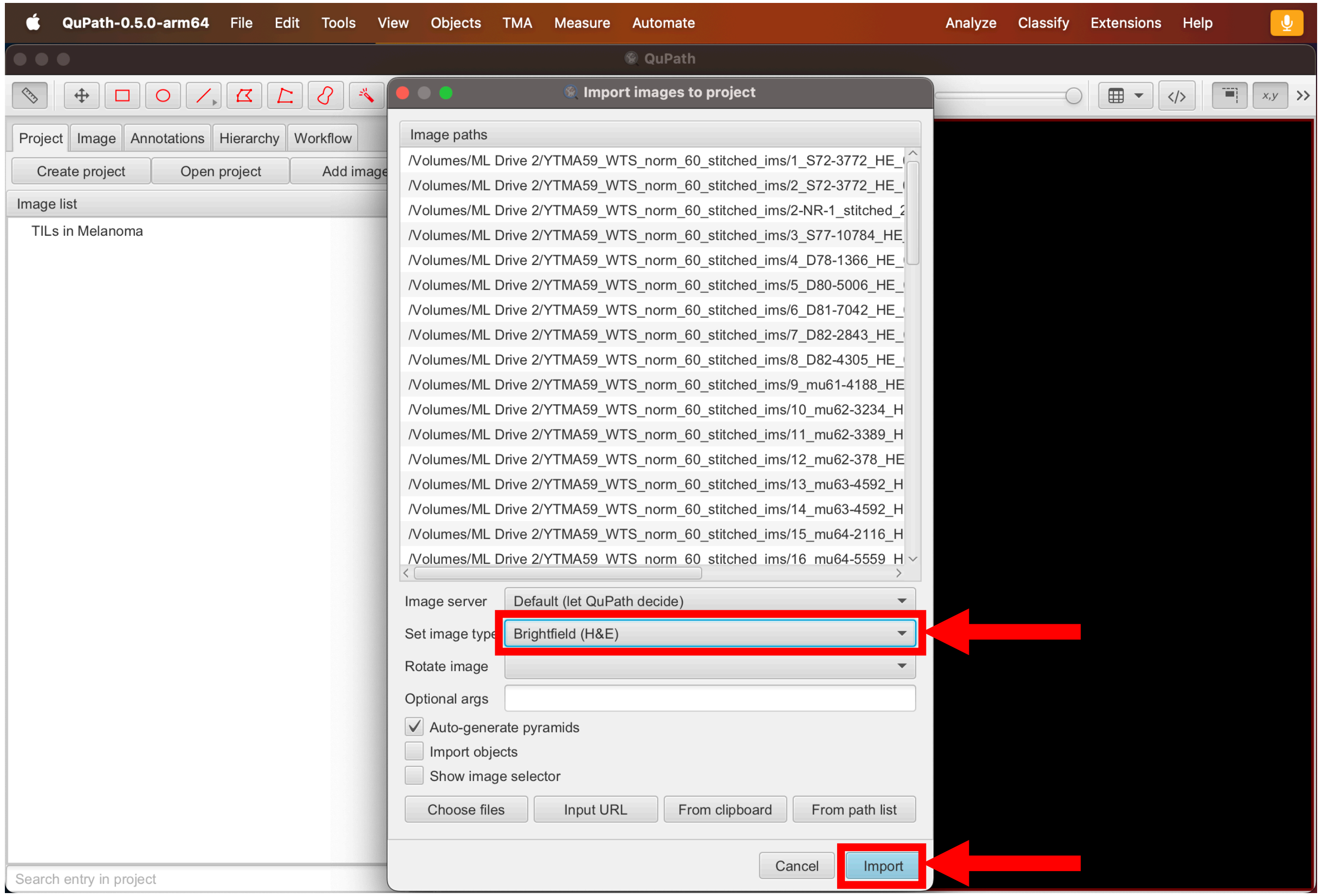

© 2025 Aung TN et al. *JAMA Network Open.*

Step 5: Read and Score the Images

1. While you have your QuPath project still open, open the Excel scoring spreadsheet we provided you.

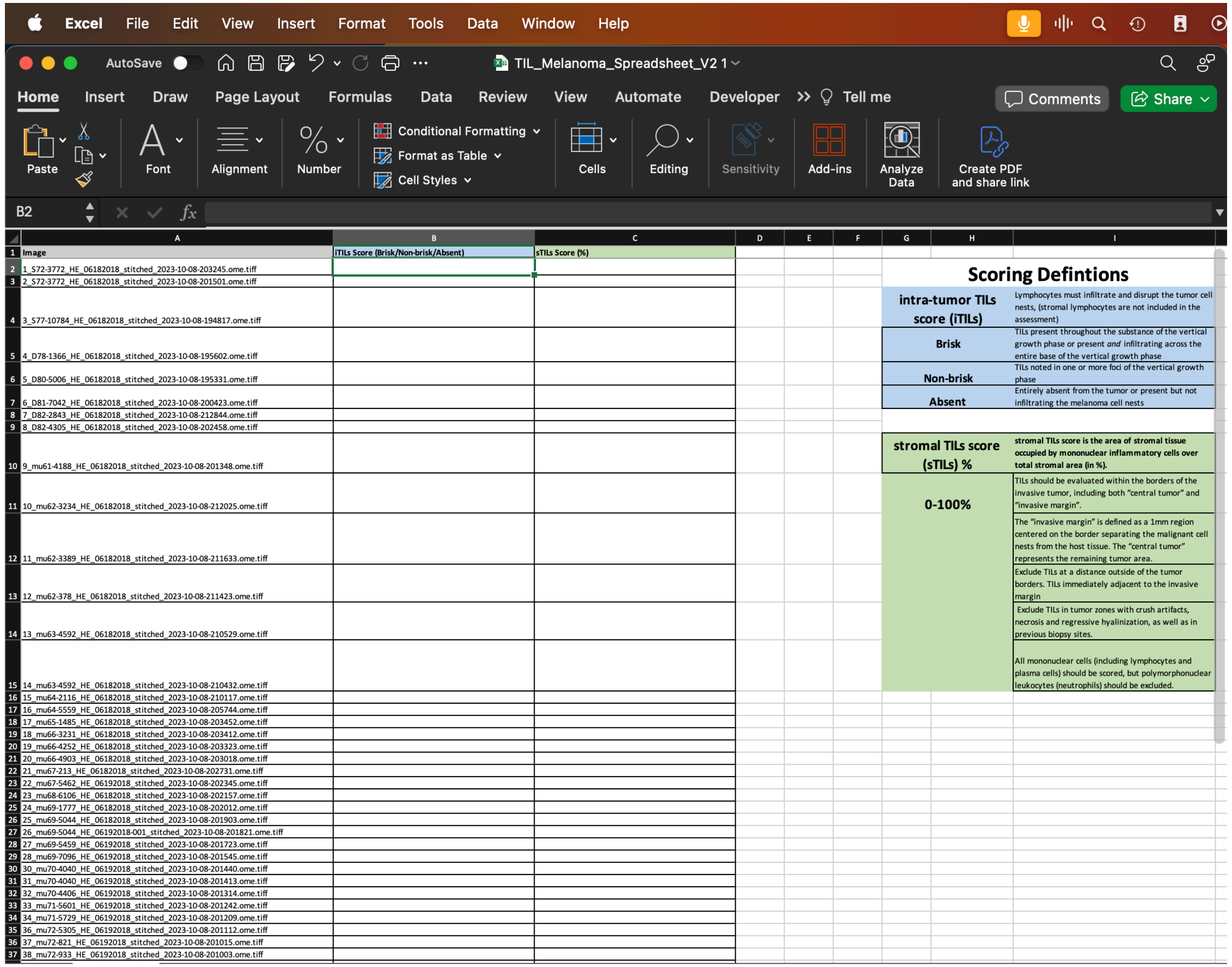

© 2025 Aung TN et al. *JAMA Network Open.*

Step 5: Read and Score the Images

2. Back on QuPath, go through the images.

- You can navigate by double-clicking on the images on the side pane.

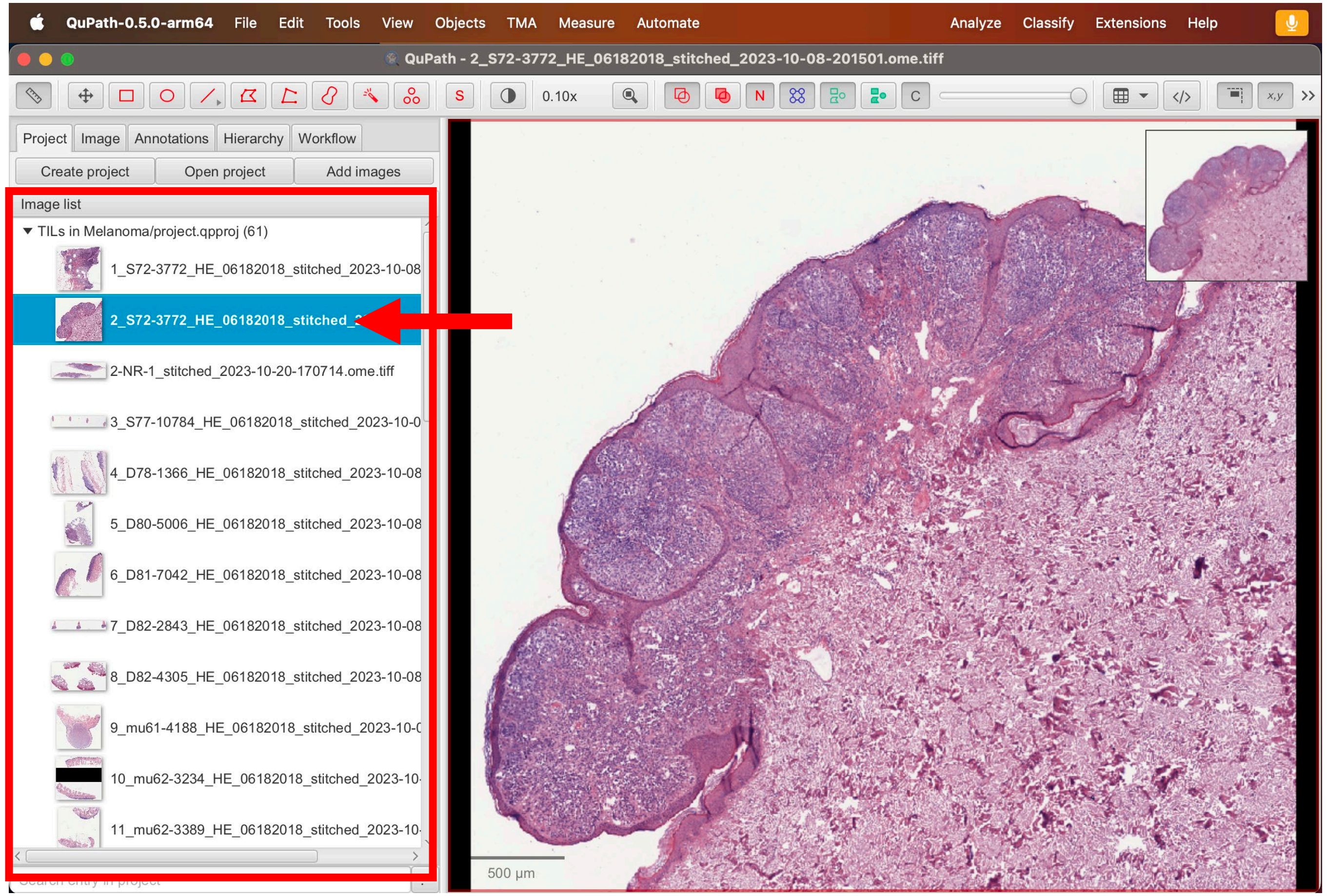

© 2025 Aung TN et al. *JAMA Network Open.*

Step 5: Read and Score the Images

3. Read at each image carefully for manual TIL scoring.

- Scroll to zoom in/out.
- Click and drag to move the image around.

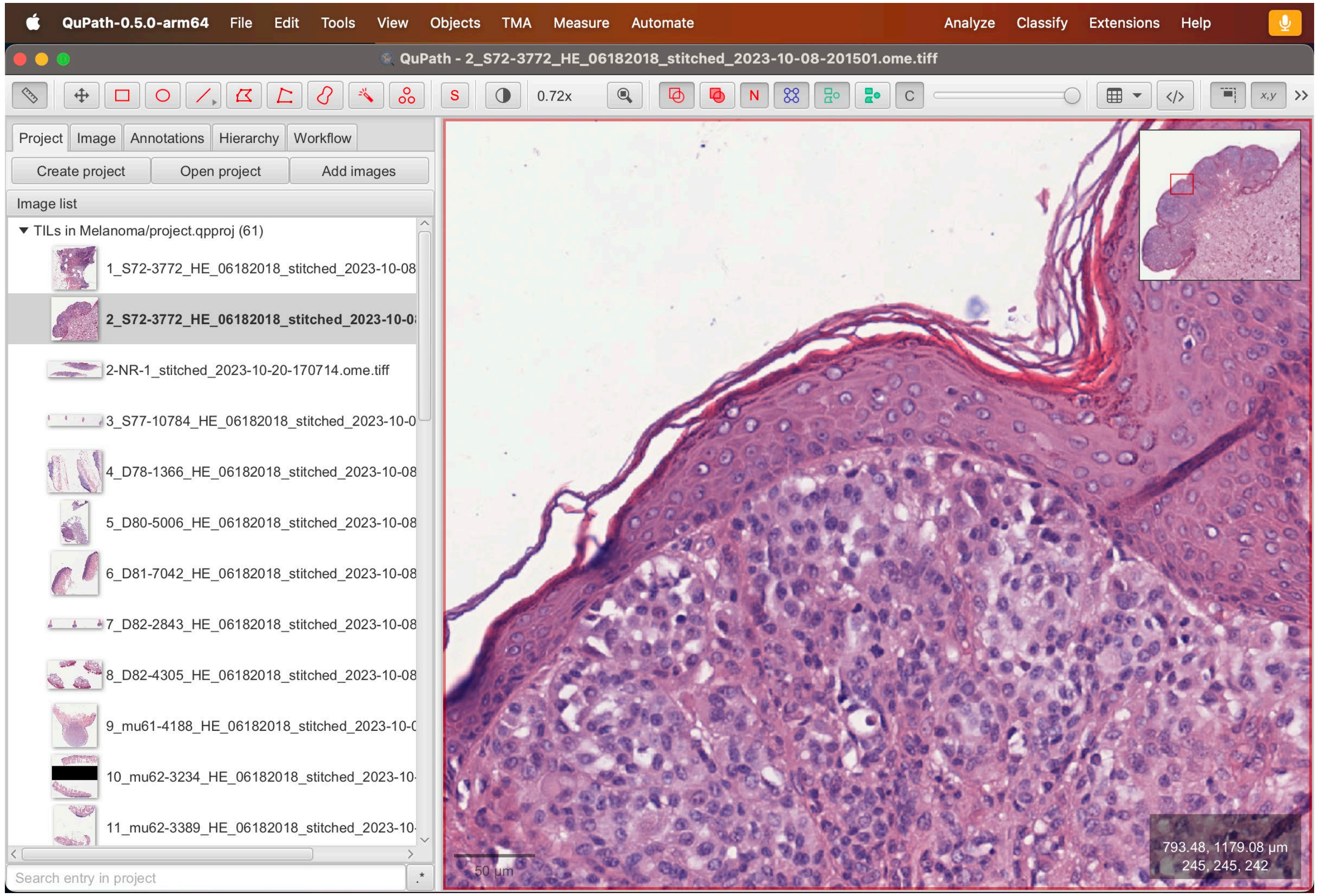

© 2025 Aung TN et al. *JAMA Network Open.*

Step 5: Read and Score the Images

4. Determine the localization of inflammatory infiltrate.

- Score intra-tumor TILs (iTILs) according to the Clark system: brisk / non-brisk / absent categories (1,2)
- Score stromal TILs (sTILs) according to the TIL WG guidelines (3). Refer to the sTILs supplementary for details

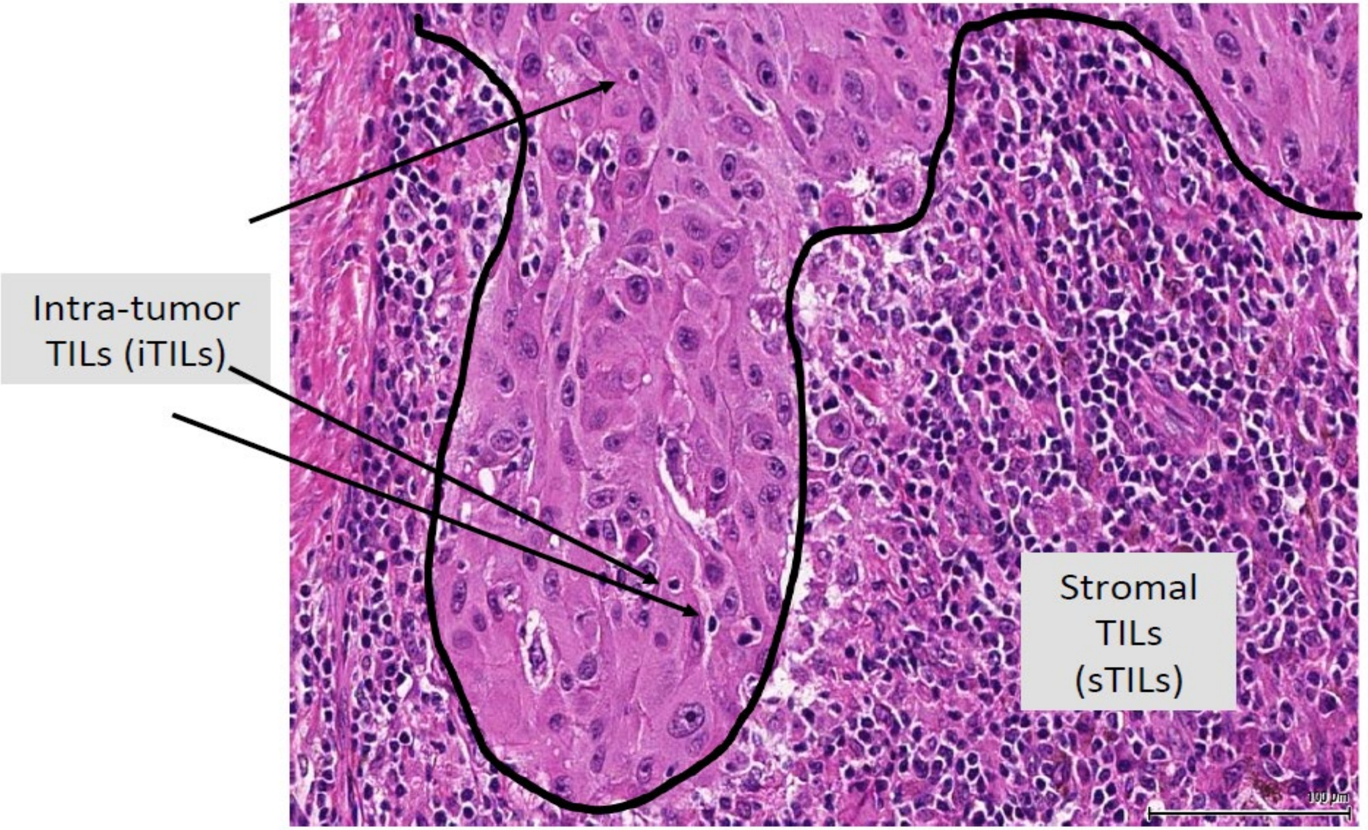

1, Elder DE et al Cancer 1985
2, Clark WH et al J Natl Cancer Inst 1989
3, Hendry S et al Adv Anat Pathol 2017

© 2025 Aung TN et al. JAMA Network Open.

Step 5: Read and Score the Images

5. Input your score beside the corresponding entry on the Excel sheet.

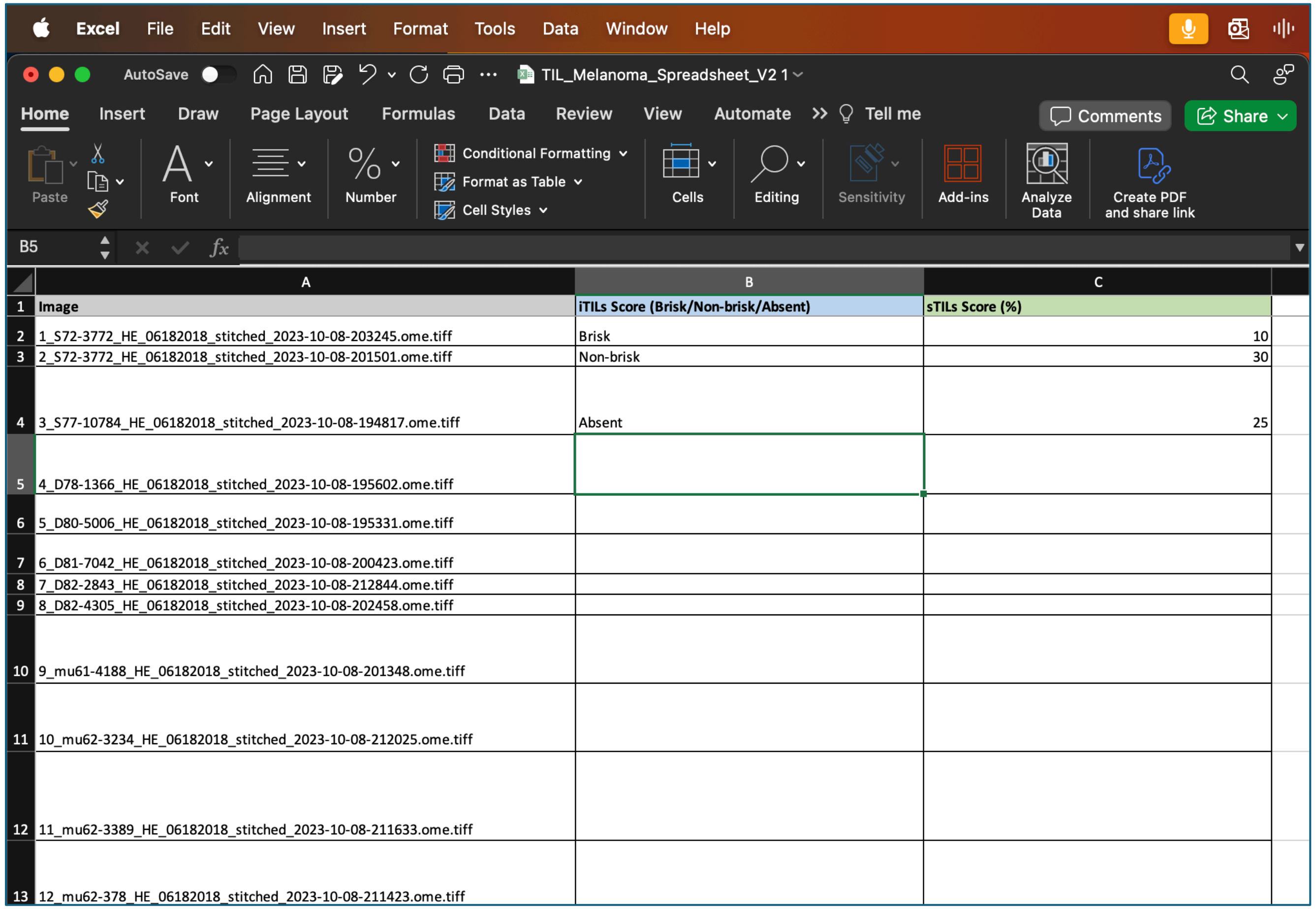

	A	B	C
1	Image	iTILs Score (Brisk/Non-brisk/Absent)	sTILs Score (%)
2	1_S72-3772_HE_06182018_stitched_2023-10-08-203245.ome.tiff	Brisk	10
3	2_S72-3772_HE_06182018_stitched_2023-10-08-201501.ome.tiff	Non-brisk	30
4	3_S77-10784_HE_06182018_stitched_2023-10-08-194817.ome.tiff	Absent	25
5	4_D78-1366_HE_06182018_stitched_2023-10-08-195602.ome.tiff		
6	5_D80-5006_HE_06182018_stitched_2023-10-08-195331.ome.tiff		
7	6_D81-7042_HE_06182018_stitched_2023-10-08-200423.ome.tiff		
8	7_D82-2843_HE_06182018_stitched_2023-10-08-212844.ome.tiff		
9	8_D82-4305_HE_06182018_stitched_2023-10-08-202458.ome.tiff		
10	9_mu61-4188_HE_06182018_stitched_2023-10-08-201348.ome.tiff		
11	10_mu62-3234_HE_06182018_stitched_2023-10-08-212025.ome.tiff		
12	11_mu62-3389_HE_06182018_stitched_2023-10-08-211633.ome.tiff		
13	12_mu62-378_HE_06182018_stitched_2023-10-08-211423.ome.tiff		

For illustration purposes only. Not to be interpreted as real data.

© 2025 Aung TN et al. JAMA Network Open.

Step 5: Read and Score the Images

6. Once you have completed the Excel sheet, select "Save As". Name this version with your own initials.

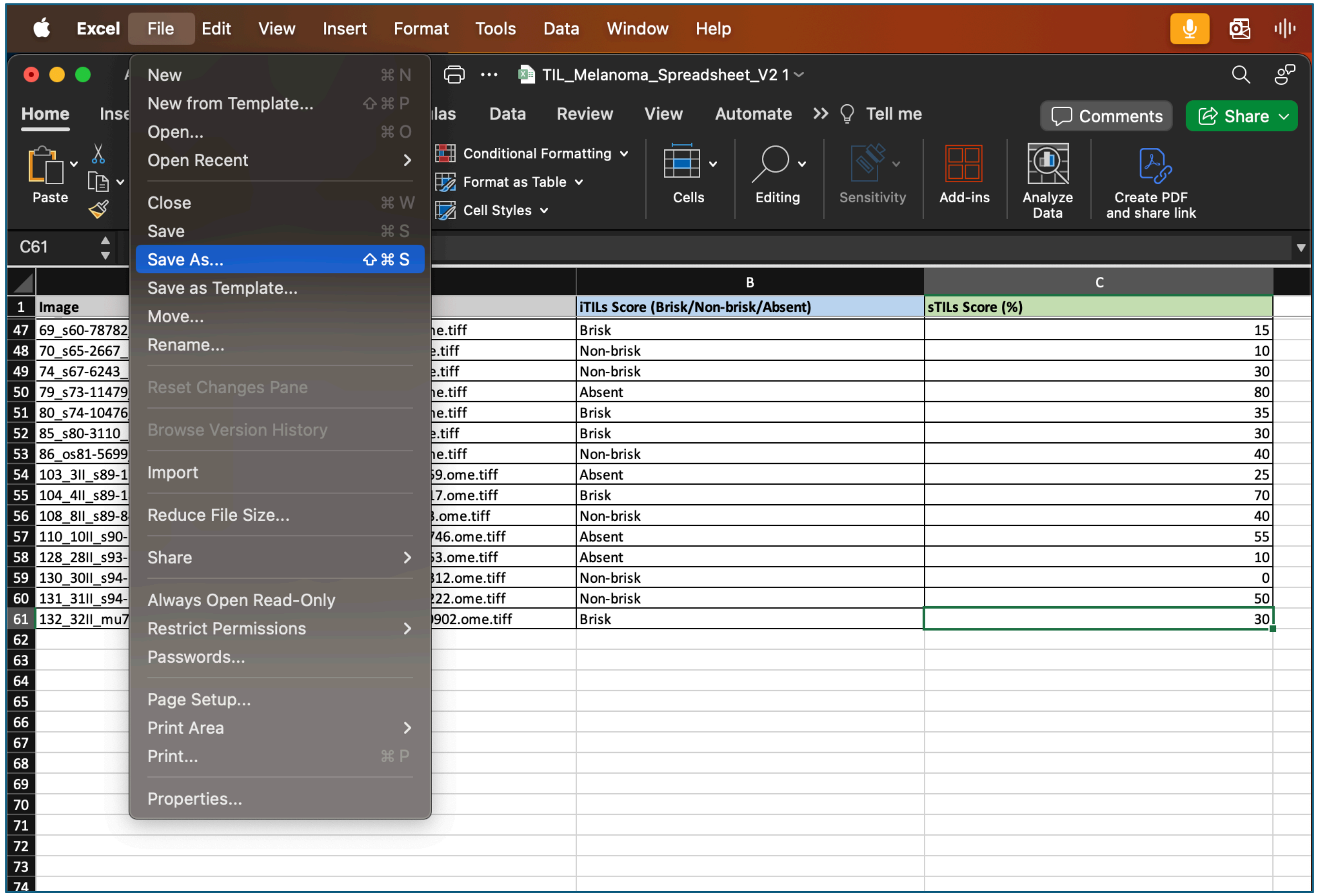

For illustration purposes only. Not to be interpreted as real data.

© 2025 Aung TN et al. *JAMA Network Open.*

Step 6: Upload the Excel Spreadsheet

1. Upload the Excel spreadsheet here.

© 2025 Aung TN et al. *JAMA Network Open.*
