# Supplementary material for: Pathologist-Read vs AI-Driven Assessment of Tumor-Infiltrating Lymphocytes in Melanoma
Source: JAMA Netw Open. 2025 Jul 3;8(7):e2518906. doi: 10.1001/jamanetworkopen.2025.18906 (PMC12232186; doi:10.1001/jamanetworkopen.2025.18906)
Supplement: Supplement 2. — Data Sharing Statement [file jamanetwopen-e2518906-s002.pdf]

Data Sharing Statement

Aung. Analytical and Clinical Validity of Pathologist-Read vs AI-Driven Assessment of Tumor-Infiltrating Lymphocytes in Melanoma. *JAMA Netw Open*. Published July 03, 2025. doi:10.1001/jamanetworkopen.2025.18906

Data
Data available: Yes
Data types: Other (please specify)
Additional Information: The algorithm developed and used in this study has been made publicly available on GitHub (https://github.com/matthewzl/Mel-Color-Norm-TIL-Detection). The images from training cohort are accessible under the accession number S-BIAD470 and those from the testing cohort are accessible upon request from the corresponding authors. The manual and AI-assisted TIL scores can be found in Supplementary Data 1 and 2.
How to access data: https://github.com/matthewzl/Mel-Color-Norm-TIL-Detection
When available: With publication

Supporting Documents
Document types: None

Additional Information
Who can access the data: researchers whose proposed use of the data has been approved
Types of analyses: for specified research purpose
Mechanisms of data availability: after approval of a proposal
